# The dynamics of *N*^6^-methyladenine RNA modification in interactions between rice and plant viruses

**DOI:** 10.1186/s13059-021-02410-2

**Published:** 2021-06-24

**Authors:** Kun Zhang, Xinjian Zhuang, Zhuozhuo Dong, Kai Xu, Xijun Chen, Fang Liu, Zhen He

**Affiliations:** 1 Department of Plant Protection, College of Horticulture and Plant Protection, Yangzhou University, Yangzhou, 225009 Jiangsu Province People’s Republic of China; 2 Jiangsu Key Laboratory for Microbes and Functional Genomics, Jiangsu Engineering and Technology Research Center for Microbiology, College of Life Sciences, Nanjing Normal University, Nanjing, 210023 People’s Republic of China; 3 Joint International Research Laboratory of Agriculture and Agri-Product Safety of Ministry of Education of China, Yangzhou University, Wenhui East Road No.48, Yangzhou, 225009 Jiangsu Province People’s Republic of China

**Keywords:** *N*^*6*^-methyladenosine, Rice, Plant viruses, Interactions

## Abstract

**Background:**

*N*^*6*^-methyladenosine (m^6^A) is the most common RNA modification in eukaryotes and has been implicated as a novel epigenetic marker that is involved in various biological processes. The pattern and functional dissection of m^6^A in the regulation of several major human viral diseases have already been reported. However, the patterns and functions of m^6^A distribution in plant disease bursting remain largely unknown.

**Results:**

We analyse the high-quality m^6^A methylomes in rice plants infected with two devastating viruses. We find that the m^6^A methylation is mainly associated with genes that are not actively expressed in virus-infected rice plants. We also detect different m^6^A peak distributions on the same gene, which may contribute to different antiviral modes between rice stripe virus or rice black-stripe dwarf virus infection. Interestingly, we observe increased levels of m^6^A methylation in rice plant response to virus infection. Several antiviral pathway-related genes, such as RNA silencing-, resistance-, and fundamental antiviral phytohormone metabolic-related genes, are also m^6^A methylated. The level of m^6^A methylation is tightly associated with its relative expression levels.

**Conclusions:**

We revealed the dynamics of m^6^A modification during the interaction between rice and viruses, which may act as a main regulatory strategy in gene expression. Our investigations highlight the significance of m^6^A modifications in interactions between plant and viruses, especially in regulating the expression of genes involved in key pathways.

**Supplementary Information:**

The online version contains supplementary material available at 10.1186/s13059-021-02410-2.

## Background

*N*^*6*^-methyladenine (m^6^A) RNA methylation is one of the most common RNA modifications in prokaryotes and eukaryotes [1]. m^6^A methylation and its biological functions in prokaryotes and eukaryotes have been the focus of research, recently [2–6]. Modification of m^6^A methylation was first reported 40 years ago [7, 8]. The modification contributes to the generation, localisation, and functionality of messenger RNA (mRNA) through the regulation of stability and translation [2–5]. Our understanding of the essential roles of m^6^A in viruses, fungi, animals, and plants is increasing [9–11]. Most studies have focused on the influence of m^6^A on development, evolution, and physiology, especially in plants [12–16]. However, little is known about the precise functions of m^6^A in the interactions between plants and parasites, and whether these functions are involved in physiological and pathological changes.

Methylation of adenosine is catalysed by “WRITER”, a large molecular weight (> 1 MDa) RNA methyltransferase complex in plants. It is composed of two MTA-70 family proteins (MTA and MTB), FK506-binding protein 12 (FKBP12) interacting protein 37 (FIP37), VIRILIZER (VIR), and ubiquitin ligase HAKAI [17–19]. The m^6^A methylation is reversible and can be removed by “ERASER”, which includes two main components in *Arabidopsis*: ALKBH9B and ALKBH10B. Recognition of the m^6^A methylated genes involves “READER”, which recognises YTH domain-containing proteins, such as evolutionarily conserved C-terminal regions (ETC2), ETC3, and ETC4 [12, 20]. m^6^A methylation is performed by adding the methyl group to the *N*^*6*^ position of adenosine. S-adenosylmethionine (SAM) often serves as a methyl “DONOR” for almost all cellular methylation reactions [21, 22]. SAM generation from methionine and adenosine triphosphate (ATP) involves SAM synthetases [22]. The “WRITER”, “READER”, “ERASER”, and “DONOR” proteins are tightly associated with multiple biological processes in plants. Previous studies showed that loss-of-function mutants of “WRITER” genes (*FIP37* and *OsFIP*) resulted in serious developmental malformations, and even embryonic lethality [14, 17]. The “ERASER” genes, which include *AtALKBH9B* and *AtALKBH10B*, affect the infectivity of the alfalfa mosaic virus (AMV) to *Arabidopsis* because of the interaction of the viral capsid protein (CP) and the eraser protein; these genes also affect the floral transition of *Arabidopsis* due to the altered stability of mRNAs targeting key flowering time genes [20, 23]. “READER” genes are involved in leaf formation and trichome morphology. The binding of ECT2 to the RNA “UGUA” m^6^A motif could regulate the transcript stability of trichome morphogenesis-related genes [12, 24]. The collective findings concerning m^6^A biological functions strongly indicate that m^6^A methylation has important roles in the regulation of gene expression in plants, such as the regulation of the mRNA stability of target genes.

Although the precise molecular functions of m^6^A dynamics are not fully understood, several studies have revealed relationships with RNA stability [25], triggering RNA structure switches [26], translation [27], splicing [28], microRNA (miRNA) processing [29], and RNA export [30]. The recent development of the high-throughput methylated RNA immunoprecipitation sequencing (MeRIP-seq) technology enabled the identification of transcriptome-wide m^6^A modification. The findings demonstrated the enrichment of m^6^A around the start codon, stop codon, and 3′-untranslated regions (3′-UTRs) with an “RRACH” consensus motif in all eukaryotes that have been analysed [15, 31, 32]. These findings strongly suggest a conserved mechanism of m^6^A deposition in eukaryotic mRNA and stimulated many hypotheses about their roles.

The dynamics of m^6^A modifications include the exact sites, frequency of methylation, and percentage of methylated genes. These dynamics may vary in plants exposed to biotic and abiotic stress, especially under parasitic infection. m^6^A modification is important in the regulation of viral replication and the viral life cycle in animal systems [6, 11, 33–35]. However, in plants, most viruses are RNA viruses. m^6^A modification, as a widespread modification in plants, may have profound potential roles in modulating virus infection. In *Arabidopsis*, the genomic RNA accumulation of AMV was reportedly decreased in the T-DNA insertion mutant of *Atalkbh9b*, and the virus infectivity was impaired, whereas the infectivity of cucumber mosaic virus (CMV) was not altered [23]. This may be because AtALKBH9B can interact with the CP of AMV, but not with the CP of CMV [23]. Infection of tobacco plants with tobacco mosaic virus (TMV) significantly reduced the overall m^6^A modification levels [36]. In addition, a conserved domain-containing ALKB has been identified in the genomic RNA of several single-stranded plant RNA viruses [37, 38]. These results imply that the m^6^A modification may act as a fine modulation mechanism for plants responding to viral infection. Some plant viruses have evolved strategies to defend against the host m^6^A modulation system. However, the m^6^A dynamics in interactions between plant and virus remain unclear, as do the molecular functions of the modification and the relationship between the expression levels of host main disease resistance pathway-related genes and the m^6^A modification region on the gene bodies.

Presently, combined MeRIP-seq and transcriptome analyses revealed the activation of the overall m^6^A modification levels during plant virus infection. Further, the distribution of m^6^A peaks in both viral and rice genomes were mapped for the first time. The m^6^A modification was tightly associated with genes that were not actively expressed in rice infected with viruses. Gene ontology (GO) analyses showed that RNA binding activity apparently influenced the molecular functions. Moreover, the most common consensus was analysed in rice with and without viruses’ infection.

We also found m^6^A levels were associated with the expression of the key genes involved in jasmonate (JA)-mediated RNA silencing. Our findings also revealed the involvement of the m^6^A modification in the relative expression of the main antiviral pathway-related genes in plants, such as the genes of main m^6^A methylation machinery, RNA silencing, and phytohormone metabolism. These data provide evidence that m^6^A modification participate in and alter the physiological and pathological status of rice plants during interactions with viruses.

## Results

### Confirmation of infection with RSV and RBSDV in rice seedlings

**Fig. 1:**
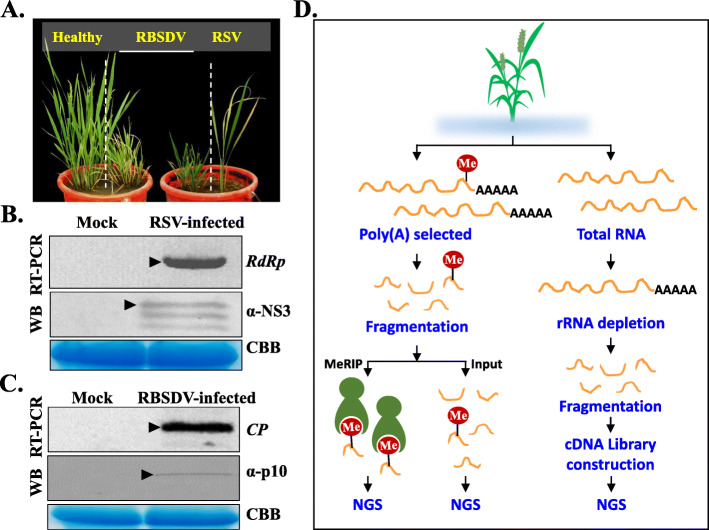
Flow chart of the investigation of m^6^A methylation during infection of rice by RBSDV or RSV. **A** Symptoms of RBSDV- and RSV-infected rice plants in plastic buckets at 60 days post infection (dpt). The left plant is a mock-treated plant, the middle two plants are infected with RBSDV, and the plant on the right is infected with RSV. **B** RT-PCR and western blot (WB) detection of RSV using a specific pair of primers corresponding to *RdRp* and anti-NS3 specific antiserum. Total proteins were stained with Coomassie brilliant blue (CBB), which was treated as the loading control. **C** RT-PCR and WB were performed to detect RBSDV in rice using a specific pair of primers corresponding to *CP*. Anti-p10 specific antiserum was carried out for the WB. **D** Experimental flow chart of m^6^A-IP-seq and RNA-seq using RBSDV- and RSV-infected plants. NGS, next-generation sequencing. MeRIP, methylated RNA immunoprecipitation

To confirm the infection of rice plants with the two plant viruses, disease development was observed. SBPH-infested rice plants harbouring RBSDV began to exhibit plant growth abnormalities, such as dwarfism and leaf darkening. At 20 days-post-transplantation (dpt), these plants showed more serious developmental abnormalities. At 60 dpt, SBPH-infested rice plants infected with RSV showed leaf yellowing, stripe, chlorosis, and slower plant growth (Fig. 1A). Leaves were collected from these symptomatic rice plants for RT-PCR and western blotting analyses to detect the target viruses (Fig. 1B, C). The specific pairs of primers corresponding to RSV and RBSDV are shown in Additional file 2: Table S1. RT-PCR showed a specific band with the expected size appeared in RSV- and RBSDV-infected rice plants compared with mock-treated plants, respectively. These results and those of western blots using RSV NS3 and RBSDV p10 antisera (Fig. 1B, C) confirmed the independent infection of the plants by the viruses following inoculation.

### Transcriptome-wide mapping of m^6^A in rice

**Fig. 2:**
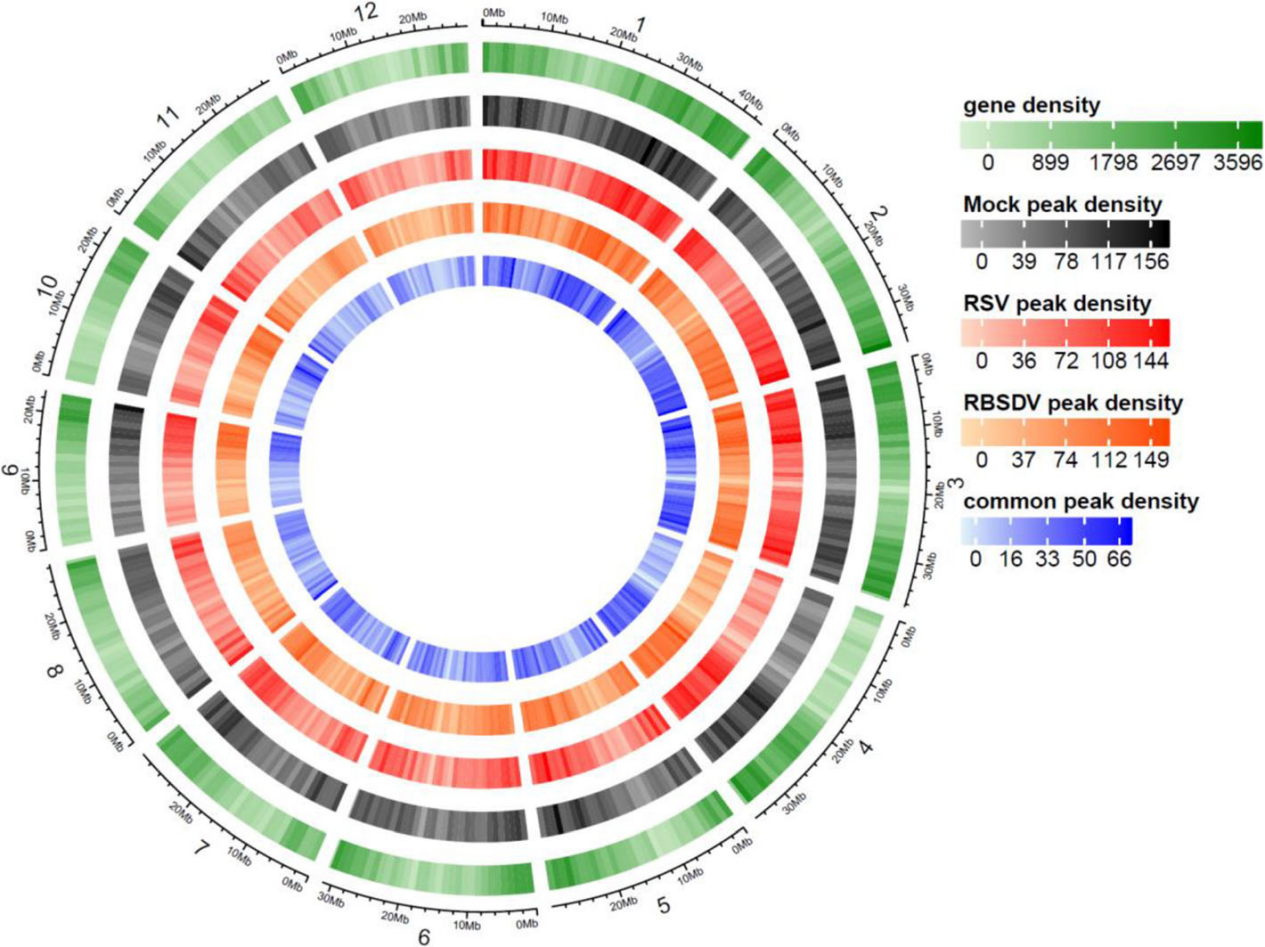
Circos plots of the m^6^A methylome in rice plants infected with RBSDV or RSV. The six rings from the outside to the inside show the genomic positions (1st), gene density (2nd), peak density of mock-treated rice plants (3rd), peak density of RSV-treated rice plants (4th), peak density of RBSDV-treated rice plants (5th), and the common peak density of RBSDV- and RSV-infected rice plants (6th)

To obtain a transcriptome m^6^A methylation modification map in rice, the mock-, RSV-, and RBSDV-infected rice samples were used for further analyses. The m^6^A analysis procedures were described, and the samples that were used for input (non-IP control), m^6^A-based RNA Immunoprecipitation (m^6^A-IP), and RNA-seq were clearly indicated (Fig. 1D). A series of IP, input, and mRNA libraries were constructed and sequenced, respectively (Additional file 2: Table S2). Samples of these series libraries came from mock-, RSV-, and RBSDV-infected rice leaves at 60 dpt. Each treatment was performed with two biological replicates. Raw sequencing data were further processed for adaptor and low-quality base removal. The obtained clear reads were aligned to the rice reference genome (*Oryza sativa.* IRGSP-1.0). Read distribution analysis showed that all the m^6^A-IP samples were highly enriched around the stop codon and within 3′-untranslated regions (3′-UTRs), which are in line with the previous reports in HIV-infected T cells and maize and suggested the m^6^A-IP sequencing data are reliable and with a high authenticity [6, 15] (Additional file 1: Fig. S1). The m^6^A-IP-seq analyses detected more than 26,000 m^6^A peaks in each individual treatment and biological replicate (Fig. 4A, Additional file 1: Fig. S2). For each treatment (one individual virus infection), high-confidence peaks were identified (Additional file 2: Table S3). Briefly, the regions that overlapped in at least one of the two replicates were designated high-confidence m^6^A peak regions. Confident peaks from different experimental conditions were further integrated into a unique m^6^A peak map. Consequently, a total of 26,390, 27,038, and 26,675 unique m^6^A peaks with high confidence (*p* < 0.05, fold change > 1.5) for mock-, RBSDV-, and RSV-infected samples were detected, respectively (Fig. 4A, Additional file 1: Fig. S2). After comparison with mock treatment, the RBSDV- and RSV-infected samples displayed 8011 and 6603 different regulated peaks, accounting for an average of approximately 1 m^6^A peak within transcription units from each gene. Among these differential m^6^A peaks, there are 3897 and 2900 new peaks appeared upon RBSDV and RSV infection, and 4113, and 3702 common peaks in RBSDV- and RSV-infected sample compared with mock-treated rice, and 1503 differential m^6^A peaks were both appeared in RBSDV- and RSV-infected sample (Additional file 2: Table S4). These results suggested that 48.7% and 43.9% differential m^6^A peaks newly appeared upon RBSDV- and RSV infection of rice, respectively, which also indicated that m^6^A methylation was tightly associated with viruses’ infection of the plant. At the genomic level, these unique m^6^A-methylated peaks for the three treatments were unevenly distributed across each rice chromosome. The common peak density was also mapped. The gene density according to the previously reported data is presented in Fig. 2, and the m^6^A peak distribution density was highly consistent with the corresponding gene density on the same chromosome position in the mock sample.

### Widespread m^6^A methylation of RSV and RBSDV genomic RNA

**Fig. 3:**
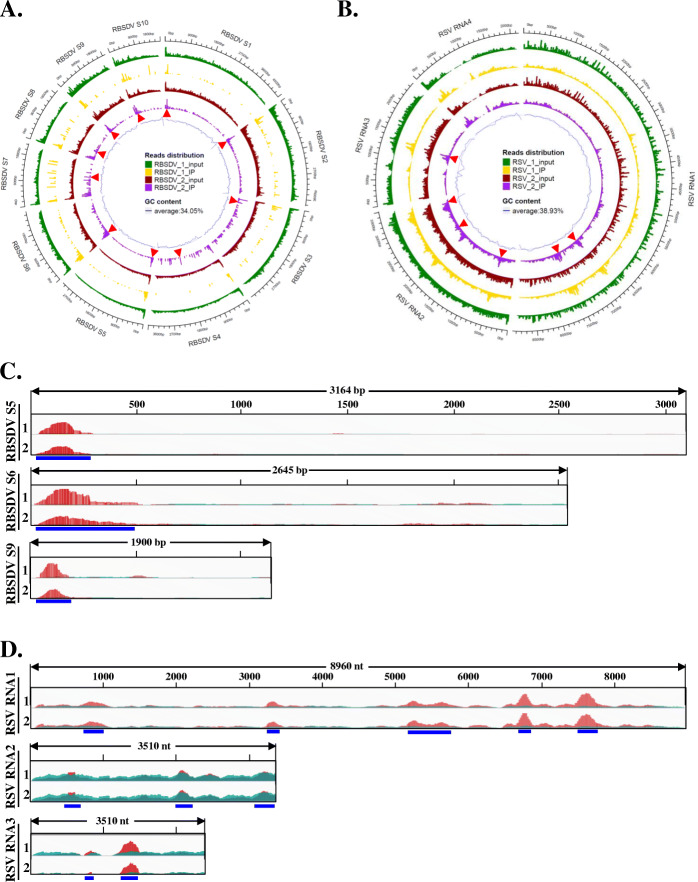
Circos plots of the m^6^A methylome in RBSDV and RSV genomic RNAs. **A** Distribution of m^6^A methylated reads on the ten RBSDV genomic RNAs. Six rings from outside to inside show genomic positions (1st), reads distribution of RBSDV_1_Input (2nd), reads distribution of RBSDV_1_IP (3rd), reads distribution of RBSDV_2_Input (4th), reads distribution of RBSDV_2_IP (5th), and the GC content of the genomic RNA (6th). **B** Distribution of m^6^A methylated reads on the four RSV genomic RNAs. Six outer rings were indicated similarly to RBSDV above. **C** m^6^A methylation peaks in the full-length RBSDV segment 5 (upper panel), 6 (middle panel), and 9 (bottom panel). The detail peaks regions and viral gene annotation are shown in the Additional file 2: Table S6. Top numbers show the full length of the analysed RNA segments, and bp is the short name of base-pair. Blue colour marked line shows the m^6^A peak region on viral genome, and number 1 and 2 mean the two replicate of the m^6^A-IP-sequencing. **D** Distribution of m^6^A peaks on the RSV genomic RNA1 (upper panel), RNA2 (middle panel), and RNA4 (bottom panel). The detail peak regions and viral gene annotation are shown in the Additional file 2: Table S6. Top numbers show the full-length of the analysed genomic RNAs, and the nt means nucleotide. Other marks are similar with Fig. 3C

The m^6^A-IP experiment was performed twice. The peak calling method used was stringent (false discovery rate < 0.01). The two replications of the next generation sequencing (NGS) data revealed high correlations with the bound RNAs (0.990), which indicated the high replicability of the sequencing results. The clear reads obtained by NGS were also aligned to the reference RBSDV and RSV genomic RNAs, and the m^6^A peaks spanning the full sequences of different segments of viruses were mapped (Fig. 3, Additional file 2: Table S5). In particular, clusters of m^6^A peaks were clearly observed in the 5′ terminal of RBSDV genomic S1, S2, S3, S4, S5, S6, S9, and S10, and some discrete peaks appeared in S4, and S7 (Fig. 3A, red arrows). In RSV-infected sample, the main m^6^A peak clusters were located on the genomic RNA2, RNA3, and RNA4, and compared to the input, several clearly m^6^A peaks were in the RNA1 to RNA4, two m^6^A peaks located to the 3′ terminal of RNA1 (Fig. 3B, red arrows). We also exhibited the fine m^6^A peaks that distributed to the each viral genomic RNAs (Additional file 2: Table S5), and the viral-specific m^6^A peaks that distributed on RBSDV S5, S6, and S9 (Fig. 3C) and RSV RNA1, RNA2, and RNA3 (Fig. 3D) were selected and exhibited. Our results suggested that the m^6^A modification often occurred in the 5′-terminal of the genomic RNAs of RBSDV, while they were random distributed on the RSV genome. These maybe resulted from the characteristic of the two completely different plant viruses (RSV is a single-strand RNA virus, while RBSDV is a double-stranded RNA virus). Taken together, in addition to the mRNA of the host plant, viral mRNAs could also be *N*^*6*^-methyladenosine methylated under interactions between virus and rice, and the m^6^A distribution pattern on viral genomic RNA was specific and novel.

### Activation of rice m^6^A RNA methylation levels upon virus infection

**Fig. 4:**
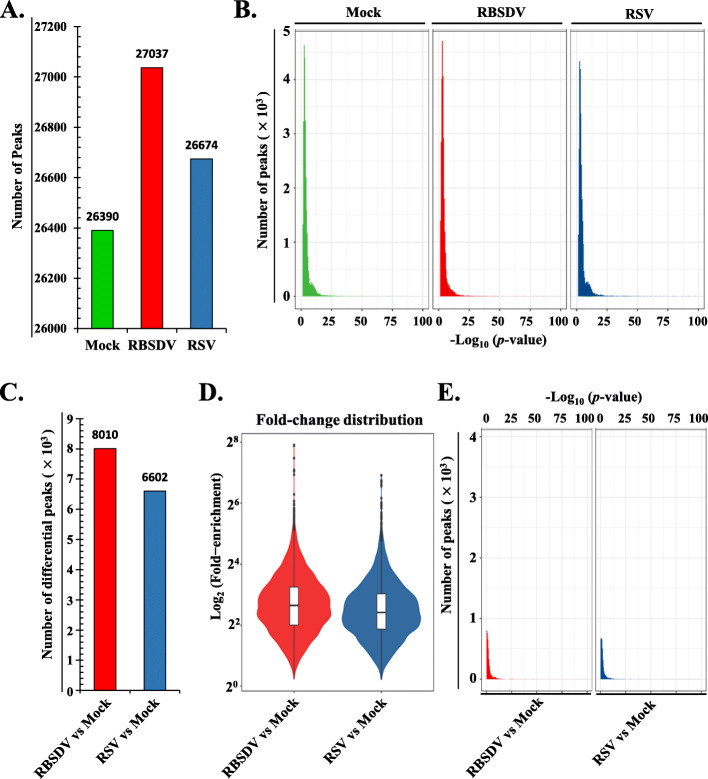
Activation of rice m^6^A methylation by virus infection. **A** Histograms show the number of unique peak in mock-treated and RBSDV- and RSV-infected rice plants. The Y-axis represents the peak number, and the X-axis represents the treatments. **B** Significant distribution analysis of the different peaks in mock-treated and RBSDV- and RSV-infected rice samples. The Y-axis represents the peak number, and the X-axis represents the negative value of the logarithm of the *p-* value base-10. **C** Histogram showing the different regulated numbers of m^6^A methylated genes in RBSDV- and RSV-infected rice compared with mock-treated rice samples. **D** Comparisons of the fold change of the different regulated peaks in RBSDV- and RSV-infected rice plants. The Y-axis represents the logarithmic of peak folded-enrichment base-2. **E** Significant distribution analysis of the different peaks in RBSDV- and RSV-infected rice samples. The Y-axis represents the numbers of different regulated peaks, and the X-axis represents the negative value of the logarithmic value of the *p*- value base-10

To explore the changes of m^6^A RNA methylation levels in virus-infected rice, the sequencing data were analysed. Collectively, there were 15,977, 16,854, and 16,267 m^6^A methylated genes that corresponding to mock-, RBSDV-, and RSV-infected rice, respectively (Additional file 2: Table S6). The findings clearly indicated that rice m^6^A RNA methylation was enriched under RSV and RBSDV infection. In terms of differential m^6^A peak number, the enriched peaks were also increased in rice infected with viruses (Fig. 4A, Additional file 2: Table S6). Meanwhile, the confidence of the peak significances was determined by calculating the correlations of the peak numbers and the *p* value (Fig. 4B). The horizontal ordinate dimension (-log_10_[*q*-value]) of most of the m^6^A peaks ranged from 4 to 10. The findings indicated that most of the peaks were highly confident and credible. The differential m^6^A peaks were selected and compared to those obtained for mock-treated rice plants. There were 8,010 and 6,602 unique and different m^6^A peaks for RBSDV- and RSV-infected rice plants compared to the mock-treated sample, respectively (Fig. 4C). For the digital exhibition of the m^6^A signal intensity, a violin plot was used to show the fold enrichment distribution (Fig. 4D). The median number of the peak enrichment-fold was approximately 40 and 76 (Additional file 2: Table S7). To explore the confidence of the different peaks deposited in RBSDV- and RSV-infected samples, the significance distribution of different m^6^A peaks was analysed by correlation with the *p-* value of each peak and peak number (Fig. 4E). The main differential regulated m^6^A peaks were deposited in 3 on the horizontal axis. The finding indicated that the different m^6^A peaks of rice mRNA under viral infection was confident and reliable. Taken together, the m^6^A modification levels of rice mRNAs were enriched under infection by plant viruses.

### Association of m^6^A with genes that were not actively expressed in virus-infected rice plants

**Fig. 5:**
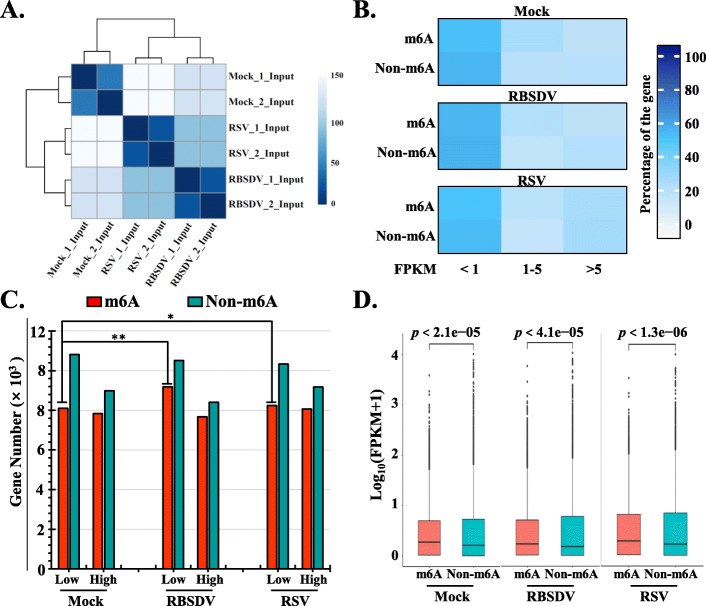
Analyses of the relationship between m^6^A methylation with expression levels of the target gene in rice under plant virus infection. **A** Euclidian distance (ED) coefficients among gene expression profiles generated by RNA-seq analysis of the two biological replicates of the three treatments. RNA-seq was performed simultaneously with m^6^A-IP-seq with total RNA extracted at 60 dpt. A lower value means a closer ED of the two compared objects, and with higher reproducibility of the two replicates. **B** The percentage of rice m^6^A methylated and un-methylated genes at a defined FPKM levels (< 1, 1–5, and > 5). Different colour densities indicate different percentages of the corresponding gene. **C** Comparisons of number of non-m^6^A methylated genes and number of m^6^A methylated genes in their gene bodies with high (FPKM > 1) and low (FPKM > 1) expression levels in the three treatments. Relationships between gene expression and number were tested using the chi-square test. “*” indicate *p-* value < 0.05, and “**” means *p-* value < 0.01. **D** Box plot comparing FPKM expression levels between non-m^6^A methylated genes and m^6^A methylated genes in the three treatments. A two-tailed unpaired Student’s *t* -test was performed to calculate the *p-* values of these three treatments

RNA deep sequencing (input samples) of the 60 dpt rice plants of mock-, RBSDV-, and RSV-infected samples with two biological replicates were performed, and each replicate was used to examine the correlation between m^6^A modification and gene expression in rice. The euclidian distance (ED) of the two replicates’ reads was small, and the square colour of the two was close (Fig. 5A). The findings suggested that the experiments were reproducible between the two replicates. Based on the confident sequencing results, the m^6^A peaks of each treatment were annotated to the genes. Rice genes that could be annotated by the m^6^A peaks were called m^6^A gene, and that couldn’t be annotated were called non-m^6^A gene in the next analyses. According to the Fragments Per Kilobase of exon model per Million mapped fragments (FPKM) (Additional file 2: Table S6), these genes were divided into three groups (FPKM < 1, 1 < FPKM < 5, and FPKM > 5), and the ratio of gene number of each category were calculated and showed by the heatmap (Fig. 5B). Either in the mock-treated sample or the viruses’ infected rice, most of the analysed genes (m^6^A or non-m^6^A genes) were mainly distributed in the low expressed category (FRKM < 1) (Fig. 5B). Compared to the mock-treated sample, the ratio of m^6^A methylated genes were increased in low expressed category of RSV- and RBSDV-infected samples (Fig. 5B). When these genes (m^6^A and non-m^6^A) were divided into highly expressed genes (FRKM ≥ 1) and genes that were not highly expressed (FRKM < 1), the expression of genes that was high or low were showed in mock-, RBSDV-, and RSV-infected samples (Fig. 5C), and we found that most genes were non-methylated and distributed in low expression category in mock-, RBSDV-, and RSV-infected rice samples (Fig. 5C). The m^6^A methylated genes were slightly enriched in the low expression category upon viruses’ infection of rice compared to the mock-treated sample (Fig. 5C). Either in mock-treated or in RSV-infected and RBSDV-infected samples, the m^6^A-methylated genes were faintly expressed at a higher level than those of non-m^6^A methylated genes (Fig. 5D), and the highly expressed genes displayed a lower m^6^A occupancy (Fig. 5B). Hence, the m^6^A methylation mainly occurred in low expressed genes either in mock-treated or in RSV- and RBSDV-infected rice samples and the m^6^A modified gene number were slightly enriched in the low expressed category upon viruses’ infection.

### Different regulated m^6^A-methylated genes in virus-infected rice plants

**Fig. 6:**
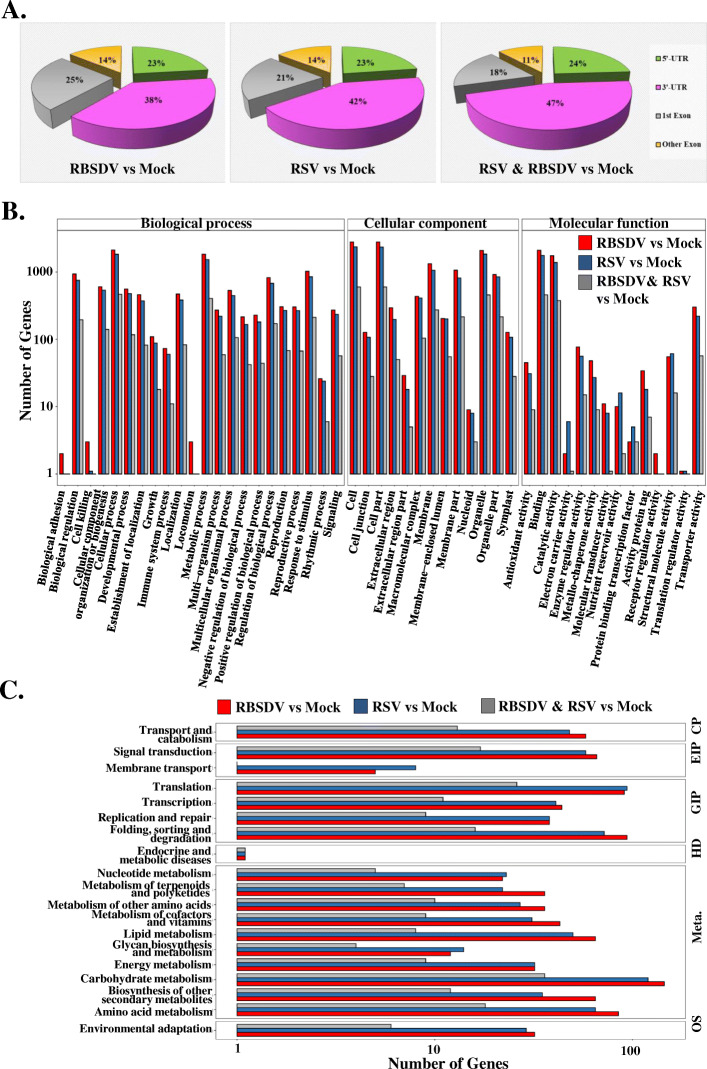
GO and KEGG analyses of different m^6^A methylated genes in RBSDV- and RSV-infected rice plants. **A** Percentages of the different gene bodies (5′-UTR, 3′-UTR, 1st exon, and other exons) in different m^6^A methylated genes in rice infected with RBSDV alone, RSV alone, or with both. **B** GO analysis of the different m^6^A methylated genes in rice infected with RBSDV alone, RSV alone, or with both compared with mock-treated rice plant. **C** KEGG analysis of the different m^6^A methylated genes in rice infected with RBSDV alone, RSV alone, or with both

To investigate which pathway-related genes were m^6^A methylated, the obtained different m^6^A peaks were mapped to the rice reference genome. The gene was divided into 5′-UTR, 3′-UTR, first exon region, and exon excluding the 5′-UTR and 3′-UTR in the protein-coding region. The sequenced clear m^6^A data were aligned to these functional elements. We found that 38%, 42%, and 47% of the m^6^A modification sites were located on the 3′-UTR of the genes in RBSDV-infected, RSV-infected, and common RBSDV and RSV peaks, respectively (Fig. 6A). Furthermore, 23%, 23%, and 24% of the m^6^A modification sites were deposited on the 5′-UTR regions of the aforementioned genes (Fig. 6A). These results indicated that most of the m^6^A sites were located in the non-coding region of genes. Gene ontology (GO) analyses were performed to provide insight into the biological functions of different m^6^A modifications in rice. The methylated genes were involved in multiple molecular functions, particularly in binding and catalytic activity (Fig. 6B). Thus, m^6^A modifications may act as epigenetic markers that mediate molecular interactions between rice and plant viruses. Furthermore, Kyoto Encyclopedia of Genes and Genomes (KEGG) analyses of the different m^6^A modification-related genes were performed. The five enriched pathways were carbohydrate metabolism, translation, protein folding/sorting/degradation, amino acid metabolism, and signal transduction (Fig. 6C). these results suggested that m^6^A modification was widely involved in and closely related to the intracellular carbohydrate metabolism, amino acid metabolism, and protein translation/folding/sorting/degradation in RSV- and RBSDV- infected rice sample.

### Analysis of consensus motifs related to m^6^A modifications

**Fig. 7:**
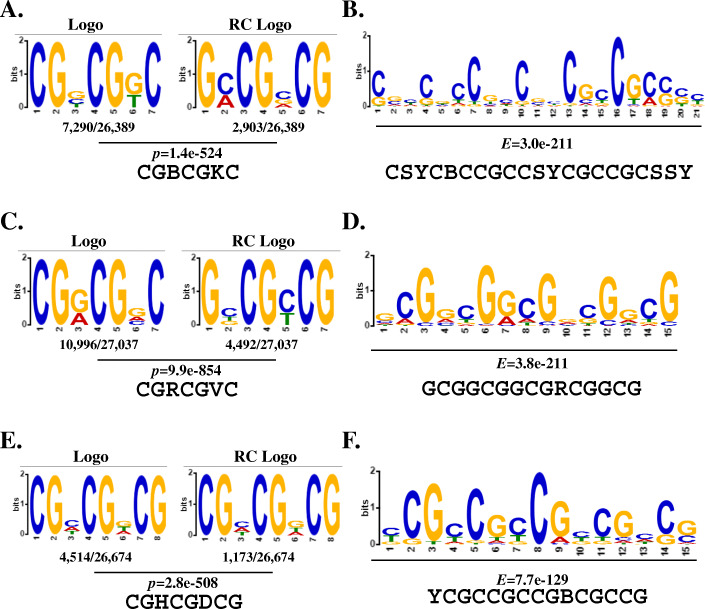
Identification of predominant consensus motifs containing m^6^A methylation sites in mock-treated and RBSDV- and RSV-infected rice plant using DREME and MEME suites. **A** Sequences logo representations of the consensus motifs containing m^6^A sites in mock-treated rice samples. **B** The most enriched consensus motif in RBSDV-infected samples. **C** The most enriched consensus motif in RSV-infected samples

To investigate whether there are conserved motifs in clear sequencing reads of mock-, RBSDV-, and RSV-infected rice samples, the MEME suite software was used for *de novo* scanning of the enriched motifs. In total, 172,801, 152,136, and 168,666 raw m^6^A peaks appeared in the sequencing data corresponding to the mock-, RBSDV-, and RSV-infected rice samples (Additional file 2: Table S8). When the parameter was set as the fold enrichment (FC) > 1.5 (*p* < 0.05), 26,389, 27,038, and 26,675 significantly enriched m^6^A peaks were identified in mock-, RBSDV-, and RSV-infected rice samples, respectively. These significantly enriched peaks were performed to do *de novo* searching of the common consensus by software. The “CGBCGKC” (B = C, G, or T; K = G or T), “CGRCGVC” (R = A or G; V = A, G or C), and “CGHCGDCG” (H = A, C or T; D = A, G or T) motifs were enriched in mock-, RBSDV-, and RSV-infected rice m^6^A methylated reads, respectively, using the DREME suite (Fig. 7A, C, and E). MEME suite scanning revealed the enrichment of the longer conserved motifs “CSYCBCCGCCSYCGCCGCSSY”, “GCGGCGGCGRCGGCG”, and “YCGCCGCCGBCGCCG” in m^6^A methylated reads of mock-, RBSDV-, and RSV-infected rice (Fig. 7B, D, and F). Besides, the enriched top five consensus in mock-, RBSDV-, and RSV-infected rice were selected and exhibited (Additional file 2: Table S9), and results showed the dynamic of the ranking with viruses’ infection. The top four widely studied motifs in other species were selected and analyses using the DREME and MEME suites in the mock-, RBSDV-, and RSV-infected rice samples (Additional file 2: Table S10), and the results showed that the most common motifs still were “RRACH” (68%), and “URUAY” (28%). Taken together, these results implied that the common recognition mechanism of the m^6^A methylated sites during the plant-virus interaction, and viruses’ infection caused the dynamics of the top five consensus ranking.

### Rice m^6^A methylation modifications display sensitive and dynamic responses to infection of rice plants by viruses

**Fig. 8:**
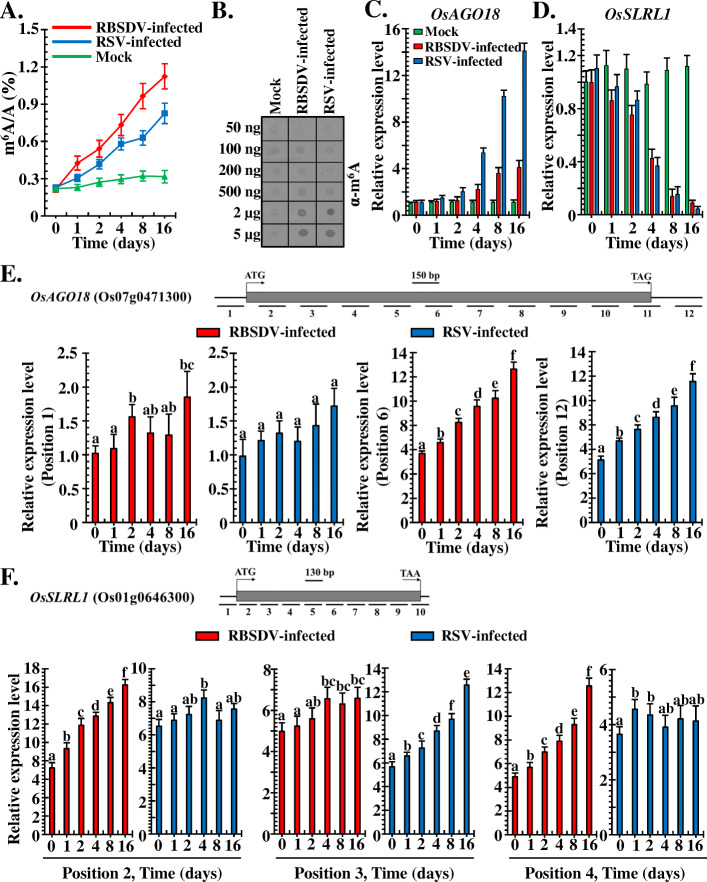
Rice m^6^A methylation levels are positively associated with the expression of key genes involved in antiviral RNA silencing pathways and plant hormone signals. **A** Comparisons of m^6^A methylation levels of the respect mock-treated and RBSDV- and RSV-infected rice plants at 0, 1, 2, 4, 8, and 16 dpi by LC-MS/MS. Error bars indicate mean ± SD, with three biological replicates. **B** Dot-blot analysis of m^6^A levels in extracted total RNA from samples at 16 dpi using the specific anti-m^6^A antibodies. The left side of the membrane depicts the amount of loaded mRNA from mock-treated and RBSDV- and RSV-infected rice plants, respectively. **C** qRT-PCR analysis of the relative expression of *OsAGO18* in mock-treated and RBSDV- and RSV-infected rice plants at 0, 1, 2, 4, 8, and 16 dpi. **D** Relative expression of the *OsSLRL1* in the three treatments at 0, 1, 2, 4, 8, and 16 dpi. **E** Analysis of m^6^A methylation levels on different fragments of *OsAGO18* by m^6^A-IP-qPCR. The upper panel indicates the gene structures of *OsAGO18* labelled with fragments amplified in the m^6^A-IP-qPCR assay. The results of positions 1 and 12 were chosen for figure exhibition. **F** m^6^A-IP-qPCR assay of the m^6^A methylation levels of different fragments on *OsSLRL1*. Similarly, the upper panel represents the gene structures labelled with fragments amplified in the m^6^A-IP-qPCR analyses. Results of positions 2, 3, and 4 were selected for the display in the figure. Error bars denote mean ± SD, n = 3 biological replicates in all qRT-PCR assays

Abiotic and biotic stress, such as heat, cold, salt, and drought, and a variety of fungal, bacterial, viral, and nematode plant pathogens often affect the growth and development of rice plants, which poses a significant threat to the yield and restricts the global distribution of rice plants. In addition to the visual phenotype changes to these stresses, there are sophisticated and fine gene expression and regulation networks behind. Hence, we wondered whether mRNA m^6^A modification was involved in the interactions between rice and plant viruses. LC-MS/MS was used to track the m^6^A methylation dynamics of rice mRNA in plants infected with RBSDV or RSV. The difference in the potential epigenetic basis of the virus infection on rice plants was also investigated. Compared to the mock treatment, the *N*^*6*^-methyladenosine level was significantly increased in RBSDV- and RSV-infected rice compared to the mock-treated sample just at the one-day-post-infection (dpi), and the FC of the m^6^A content was further increased with the infection time extended from 2 days to 16 days (Fig. 8A). These results demonstrated that the m^6^A methylation was sensitive and dynamic upon viruses’ infection. The FC of the m^6^A content in the RBSDV-infected samples was approximately 5 times higher than that in the mock-treated sample at 16 days, and 3 times higher in the RSV-infected sample (Fig. 8A). Total RNAs were extracted, then the mRNAs were isolated. Nucleic acid-based dot-blot analyses were performed. The blotting results showed that with the increase in mRNA loading, the colour reaction of virus-infected rice plants became stronger compared with the mock-treated samples (Fig. 8B). These findings indicated that the infection of plants with viruses markedly increased the *N*^*6*^-methyladenosine level of plant mRNAs, and the *N*^*6*^-methyladenosine level was tightly correlated with virus infection of rice plants.

### Correlation of m^6^A levels with expression of key genes involved in plant antiviral RNA silencing pathway and hormone metabolism

The m^6^A methylation level of rice total mRNAs was strongly correlated with virus infection of the plants (Fig. 8B). This prompted us to explore the potential molecular mechanisms. We focused on the virus infection of rice and explored whether m^6^A modifications were involved in the regulation of the key genes’ expression that related to virus infection. Based on the m^6^A-IP sequencing results (Additional file 2: Table S11), the m^6^A methylated candidate genes, *ARGONAUTE 18* (*OsAGO18*), and *SLENDER RICE LIKE 1* (*OsSLRL1*), were selected. OsAGO18 was reported to participate in anti-virus RNA silencing pathways in rice by binding small interfering RNAs (siRNAs) [39]. The expression of OsAGO18 can be activated by jasmonate (JA) signalling [40]. OsSLRL1 is a DELLA family protein that regulates plant hormone metabolism and mediates the growth and development of plants [41–43]. qRT-PCR was performed and the relative expression levels of the selected candidate genes were determined in plants infected by the virus from 0 to 16 days. *OsAGO18* was upregulated by 14-fold and 4-fold in RBSDV- and RSV-infected rice plants, respectively, compared with mock-treated plants, while the *OsSLRL1* was respectively downregulated by 12-fold and 25-fold in RBSDV- and RSV-infected rice plants, respectively (Fig. 8C, D). Based on these results, we concluded that the antiviral RNA silencing pathway was activated, and the synthesis and degradation of plant hormones controlling the growth, development, and basal resistance to pathogens were regulated in virus-infected rice plants.

To validate whether the m^6^A modifications were correlated with the expression of key genes, m^6^A methylation-based RNA immunoprecipitation and qRT-PCR technology (m^6^A-IP-qPCR) was performed. The m^6^A levels in position 12 of *OsAGO18*, not position 1 or other regions, was increased by approximately 13-fold and 11-fold in RBSDV- and RSV-infected samples, respectively, compared with mock-infected rice plants (Fig. 8E). The m^6^A levels in positions 2 and 4 of *OsSLRL1* were increased by 16-fold and 12-fold in RBSDV-infected rice plants, respectively, but not in position 3 (approximately 6-fold and not responsive to virus infection) (Fig. 8F). For RSV-infected samples, m^6^A levels in positions 2 and 4 of *OsSLRL1* were not significantly changed (approximately 6- and 4-fold, respectively) under virus infection, whereas the m^6^A level was markedly up-regulated at position 3 (Fig. 8F). The collective findings indicated that changes in the relative expression of *OsAGO18* and *OsSLRL1* tightly correlated with the changes in their m^6^A modification level and that the m^6^A methylation of different gene regions may have different effects on its expression. These findings could contribute to a deeper understanding of the molecular basis of interactions between rice and viruses.

### Involvement of rice m^6^A modification in the regulation of the expression of the main m^6^A modification machinery components in virus-infected plants

RNA m^6^A modifications occur in many eukaryotes, and it was done by the m^6^A methylation machinery. The WRITER, READER, ERASER, and methyl synthetases that produce the DONOR (methyl) are the four main components of the m^6^A methylation machinery. To investigate whether the gene expression of the main components of m^6^A methylation machinery can be affected by m^6^A modifications, qRT-PCR was performed. The relative gene expression levels of five WRITER genes (*OsMAT1*, *OsMAT2*, *OsMAT3*, *OsMAT4*, and *OsFIP*), five ERASER genes (*OsALKBH10B*, *OsALKBH9B-1*, Os05g0401500, *OsALKBH9B-2*, and *Os03g0238800*), 12 READER genes (*OsYTH1–12*), and five S-adenosyl-I-methionine synthetases (*OsSAM1*, *OsSAM1L*, *OsSAM2*, *OsSAM2L*, and *OsSAM3*) were determined in mock and virus-infected rice samples. The relationship between the relative expression level of target genes and m^6^A modification sites was counted by combination analyses (Additional file 2: Table S12), and no any fixed regular pattern has been found.

**Fig. 9:**
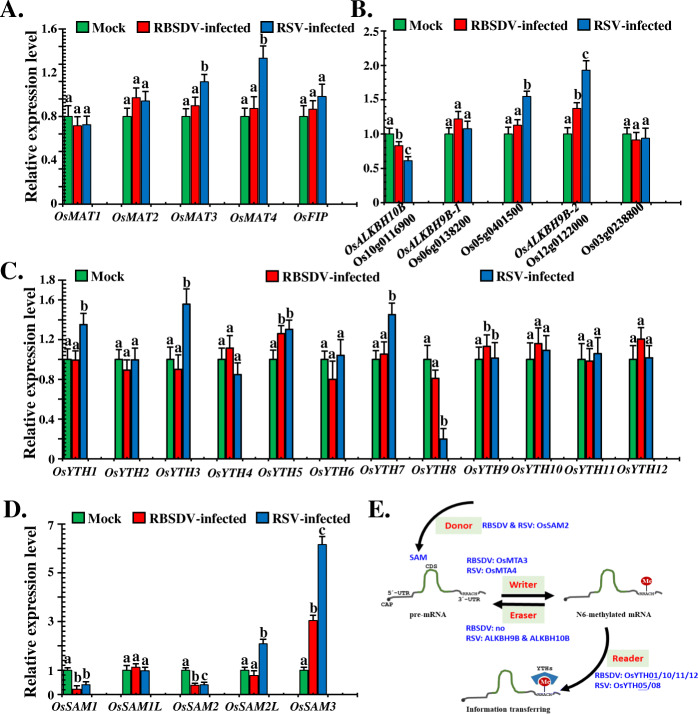
Integrated analyses of the main components of m^6^A methylation machinery in rice with m^6^A methylation modifications and gene expression in plants infected with viruses. **A** Relative expression levels of five “WRITER” components in rice plants by qRT-PCR analyses. **B** qRT-PCR analysis of the relative expression of five “ERASER” components in rice plants. **C** Relative expression levels of twelve “READER” component genes were determined by qRT-PCR. **D** Relative expression levels of five methyl “DONER” synthesis genes were analysed by qRT-PCR. **E** Rice m^6^A methylation pathways and related m^6^A methylated genes under plant virus infections. Blue coloured letters indicate the m^6^A methylated genes of certain treatments. For instance, RBSDV: OsMTA3, means the *OsMTA3* gene was methylated in RBSDV-infected sample. All qRT-PCR assays were performed with three biological replicates, and the error bars denote the mean ± SD

Compared with mock-treated samples, the *OsMAT3* and *OsMAT4* WRITER genes were significantly increased in RSV-infected samples, whereas they did not change in RBSDV-infected samples (Fig. 9A). For ERASER genes, the expression of *OsALKBH10* was significantly suppressed in both the RBSDV- and RSV-infected samples, both *Os05g0401500* and *OsALKBH9B-2* were up-regulated in RSV-infected samples, whereas the expression of *OsALKBH9B-2* was increased in RRSDV-infected samples (Fig. 9B). For READER genes, the expression levels of *OsYTH1*, *OsYTH3*, *OsYTH5*, and *OsYTH7* were up-regulated in RSV-infected samples, whereas *OsYTH8* was significantly decreased. Only the expression level of *OsYTH6* was increased in RBSDV-infected samples (Fig. 9C). For the methyl donor producer, the *OsSAM1* and *OsSAM2* expression levels were significantly down-regulated in both RBSDV- and RSV-infected samples, whereas the expression of *OsSAM2L* and *OsSAM3* in RSV-infected samples were up-regulated, and the expression of *OsSAM3* was also markedly increased in RBSDV-infected samples (Fig. 9D). Based on the relative expression results, we integrated the m^6^A-IP sequencing data with the m^6^A methylation machinery in rice (Additional file 2: Table S12). Modification of m^6^A occurs in genes of m^6^A methylation machinery under plant viruses’ infection. For the donor producer, *OsSAM2* was m^6^A methylated in both RBSDV- and RSV-infected samples. For the WRITER genes, *OsMTA3* and *OsMTA4* were m^6^A methylated in RBSDV- and RSV-infected samples, respectively. For ERASER genes, the m^6^A modification occurred on *OsALKBH10B* and *OsALKBH9B* in RSV-infected samples, whereas no ERASER genes were m^6^A methylated in RBSDV-infected samples. For READER genes, *OsYTH01*, *OsYTH10*, *OsYTH11*, and *OsYTH12* in RBSDV-infected samples, and *OsYTH05* and *OsYTH08* in RSV-infected samples were m^6^A methylated (Fig. 9E). In summary, the m^6^A modifications occurred in the genes that encoding the plant m^6^A methylation machinery, and probably regulated the dynamics of the target gene expression, which may act as a main post-translational gene expression regulation strategy under plant virus infection.

### Involvement of rice m^6^A modification in regulating the expression of the main antiviral RNA silencing components in virus-infected rice plants

To investigate whether m^6^A methylation modification was involved in the regulation of the main host antiviral RNA silencing pathways genes and the reported resistance genes corresponding to these two viruses. Genes, which included nine *DCLs*, five *RNA-dependent RNA polymerases* (*RDR*), 17 *ARGONAUTE* genes (*OsAGOs*), seven RBSDV resistance genes (*OsGDI homologous*), and two RSV resistance genes (*OsSOT1* and *OsStvb-i*), were selected for relative expression analyses. Integrated analyses of the antiviral genes with m^6^A modification sites on the gene body and relative expression level were determined in virus-infected rice plants.

**Fig. 10:**
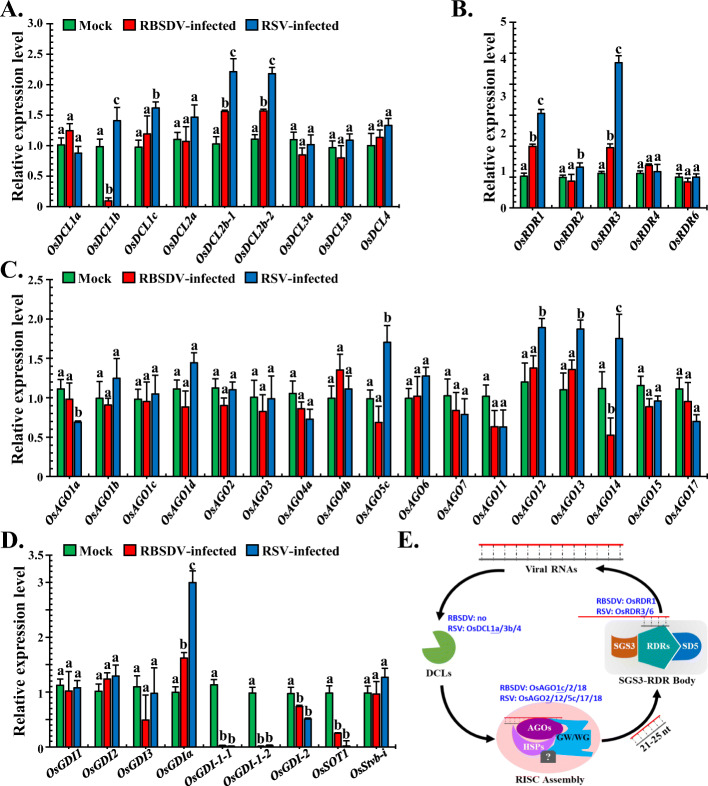
Integrated analyses of main antiviral RNA silencing pathway-related genes with m^6^A methylation modifications and gene expression levels in rice infected with viruses. **A** Relative expression levels of nine *OsDCL* genes in mock-treated and RBSDV- and RSV-infected samples. **B** Relative expression levels of five *OsRDR* genes in mock-treated and RBSDV- and RSV-infected samples using qRT-PCR analyses. **C** Relative expression levels of 17 *OsAGO* genes were determined with respect to mock-treated and RBSDV- and RSV-infected samples using qRT-PCR analyses. **D** Relative expression levels of nine resistance genes, including seven *OsGDI* genes, one *OsSOT1* gene, and one *OsStvb-i* were determined in mock-treated and RBSDV- and RSV-infected samples using qRT-PCR analyses. **E** Rice antiviral RNA silencing pathways and the related m^6^A methylated genes in virus-infected plants. Blue coloured letters depict the m^6^A methylated genes of a certain treatment, as detailed in Fig. 9E. All qRT-PCR assays were performed with three biological replicates. The error bars denote the mean ± SD

qRT-PCR revealed that both the *OsDCL2b-1* and *OsDCL2b-2* were significantly up-regulated in RBSDV- and RSV-infected samples than in mock-treated samples (Fig. 10A). In addition, the expression of *OsDCL1b* was greatly suppressed in RBSDV-infected samples, whereas it was increased in RSV-infected samples (Fig. 10A). Compared to the mock-treated samples, the expression of *OsRDR1* and *OsRDR3* in both RBSDV- and RSV-infected samples was markedly increased, and *OsRDR2* was upregulated only in RSV-infected samples (Fig. 10B). In RBSDV-infected samples, only the expression of *OsAGO18* was up-regulated significantly (Fig. 8C), whereas in the RSV-infected samples, *OsAGO5c*, *OsAGO12*, *OsAGO13*, *OsAGO14,* and *OsAGO18* were markedly increased (Figs. 8C and 10C). Concerning resistance genes, seven *ZmRabGDI* (Rab GTPase dissociation inhibiter) homologous genes in rice, which were defined as RBSDV resistance genes on maize [44], were screened out. The RSV resistance genes *OsSOT1* (Sulfotransferase 1) [45] and *OsStvb-i* (Stripe disease resistance i) [46], were also chosen. A total of nine genes were selected for further gene expression analyses. *OsGDI-1-1*, *OsGDI-1-2*, *OsGDI-2*, and *OsSOT1* were significantly decreased both in RBSDV- and RSV-infected rice plants compared to that in the mock-treated sample (Fig. 10D). In contrast, *OsGDIα* was markedly upregulated in RBSDV- and RSV-infected rice plants (Fig. 10D). The collective findings indicated that *OsDCL2b-1*, *OsDCL2b-2*, *OsRDR1*, *OsRDR3*, *OsAGO12*, *OsAGO13*, and *OsAGO18* were the main antiviral genes in the innate immune RNA silencing pathways, and that *OsGDIα*, *OsGDI-1-1*, *OsGDI-1-2*, *OsGDI-2*, and *OsSOT1* may act as resistance genes for both RBSDV and RSV.

To analyse the potential roles of m^6^A methylation in the expression of 57 selected genes in RNA silencing pathways and viral resistance genes, we systematically analysed their m^6^A modification status according to the results of m^6^A-IP-seq (Additional file 2: Table S13). For the main RNA silencing pathway genes, *OsAGO1c*, *OsAGO2*, *OsAGO18*, and *OsRDR1* were m^6^A methylated in RBSDV-infected rice. In RSV-infected samples, *OsDCL1a*, *OsDCL3b*, *OsDCL4*, *OsAGO2*, *OsAGO12*, *OsAGO5c*, *OsAGO17*, *OsAGO18*, *OsRDR1*, and *OsRDR3* were significantly methylated (Fig. 10E). In contrast, no viral resistance genes were m^6^A methylated (Fig. 10E). These results indicated the possible involvement of m^6^A methylation in the main antiviral RNA silencing pathways. The methylation may act as a fine regulator to mediate the spatial and temporal expression of target genes in arm race of plant-virus interaction.

### Integrated analyses of relative expression profile and m^6^A modification of seven phytohormone metabolism-related genes

Viruses have various strategies to reprogram the host’s cellular status to one that is prone to viral replication and spread. In plants, the specific environment created by viruses usually refers to phytohormone regulations of nearly all aspects of plant physiology, including development, growth, defence, and reproduction [47]. These phytohormones, which include jasmine acid (JA), salicylic acid (SA), abscisic acid (ABA), auxin, cytokinin (CTK), ethylene (ET), and brassinosteroids (BR), have significant roles in plant development and physiological regulations and are also involved in defence against pathogens [48, 49]. Previous findings suggest that phytohormones are strongly associated with virus infection and symptom development. We investigated whether these phytohormones are regulated by m^6^A modification and whether the expression profiles of their metabolic genes are altered by virus infection of rice plants. qRT-PCR was performed to determine the relative expression of the seven main phytohormone metabolism-related genes (Additional file 1: Fig. S3–S9). Further, integrated analyses of their relative expression and m^6^A modification sites were performed (Additional file 1: Fig. S10, Additional file 2: Table S14).

For the JA pathway, the biosynthesis genes *OsLOX8* and *OsLOX9* were markedly up-regulated in both RSV- and RBSDV-infected plants. *OsJMT1-1*, *OsAOS2*, and *OsLOX2* were markedly decreased, whereas *OsAOS1* and *OsLOX5* were not changed (Additional file 1: Fig. S3A). In addition, *OsJMT1*, *OsLOX1*, and *OsHPL3* were decreased in RSV-infected samples, whereas *OsJMT1* was increased in RBSDV-infected plants (Additional file 1: Fig. S3A). The expression profiles of 21 JA responsive genes were further investigated. *OsPR1a*, *OsWRKY28*, *OsPR2*, and *OsPR5-4* were significantly increased in both RSV- and RBSDV-infected plants, *OsPR5-3*, *OsMYB2*, *OsMYB55/56-1*, *OsWRKY10*, *OsRbohA*, *OsRbohB*, and *OsRbohC* were markedly decreased, and *OsPR1b*, *OsPR5-2*, *OsbZIP52*, and *OsRbohE* were not changed. *OsPR5-1* and *OsPR1* were increased in RBSDV- and RSV-infected rice, respectively (Additional file 1: Fig. S3B and S3C). These results indicated that the activated JA pathways mainly depend on the markedly increased expression of biosynthesis-related *OsLOX8* and *OsLOX9*, and the up-regulated *OsPR1a*, *OsWRKY28*, *OsPR2*, and *OsPR5-4* responsive genes.

For the SA pathway, we investigated the biosynthesis-related genes *OsICS1*, *OsPAL*, *OsPAL1*, *OsAIM1*, *OsCM*, and *OsEDS1*) and the response genes (*OsPR1-101*, *OsWRKY45-1*, *OsWRKY45-2*, *OsSGT1*, *OsPR1b*, *OsPR1a*, *OsPR1-12*, *OsPR1-21*, *OsPR1-22*, *OsPR1-51*, and *OsPR1-121*). *OsICS1*, *OsPAL*, and *OsPAL1* were significantly suppressed in both the RBSDV- and RSV-infected plants, *OsCM* and *OsEDS1* were not altered, and only *OsAIM* was slightly up-regulated in RSV-infected plants (Additional file 1: Fig. S4A). These results indicated that SA biosynthesis was suppressed upon virus infection. *OsWRKY45-1*, *OsWRKY45-2*, and *OsRR1-51* were significantly increased in both the RBSDV- and RSV-infected plants, whereas the *OsSGT1*, *OsPR1-21*, *OsPR1-22*, and *OsPR1-121* were dramatically decreased. *OsPR1-101* and *OsPR1a* were markedly up-regulated in RSV-infected plants but significantly down-regulated in RBSDV-infected plants (Additional file 1: Fig. S4B and S4C). These results suggested that the SA pathway was suppressed by virus infection through down-regulation of the main biosynthesis genes.

For the ABA pathway, qRT-PCR revealed that *OsNCED3* was significantly up-regulated in RBSDV-infected samples, whereas in RSV-infected plants, *OsNCED1-1* was up-regulated. *OsABA* was suppressed in both RBSDV- and RSV-infected plants. Other biosynthesis-related genes, including *OsABA1*, *OsbZIP72*, and *OsbZIP23*, were unchanged in RBSDV- and RSV-infected plants (Additional file 1: Fig. S5A). ABA deactivation genes *OsABA8OX1*, *OsABA8OX2*, and *OsABA8OX3* were significantly up-regulated in RBSDV-infected plants. In RSV-infected plants, *OsABA8OX2* and *OsABA8OX3* were slightly decreased (Additional file 1: Fig. S5B). The collective results indicated that the ABA pathway was activated by RBSDV infection, but was suppressed in RSV-infected plants.

For the Auxin pathway, the relative expression levels of auxin-related metabolic genes were affected differently by virus infection. Of the analysed biosynthesis genes, *OsYUCCA1*, *OsYUCCA5*, *OsYUCCA6*, *OsYUCCA9*, *OsAO-2*, *OsAO3-L*, and *OsAOO3-2* were significantly down-regulated upon RBSDV and RSV infection, whereas *OsYUCCA8*, *OsAO-1*, *OsAAO*, and *OsAAO3-1* were up-regulated (Additional file 1: Fig. S6A). The *OsGH3.8* and *OsGH3.2* auxin transformation genes were markedly increased in both RBSDV- and RSV-infected plants (Additional file 1: Fig. S6B). The *OsPIN1A*, *OsPIN1B*, *OsPIN2*, *OsPILS7a*, and *OsPILS7b* auxin transportation genes were markedly down-regulated in RBSDV- and RSV-infected plants (Additional file 1: Fig. S6C), while only *OsPIN3A* was increased (Additional file 1: Fig. S6D). The *OsIAA3* and OsIAA7 signalling genes were markedly decreased in both RBSDV- and RSV-infected plants (Additional file 1: Fig. S6E). The other signalling gene (*OsIAA20*) was significantly up-regulated in RBSDV-infected samples, whereas it was severely suppressed in RSV-infected plants (Additional file 1: Fig. S6E). These results showed that the auxin pathway was suppressed in both RBSDV- and RSV-infected plants.

For the CTK pathway, genes responsible for CTK biosynthesis, including *OsIPT3* and *OsIPT7*, were dramatically decreased in both RBSDV- and RSV-infected plants (Additional file 1: Fig. S7A). In contrast, the CTK transformation genes or oxidase genes (*OsCKX4* and *OscZOGT1*) were significantly up-regulated in both RBSDV- and RSV-infected plants (Additional file 1: Fig. S7B). The *OsPR8*, *OsPR10a-1*, *OsPR1a-1*, *OsPR1b*, and *OsPR10a-2* CTK responsive genes were markedly increased in both RBSDV- and RSV-infected plants (Additional file 1: Fig. S7C & S7D). Most of the analysed genes (*OsPR2-1*, *OsPR2-2*, *OsPR3*, *OsPR4*, *OsPR5-1*, and *OsPR6*) were suppressed (Additional file 1: Fig. S7D). Other responsive genes, which included *OsPR1a-2*, were dramatically upregulated in RBSDV-infected samples, but not in RSV-infected samples. The expression profile of *OsPR9* was completely opposite (Additional file 1: Fig. S7D). These results showed that the CTK pathway in rice was suppressed by viral infection.

For the ET pathway, the *OsACS2* biosynthesis gene was significantly up-regulated in both RBSDV- and RSV-infected plants, as was *OsACS1* in RBSDV-infected plants (Additional file 1: Fig. S8). Most of the oxidase genes (*OsACO7*, *OsACO1*, and *OsACO2*) were dramatically decreased in both the RBSDV- and RSV-infected plants (Additional file 1: Fig. S8). The collective results indicated that the ET pathway was activated in rice upon virus infection.

For the BR pathway, 24 genes, including 14 biosynthesis-related genes and 10 signalling genes, were analysed. Most of the biosynthesis genes (*OsD11*, *OsDS11-L*, *OsCPD1*, *OsGSK2*, *OsRAV1*, *OsRAV2*, *OsRAVL1*, and *OsBZR1*) were markedly suppressed in both RBSDV- and RSV-infected plants, whereas *OsDWARF4* was significantly up-regulated (Additional file 1: Fig. S9A). The four signalling genes (*OsBRI1-1*, *OsBRI1-2*, *OsBAK1-4*, and *OsBAK1*-*10*) were dramatically decreased in RBSDV- and RSV-infected plants. The other four genes (*OsI-BAK1*, *OsBAK1-2*, *OsBAK1-3*, and *OsBAK1-8*) were severely downregulated, and two genes (*OsBAK1-6* and *OsBAK1-9*) did not change (Additional file 1: Fig. S9B). The expression profiles of these genes were synchronously changed in the RBSDV- and RSV-infected plants. These results suggested that the BR pathway was suppressed in rice plants upon virus infection.

Seven phytohormone metabolism-related genes, including 154 genes, were analysed by qRT-PCR (Additional file 1: Fig. S10A). Of these genes, m^6^A modification was detected in 37 and 19 genes in RBSDV- and RSV-infected samples, respectively. m^6^A methylation was detected in nine genes in RBSDV- and RSV-infected plants (Additional file 1: Fig. S10B, Additional file 2: Table S14). m^6^A-IP-sequencing data mapped these m^6^A methylation sites to different regions of target genes. Most of the peaks were located in the coding sequence (CDS) region (Additional file 1: Fig. S10C). Furthermore, we analysed the relationship between m^6^A methylation sites on gene bodies and the relative gene expression levels. In most cases, the target gene was downregulated if the m^6^A site was located in the 5′-UTR, CDS, and CDS/3′-UTR, with the location in the 3′-UTR often being associated with up-regulation of the target gene (Additional file 1: Fig. S10D). Hence, a viral infection could regulate the expression of target genes, and the regulation mode was varied in different manners for the same genes under the two different viral infection conditions.

### Widely integrated analyses of the relationship between m^6^A methylation regions and expression levels using RNA-seq and m^6^A-IP-seq

To investigate the relationship between the location sites of m^6^A methylation and the relative expression profiles, we analysed the common regulated peaks that appeared in both the RBSDV- and RSV-infected plants. We classified the reads that extended 5′-UTR to the start codon (5′-UTR/CDS), reads that extended the stop codon to the 3′-UTR (CDS/3′-UTR), and other reads that did not go beyond two obviously distinct regions to the corresponding 5′-UTR, CDS, or 3′-UTR (Additional file 2: Table S15).

In total, 3,734 and 3,336 peaks were analysed in RBSDV- and RSV-infected plants, respectively (Additional file 1: Fig. S10E and S10F). In RBSDV-infected plants, the relative expression level of 44.45% m^6^A modified genes was not changed, 29.73% were downregulated, and 25.82% were up-regulated. Most m^6^A sites were located in the CDS and 3′-UTR regions (Additional file 1: Fig. S10E). The number of genes that were upregulated, downregulated, and unchanged were increased gradually when the m^6^A methylation occurred in the 5′-UTR/CDS and CDS/3′-UTR region (Additional file 1: Fig. S10E). In RSV-infected plants, the majority of m^6^A modified genes (69.04%) were downregulated. Most m^6^A sites (49.34%) were located in the CDS region and 3′-UTR. CDS and 3′-UTR location of the m^6^A modified sites often indicated that the target genes were downregulated (Additional file 1: Fig. S10F), and the m^6^A methylation was mostly occurred in CDS and 3′-UTR region in both RSV- and RBSDV-infected samples. Taken together, these results indicated that the regulatory roles of post-transcriptional m^6^A modification differed upon RBSDV or RSV infection of rice plants and that certain m^6^A sites on specific genes may have specific functions. The common regular pattern between expression level and m^6^A methylation region, which are suitable for all m^6^A methylated genes, have not been found so far. This may be due to the fact that different viruses cause plant disease through hijacking different host’s signal pathways, and different genes own different sequences characteristics.

## Discussion

The discovery of the m^6^A modification in different organisms has revealed a new and promising area of research in RNA epigenetics [4, 29, 31]. The understanding of the epigenetic roles of m^6^A modifications in eukaryotes has just begun. In our studies, to deepen the understanding of the fundamental roles of m^6^A in rice plants and the interactions between rice and viruses, we analysed the genome-wide m^6^A distributions of the transcriptome using the rice cultivar HD5 infected with RBSDV and RSV. We determined a series of genome-wide m^6^A distribution maps using m^6^A-IP data that corresponded to viruses and rice plants. These sites were identified by independent m^6^A-IP-qPCR and nucleic acid-based dot-blot assays using an m^6^A specific antibody and by quantitative LC-MS/MS. qRT-PCR comparison with transcriptome data permitted the comprehensive analysis of the relative expression levels of important antiviral pathway-related genes in rice. The m^6^A regulation mode was systematically analysed by correlating the positions of m^6^A modification on the gene body and the relative expression levels of target genes. These analyses have provided the first data on the genome-wide m^6^A modification distributions on rice, RSV, and RBSDV. Most of the consensus motifs were enriched in rice with different treatments. m^6^A modification was activated in virus-infected rice plants. m^6^A modification was strongly associated with genes in rice that were expressed at low levels upon virus infection. m^6^A modification plays essential roles in the regulation of the relative expression of the main antiviral pathway genes. The mode of regulation of gene expression by m^6^A modification was different for RBSDV and RSV infections.

### High-throughput transcriptome-based genome-wide m^6^A distributions

m^6^A modification distribution maps of virus infection and the associations with host gene expression and virus infection have already been determined in mammalian cells. Differences in these maps upon infection with different viruses have been described [6, 11, 35, 50]. In plants, mapping m^6^A sites in the gene bodies of mRNAs has been investigated in *Arabidopsis* [17, 20, 32, 51–53]. However, little has been reported in maize [15] and rice [54]. Presently, genome-wide m^6^A distribution maps of rice leaves and in rice infected with RSV and RBSDV were obtained using high-throughput transcriptomic-based m^6^A-IP sequencing (Fig. 2). In total, we obtained > 20,000 unique m^6^A peaks in each treatment, which exceeds the numbers previously reported in callus and leaves (approximately 8000 and 14,000, respectively) [54]. Most of the peaks were common in mock-, RBSDV-, and RSV-infected rice, but some m^6^A peaks were randomly distributed and different in RBSDV- and RSV-infected rice, and the common m^6^A peaks were mainly distributed in rice chromosomes 1, 2, and 3 (Fig. 2). Besides, some common m^6^A peaks were simultaneously and specifically distributed in chromosomes 4, 5, and 10 in RBSDV- and RSV-infected rice (Fig. 2). In addition, the m^6^A peak density was high in telomeric regions in rice chromosomes 2, 3, 4, 9, and 10 (Fig. 2). These results imply that post-transcriptional m^6^A modification may play different roles in different viral infection conditions and chromatin conformation alternations. The chromatin state and transcription regulatory function of m^6^A modification in chromosome-associated regulation was revealed recently [4]. However, the detailed and real biological functions of m^6^A modification in plants remain largely unknown and require further study.

In animal viruses, the m^6^A modification of genomic RNA was determined several years ago. This modification plays significant roles in virus infection, replication, and gene expression processes [11, 33, 35, 50]. However, in plant viruses, the m^6^A modification distribution on viral genomic RNA is obscure. Only two plant viruses, AMV and TMV, are reportedly involved in m^6^A modification in *Arabidopsis* and tobacco, respectively, [33, 36]. In *Arabidopsis*, protein interactions between AMV CP and atALKBH98 (Eraser) resulted in successful infection, while the increased abundance of m^6^A in the AMV genomic RNA impaired systemic infection in conditions of AtALKBH98 suppression [23]. In tobacco, TMV infection reduced m^6^A levels by up-regulating the potential demethylase and down-regulation of the potential methylase [36]. The collective findings suggest that m^6^A has an important regulatory role in interactions between plants and viruses. However, the details remain unknown. We focused on the two most important and devastating viruses concerning rice production (RBSDV and RSV) to try to understand whether m^6^A was involved in the rice-virus interactions. The two genome-wide m^6^A distribution maps of RBSDV and RSV (Fig. 3) are novel. The precise and detailed positions of m^6^A sites on viral genomic RNA, especially RBSDV segment S5, S6, and S9 (Fig. 3C), or RSV RNA1, RNA2, and RNA3 (Fig. 3D). Although previous studies have shown that m^6^A modifications participate in plant virus infections, the precise m^6^A positions on the genomic RNA were not revealed. Our study is the first to show the genome-wide m^6^A distributions in the RBSDV and RSV. The candidate potential m^6^A sites may permit for a deeper understanding of the roles in virus infection or interactions between viruses and plants, especially in studies of relationships between RNA virus genomic structures and their infectivity. However, we located the m^6^A site only in a genomic region of approximately 200 bp, which was not sufficiently accurate and precise. Further studies are needed to identify the single nucleotide that was methylated. With the recent development of nanopore sequencing technology [32, 55], accurate and fine genome-wide m^6^A distribution mapping should be possible.

### Most common consensus motifs assay

Earlier biochemical studies, high-throughput transcriptome-based m^6^A-IP sequencing, and recent nanopore sequencing of animal, viral, and plant mRNAs have shown that the consensus sequence RR[m^6^A]CH (R=A/G, H=A/C/U) is the most significantly enriched motif in m^6^A peaks in all eukaryotes to date [5, 15, 32, 56–59]. In agreement with these reports, we identified 114,546, 104,757, and 117,381 canonical RRACH motifs in mock-, RBSDV-, and RSV-infected samples, respectively. These accounted for 67.9%, 68.9%, and 67.8% of the respective total m^6^A peaks obtained from sequencing data (Additional file 2: Table S10). Additionally, we analysed the four other most common consensus motifs in rice (“URUAY”, “RAGRAG”, and “UGUAMM”, R=A/G, H=A/C/U, W=A/U, M=A/C) [14, 54] (Additional file 2: Table S10). These motifs also were significantly enriched in the three aforementioned treatments, implying that our sequencing data were confident and believable.

In the enriched m^6^A peaks (FC > 1.5, *p* < 0.05), we obtained the most common motifs using *de novo* prediction software for mock-, RBSDV-, and RSV-infected rice samples (Fig. 7). The “CGXCGXC” (X=A/U/C/G) motif was the most enriched motif in the significantly enriched m^6^A peaks in mock- and RBSDV-infected rice plants. The “CGXCGXCG” (X=A/U/C/G) motif was enriched in RSV-infected rice. These *de novo* predicted motifs may indicate potential consensus around the methylated adenine site that is common in the absence and presence of virus infection. Searches for other functional common consensus motifs for m^6^A modifications need to be performed.

### Activation of m^6^A modification in rice under biotic stress

The potential functions of m^6^A epigenetic alterations in various eukaryotes and viruses, which are involved in RNA metabolism [29], plant development regulation [24], and RNA stability regulation [51], have been elucidated. However, the detailed and precise roles of m^6^A are obscure. Presently, the m^6^A modification process was activated upon virus infection in rice, as evidence by the viral infection associated increase of m^6^A peak number (Fig. 4A), the differential m^6^A peak number (Fig. 4C and Additional file 2: Table S6), and the m^6^A content (Fig. 8A, B). These results revealed the positive association of m^6^A with plant virus infection.

Viral infection in rice plants is a biotic stress. The m^6^A modification status during this stress was presently clarified for RSV and RBSDV. We investigated the relative expression of two candidate genes (*OsAGO18* and *OsSLRL1*) and validated their m^6^A regions on the gene body using m^6^A-IP-qPCR with corresponding primers (Fig. 8C–F). The m^6^A modifications were strongly associated with the up- (Fig. 8C, E) or downregulation (Fig. 8D, F) of the target genes, which determined the direct correlation between m^6^A modification and relative expression. However, compared with the decreased m^6^A levels evident during TMV infection of tobacco [36], we obtained evidence using different examinations of the opposite result. The dichotomy may have resulted from the different virus infections in different plants. Viral characteristics are very distinct from each other, and their lifestyle in different hosts are often varied [60]. Therefore, different viruses have different effects on the host’s m^6^A modifications.

### Regulation of the relative expression levels of target genes

Key discoveries of mRNA m^6^A modification functions have mostly concentrated on development [12, 24], such as embryo formation, growth, organ definition [61], apical dominance, trichome branches, gravitropic response, development of lateral roots and vasculature [18, 62], stem cell differentiation in the shoot apical meristem [17], maintenance of circadian and seasonal plant rhythms [32], and flowering [20], all these processes occurred due to the temporal and spatial expression of the key genes in development. Hence, how does m^6^A methylation regulate the expression of these key genes is crucial in its molecular functional dissections.

In most cases, the post-transcriptional gene regulations are considered to result from host small RNA-based regulation, which mediate the rapid change of relative expression of key pathway genes for better adaptation under stress [63]. Virus infection in rice plants is an important biological stress that alters (in an as yet unclear manner) the expression profiles of genes responsible for defence against the viral pathogen. However, the main group of genes that are m^6^A modified are unknown, as are the m^6^A modifications that participate in the response to the infection. The detailed mode of regulation of m^6^A modifications to the target mRNA are also unclear. Presently, we first focused on the statistical data of the relationship between the gene expression levels and m^6^A modification. The results showed that m^6^A mostly occurred in genes that were expressed at low levels during virus infection in plants (Fig. 5), and no other reports about this information in plants under biotic stress. Under abiotic stress conditions, the stability of salt-tolerant transcripts was enhanced by adding the m^6^A sites for tolerance improvement in salt-stressed *Arabidopsis* [51]. The WRITER (ALKBH10 family) and READER (ECT2 family) genes were increased, whereas the overall levels of m^6^A modifications were decreased in maize under drought stress [15]. The READER ECT2 was relocated to the stress granules in heat-stressed cells [24, 64]. These results indicate that the responsive m^6^A modifications are sensitive and complex under abiotic stress. Similarly, the present findings also indicate sensitivity and complexity of m^6^A in response to biotic stress, especially in genes expressed at low levels.

More specifically, several targeted pathways that were strongly associated with virus infections were further explored. We analysed the relative expression levels of the m^6^A modification machinery-related genes (Fig. 9A–D), the main antiviral RNA silencing pathway-related genes (Fig. 10A–C), potential resistance genes (Fig. 10D), and seven phytohormone metabolic pathway-related genes (Additional file 1: Fig. S3–S9) using qRT-PCR. Combined with m^6^A-IP-sequencing data, the m^6^A modifications were strongly associated with these pathways (Figs. 9E and 10E, Additional file 2: Table S14). In addition, we found that m^6^A modification was also involved in the top five KEGG pathway-related genes that originated from the RNA-seq data (Additional file 2: Table S9, S11, S12, S16, and S17). The collective results indicate that m^6^A actively participates in interactions between plants and viruses and has essential roles in the regulation of the relative expression of target genes.

With respect to the main components of the m^6^A modification machinery, we found that six component genes of the m^6^A machinery for RBSDV and RSV could be modified by m^6^A (Fig. 9E). Their relative expression levels also varied (Fig. 9A–D). These results suggest that the m^6^A machinery could regulate the expression of main component genes through m^6^A methylation, which influences the overall cellular m^6^A levels of the host under conditions of viral infection. In previous studies, the WRITER genes were downregulated, whereas the ERASER genes were down-regulated in TMV infection of tobacco [36]. The findings similarly showed the regulatory roles of virus infection in the main m^6^A machinery components. Suppression of *AtALKBH9B* expression, the systemic infection of *Arabidopsis* with AMV was impaired, while CMV infection did not influence [23]. Together with our findings, these results imply the potential of m^6^A modification in regulating plant antiviral defence processes.

As the main plant antiviral strategies, RNA silencing pathways and resistance genes play significant roles in countering virus infections [65, 66]. Presently, we first analysed the relative expression levels of nine *OsDCLs* genes, five *OsRDRs* genes, 17 *OsAGOs* genes, and nine reported resistance genes in rice infected with RBSDV or RSV (Fig. 10A–D). Based on the m^6^A-IP-sequencing date, we mapped the detailed m^6^A modified genes involved in the RNA silencing pathways (Fig. 10E). Surprisingly, many essential genes, including *OsAGO18* [40] and *OsAGO2* [67], could be m^6^A modified, and their relative expression levels were also affected. In addition, other RNA silencing pathway-related genes were also m^6^A modified during infection with RBSDV or RSV. Except for the small RNA-based gene expression regulatory strategies in plants, our results provide the first evidence that m^6^A could act as an important regulator of gene expression to participate in the regulation of the main antiviral RNA silencing pathway-related genes.

Phytohormones are the main chemical messengers in the regulation of the physiological processes from germination to senescence during the whole life cycle of plants. Phytohormones have significant roles in defending against biotic and abiotic stresses in plants, especially against viral infections [49, 68]. RBSDV and RSV are two important and devastating rice pathogens. These virus infections occur at epidemic proportions and severely affect the quality and yield of rice in the main production area of East Asia [69]. In recent years, that different types of phytohormones balancing plant growth and antiviral defence processes in plants have been demonstrated. They are JA [40, 70–75], SA [45, 71, 72], ABA [71], auxin [72], CTK [71], ET [70], and BR [40, 70, 74, 76], respectively. All these results imply that these seven phytohormones widely participate in the regulation of the physiological status of the host plant to coordinate response to virus infections. Similarly, we wanted to determine whether m^6^A modifications are also involved in regulating the metabolism of these seven phytohormones. First, the relative expression of these seven phytohormone metabolism pathway-related genes were determined by qRT-PCR (Additional file 1: Fig. S3–S9). Furthermore, m^6^A-IP sequencing identified the genes that were m^6^A modified (Additional file 2: Table S14). The data demonstrated that the m^6^A methylated genes were distributed among nearly all analysed specific phytohormone metabolism pathways during virus infection. These results imply the frequent and important roles of m^6^A modification in interactions between plants and viruses, especially in the use of phytohormones in defence. Numerous reports have described the relationships between specific phytohormones and virus infection. The present description of the m^6^A modification-mediated gene expression regulatory strategy in phytohormone metabolism is the first time.

Besides the analysed host m^6^A machinery, RNA silencing pathways, and seven phytohormone metabolism pathways, we further explored the top five KEGG pathways obtained by differentially expressed genes in RBSDV- and RSV-infected rice plants compared with mock treatment (Additional file 2: Table S11, S16, and S17). KEGG pathway analyses showed that phenylpropanoid biosynthesis (ko00940), plant hormone signal transduction (ko04075), amino sugar and nucleotide sugar metabolism (ko00520), starch and sucrose metabolism (ko00500), and cysteine and methionine metabolism (ko00270) pathways were differentially regulated under RBSDV and RSV infection of rice, with a total of 21 common genes methylated (Additional file 2: Table S17). Combined with the results of the pathways that correlated with virus infection, the collective findings of these analyses once again demonstrated that the m^6^A modification participates in almost every aspect of host biological process, especially the host’s responsive signal transduction of viral infection.

Taken together, the post-transcriptional mRNA m^6^A modifications, which were often unrecognised in previous gene expression analyses, play important roles in interactions between host and virus. The results imply that the general or possibly regulatory machinery of the up- or down-regulation of target genes in certain conditions could involve in m^6^A modifications, in addition to small RNA-based transcriptional regulation, RNA decay, and DNA transcriptional suppression.

### Regulation-mode of the relative expression of target genes

Mediation of mRNA physiological status by m^6^A modification leads to fate determination and has been correlated with a wide range of mRNA metabolism, including changes in the secondary structure [26, 77–79], nuclear export [30, 80], pre-miRNA splicing and processing [26, 29, 78, 81–84], alternative polyadenylation site (APA) choice [85–87], RNA stability [17, 24, 88], and RNA maturation [14, 18, 61, 88]. The relationship between m^6^A modifications and mRNA translation has been intensively studied in recent years [2, 13]. Several groups demonstrated the stimulatory effects of m^6^A on translation [89–93], while others groups showed inhibitory effects [94–96]. The dichotomous effects of m^6^A on translation indicate the involvement of other, as yet unclear, factors. In terms of detail sites, m^6^A modification mainly affects the secondary structures of target RNA due to the addition of the methyl group to position 6 of adenylate at special gene body, and these chemical structures’ changes lead to other biological or activity alterations. Understanding the impact of the m^6^A modification on the relative expression levels of transcripts was the basis to uncover the mechanism of m^6^A translation.

To determine some regular pattern of m^6^A modifications to the transcripts’ relative expression levels, we first mapped the m^6^A modification sites on the gene body and then obtained data of the regulation pattern by using the m^6^A positions and their corresponding expression profiles from m^6^A-IP-seq and RNA-seq/qRT-PCR, respectively (Additional file 1: Fig. S10). Seven phytohormone metabolic pathway-related genes comprising a total of 154 genes, were used for m^6^A and relative expression profile determination analyses (Fig. 10A–D). m^6^A modification could lead to down-regulation of the target genes in most cases, especially when the m^6^A modification site was located in the 5′-UTR or CDS/3′-UTR region of the target gene. Previous studies, especially in animal systems, showed that higher levels of m^6^A modification in the last exon may affect the usage of APA [85], while Bartosovic et al. confirmed that the length of the 3′-UTR could be regulated by the regulation of APA in similar situations [86]. In plants, the rice mutant with disabled FIP37 (WRITER) resulted in chimeric mRNA formation of the two spatially adjacent genes (pair of AT4g30570/580 or AT1g71330/340) [17, 97]. These results confirmed the potential functions of m^6^A in APA alternatives, which directly determine the translational levels of the target genes. Our finding of the m^6^A location of the CDS/3′-UTR region may support the reported APA alternative mechanism from the perspective of transcription, and another finding of m^6^A location of the 5′-UTR regions, which supports that 5′-UTR m^6^A locations result in higher translational efficiency [13].

To ascertain the regulation pattern between positions of m^6^A modification sites and the target genes’ expression profile, we integrated the m^6^A-IP-seq data. We selected the common differentially regulated m^6^A peaks of both RBSDV- and RSV-infected rice samples that were compared with the mock treatments. Then, we annotated and mapped these differently regulated peaks to the corresponding target genes and obtained 2913 and 2624 genes in RBSDV- and RSV-infected rice samples, respectively (Additional file 2: Table S15). As shown in Fig. 10E, F, wherever the m^6^A modification sites were located, the target gene transcripts in most cases were downregulated (Fig. 10F) or unchanged (Fig. 10E), especially in the RBSDV-infected samples, consistent with our findings from m^6^A modifications in seven phytohormone metabolic pathway-related genes (Fig. 10A–D). The RSV-infected samples displayed smaller changes in gene expression under conditions of m^6^A modifications in any position of the target genes (Fig. 10E). All these results imply that m^6^A could directly regulate the secondary structures of mRNA by affecting APA usage and alternations. The results also indicate that infections by different viruses could coordinate and regulate different physiological states of plants for the best viral adaptation to host defence, to achieve a balance between plant defence and viral infection.

The present study is the first description of genome-wide m^6^A distribution maps of rice and of two significant viruses. We further analysed the common consensus motifs and validated the m^6^A modification in rice. m^6^A was activated in RBSDV- and RSV-infected rice, and the activation was predominantly in genes that were not actively expressed. Finally, m^6^A was strongly associated with and directly regulated the expression of the main antiviral pathway-related genes in rice plants. All these findings deepen our understanding of the significant roles of m^6^A in interactions between plants and viruses, especially the model plant (rice) and two significant model plant viruses (RBSDV and RSV). The findings highlight the need for more profound and detailed investigations of the functions of m^6^A on select target genes.

## Method and materials

### Plant materials

The rice japonica cultivar Huaidao 5 (HD-5) was planted in a bucket and cultured in a growth chamber under short-day (SD) conditions (11 h light/13 h dark) with a light intensity of 900 μmol/m^2^ s^1^ at 27 °C. HD-5 was highly susceptible to rice stripe virus or rice black-stripe dwarf virus in the field [98].

### Artificial inoculation of RBSDV and RSV

Artificial inoculation of RBSDV and RSV was performed as previously described with minor modifications [99, 100]. For RSV inoculation, small brown planthoppers (SBPHs) that carring RSV, which was maintained at the Department of Plant Protection, Yangzhou University, was transferred to rice seedlings cultured in a glass beaker (1 L). Then, the seedlings were divided and transplanted to a 20 L plastic bucket after 3 days’ aspiration. For RBSDV inoculation, RBSDV-infected rice plants were collected from the field and exposed to non-virulent SBPHs for 4 days. As the SBPHs fed, they acquired the RBSDV. The viruliferous SBPH were transferred to a beaker that was cultured with rice seedlings. Three days later, the rice seedlings were transplanted to the same-size plastic bucket mentioned above.

For mock treatment, non-virulent SBPHs were transferred to a glass breaker and cultured with rice seedlings for three days. The seedlings were transplanted in a similar manner as described above for the viruliferous SBPH inoculated seedlings. The RSV- and RBSDV-infected rice plants were equipped with pest control nets and cultured in the growth chamber at Yangzhou University. Total protein was extracted from the planted rice seedlings and analysed. western blot was performed to detect the presence of RSV and RBSDV as described next.

### Western blot detection of RSV and RBSDV

Western blot detection was performed as described previously, with minor modifications [101]. Briefly, total proteins of the mock- or virus-inoculated rice seedlings were extracted and dissolved in 2 × SDS protein loading buffer containing 5% β-mercaptoethanol. After 5 min of heating in boiling water and centrifugation at 13,000 *g* for 10 min, the samples were resolved by 12.5% SDS-PAGE. The total proteins were transferred to a piece of nitrocellulose membrane. Polyclonal antiserum against RSV NS3 and RBSDV p10 protein was used as the first antibody. This was followed by a secondary alkaline phosphatase-linked antibody (AP-A; Sigma-Aldrich, USA). The specific bands were visualised with a colour reaction by adding nitro blue tetrazolium/5-bromo-4-chloro-3-indolyl phosphate (NBT/BCIP) as the substrates (Sangon Biotech, China).

### Total RNA exaction and reverse transcription-polymerase chain reaction (RT-PCR) detection

Trizol Reagent (TaKaRa Bio, Japan) was used for rice total RNA extraction as described previously [102]. RNase-free rDNase I (TaKaRa Bio, Japan) was added to remove contaminating genomic DNA. Reverse transcription reactions were performed as described previously with RSV- and RBSDV-specific primers, respectively (Additional file 2: Table S1) [101]. Synthetic cDNA was used as templates, and PCR reactions were performed with specific pairs of primers as described.

### High-throughput RNA m^6^A-IP-seq and RNA-seq

Mock, RSV-, and RBSDV-infected rice plants were used for total RNA isolation. Ribosome RNA (rRNA) was captured and deleted using the RiboMinus™ Plant Kit for RNA-seq (Thermo Fisher Scientific, USA). RNA Fragmentation Reagents (Ambion, USA) were added and the RNA was randomly fragmented into approximately 200 nucleotide fragments. The fragmented RNA was divided into two identical portions. One was used in the m^6^A-IP experiment, the other was used as the input for the next RNA-seq.

Anti-m^6^A polyclonal antibody (#ab208577; Abcam, USA) was added to the obtained fragmented RNA and incubated at 4°C for 2 h in immunoprecipitation (IP) buffer (10 mM Tris-HCl, pH 7.4, 150 mM NaCl, 0.1% [v/v] octylphenoxypolyethoxyethanol [Igepal CA-630]) with the addition of RNase Plus RNase inhibitor (Promega, USA). The mixture was immunoprecipitated by incubation with protein-A conjugated beads at 4 °C for 2 h. After adequate washing and removal of non-specifically bound compounds, 0.8 mg/mL *N*^*6*^-methyladenosine in IP buffer was used to elute the bound RNA. TruSeq standard mRNA Sample Prep Kit (Illumina, USA) was used to construct the library of immunoprecipitated RNA and input RNA as described previously [15]. High-throughput sequencing was performed on the Hiseq X Ten platform (Illumina) with the libraries corresponding to immunoprecipitated RNA. Single-end reads of 50-bp-length were obtained. The libraries from the input RNA were also sequenced and 122-bp-length double-end reads were produced.

### Analysis of the sequence data

Row reads from m^6^A-seq and RNA-seq were trimmed by adaptor sequence removal, followed by treatment with the Trimmomatic v0.36 tool [103] to remove the low-quality bases. The FastQC programme was used to examine the quality of the trimmed m^6^A-seq and RNA-seq clear reads (https://www.bioinformatics.babraham.ac.uk/projects/fastqc).

### Distribution of m^6^A sites in rice and viral genomes

The obtained RNA-seq clear reads of mock-treated, RSV-, and RBSDV-infected rice were aligned to the rice (*Oryza sativa*. IRGSP-1.0), RBSDV, and RSV reference genome, respectively, using Tophat v2.1.1 [104]. The maximum intron length was set to 10 kb with default settings for other parameters. Cufflinks v2.2.1 [105] was used to analyse the unique mapping reads. The term FPKM mapped reads (FPKM = Counts of mapped fragments × 10^9^/[length of transcript × total count of the mapped fragments]) was used for normalisation and estimation of the gene expression level. The Cuffdiff programme in Cufflinks v2.2.1 was used for differential analysis. The *O. sativa* reference genome sequences and annotation were download from Ensembl Plants (release 45; https://plants.ensembl.org)[106]. The reference genome sequences of RSV and RBSDV were obtained from the NCBI database (https://www.ncbi.nlm.nih.gov/).

### Peak calling analysis

The obtained m^6^A-seq clear reads of mock-treated, RSV-, and RBSDV-infected rice samples were aligned to their respective reference genome as described above. The m^6^A enriched peaks in each m^6^A-IP sample were determined by MeTDiff peak calling software [107] with the corresponding input sample serving as control. The running options of the MeTDiff software were set as: FRAGMENT_LENGTH = 200, PEAK_CUTOFF_PVALUE = 0.01, PEAKS_CUTOFF_FDR = 0.05. After the running, the m^6^A peak was detected, and the called peaks were annotated by intersection with gene architecture by ChIPseeker [108].

### Identification of enriched motifs within m^6^A peaks

The MEME (http://meme-suite.org/tools/dreme) [109] and Discriminative Regular Expression Motif Elicitation (DREME) [110] tools suites located in the MEME suite were used to perform motif enhancement analyses. The DREME suite was used to discover short (≤ 8 bp) and ungapped motifs enriched within series target m^6^A peak sequences relative to control sequences (shuffled m^6^A peak sequences). The series target m^6^A peak sequences were extracted from the rice reference genome (*O. sativa*. IRGSP-1.0) using the fastaFromBed function in BEDTools software v2.28 [111]. The control sequences were randomly produced by shuffling each m^6^A peak sequence while retaining the nucleotide frequencies. The performance was executed by “fasta-shuffle-letters” utility (k = 1) in the MEME suite. These two aforementioned types of sequences were loaded into DREME for discovery motifs. The parameter settings were as follows: meme-minw, 8; meme-maxw 30; meme-nmotifs, 10; *E* dreme e-value threshold, 0.05; Fimo-skip-spamo-skip-centrimo-score, 5.0; centrimo-ethresh, 10.0; The AME tools (http://meme-suite.org/tools/ame) package in the MEME suite was used to calculate the significance level of relative enrichment of one special motif within target sequences relative to the control sequences.

### Gene expression quantification

Total RNA was extracted as described previously [102]. After treatment with RNase-free rDNase I (TaKaRa Bio, Japan), the first-strand cDNA was produced using PrimeScript II First Strand cDNA Synthesis Kit (TaKaRa Bio, Japan) according to the manufacturer’s instructions. Quantitative PCR (qPCR) was performed using the CFX96 Real-Time PCR Detection System with SsoFast EvaGreen Supermix (Bio-Rad, USA). The quantitative experiment was performed with three biological replicates and three technical replicates. Rice *eEF1α* was chosen as the internal reference gene [112]. The gene expression levels were analysed using the Bio-Rad CFX Manager software. The pairs of primers used for gene expression quantification are listed in Additional file 2: Tables S18 and S19.

### Total mRNA isolation and Dot-blot analyses

The extracted total RNAs of the rice plant by Trizol Reagent (TaKaRa Bio, Japan). Then, the Ambion® Poly(A)Purist™ MAG kit (Invitrogen^TM^, USA) was taken for total mRNA isolation according to the manufacturer's instructions. RNA integrity was evaluated using the Agilent 2100 Bioanalyzer (Agilent Technologies, Santa Clara, CA, USA). Dot blotting was performed with minor modification as described previously [15]. Briefly, the extracted total mRNA was treated with DNase and quantified with a NanoDrop™ 1000 Spectrophotometer (Thermo Fisher Scientific, USA). The loading volume to each dot was calculated according to the concentration of the purified total RNA. The RNAs were denatured by heating at 75 °C for 5 min, followed by chilling on ice for at least 10 min before loading. The samples were spotted on a Hybond-N^+^ membrane (Amersham, USA), followed by drying at 25 °C for 20 min. After ultraviolet crosslinking, the membrane was blocked with 3% skimmed milk in 1 × PBST buffer (PBS containing 0.05% Tween-20) for 3 h. Then, the membrane was washed three times for 10 min interval with 1× PBST buffer. The membrane was incubated with anti-m^6^A antibody (#ab208577; Abcam) in 1× PBST buffer for 1 h at 4 °C. After washing three times, the membrane was incubated with alkaline phosphatase (AP)-conjugated anti-mouse immunoglobulin G secondary antibody (#ab98712; Abcam). The colour reaction was performed as in the western blot.

### Liquid chromatography-tandem mass spectrometry (LC-MS/MS) analysis of m^6^A contents

RNA samples isolated by the kit above were treated with a digestion cocktail containing nuclease P1 (Sigma-Aldrich, USA) and phosphodiesterase I (Sigma-Aldrich). The digestion was performed in buffer (10 mM Tris-HCl pH 6.8, 30 mM sodium acetate, 2 mM ZnCl_2_) with 2 U and 0.01 U of each corresponding enzyme at 37 °C for 12 h, followed by further digestion with 2 U AP for 1 h at 37 °C. The digestion enzymes were removed using a 10 kDa filtration tube (Amicon Ultra, USA). RNA solutions were diluted for 10 times, and 10 μL of the dilution was used for LC-MS/MS. Reverse-phase high-performance liquid chromatography (Agilent, USA) was used to separate the nucleosides using an Agilent C 18 column (5 μm size, 150 mm × 2.1 mm), which was combined with MS detection using a QTRAP 5500 device (AB Sciex, USA). The detailed parameters were set as previously described [113].

### Validation of m^6^A site using m^6^A-IP-qPCR

Total RNA was extracted from mock-, RSV-, and RBSDV-infected samples at different times (0, 1, 2, 4, 8, and 16 days post-inoculation). After rRNA removal by the Ambion® Poly(A)Purist™ MAG kit, RNA fragmentation, and m^6^A-IP experiments were performed as described above [16]. The precipitated RNAs from *N*^*6*^-methyladenosine eluted solution were used as templates. The fold enrichment of each fragment was determined by qRT-PCR. The designed specific primers are shown in Additional file 2: Table S18.

## Supplementary Information

**Additional file 1: Fig. S1** Distribution pattern of sequenced m^6^A-IP-seq reads along the transcripts. **Fig. S2** Intersection among m^6^A peaks identified in two biological replicates of three treatments. **Fig. S3** Relative expression of jasmine acid (JA) biosynthesis and response genes in rice infected with viruses. **Fig. S4** Relative expression of salicylic acid (SA) biosynthesis and response genes in rice infected with viruses. **Fig. S5** Relative expression levels of abscisic acid (ABA) biosynthesis and deactivation genes in rice infected with viruses. **Fig. S6** Relative expression levels of auxin biosynthesis, transportation, and signaling genes in rice infected with viruses. **Fig. S7.** Relative expression levels of cytokinin (CTK) biosynthesis, oxidation, and response genes in rice infected with viruses. **Fig. S8** Relative expression levels of ethylene (ET) biosynthesis genes in rice infected with viruses. **Fig. S9** Relative expression levels of brassinosteroids (BR) biosynthesis and signaling genes in rice infected with viruses. **Fig. S10** Integrated analyses of the seven main phytohormone related genes with m^6^A methylation and relative expression levels in rice infected with viruses. **Fig. S11** Loading control that corresponding to the dot-blot analyses. **Fig. S12** The uncropped western blot membranes that showed in Fig. 1B and C.

**Additional file 2: Table S1.** Primers used for RSV and RBSDV detections in this study. **Table S2**. Sequenced and rice genome mapped reads in m^6^A-IP-seq, input RNA-seq rice samples. **Table S3**. Nucleotide localization and enrichment of the top 10 m^6^A peaks identified in Mock-, RSV-, and RBSDV-infected rice transcripts by m^6^A-IP-Seq. **Table S4**. The category of the differential m^6^A peaks upon two viruses' infection in rice. **Table S5**. Nucleotide localization and enrichment of the m^6^A peaks identified in RSV and RBSDV genomics by m^6^A-IP-Seq. **Table S6.** Gene ID and their fpkm analyses. **Table S7**. Different peaks statistic. **Table S8**. The m^6^A peaks appeared in different treatments. **Table S9.** The most abundant consensus motif in Mock, RSV-, and RBSDV-infected rice plant using suite of DREME and MEME. **Table S10**. Analyses of the m^6^A peaks that containing most four common consensus appeared in other species. **Table S11.** Detail information of ListHits Gene and m^6^A methylated genes under rice viruses’ infection derived from Additional file 2: Table S16. **Table S12**. Integrated analyses of the m^6^A methylation related genes with m^6^A modifications and expression profiles. **Table S13**. Integrated analyses of the anti-viral RNA silencing pathway related genes with m^6^A modification and expression profile. **Table S14**. Integrated analyses of the plant hormone metabolic genes with m^6^A modifications and expression profiles. **Table S15**. Integrated analyses of the relationship betwwen relative expression and m^6^A positions. **Table S16**. Summary of the m^6^A RNA methylation level in enriched top 5 KEGG pathways related genes from RNA-seq under rice viruses’ infection. **Table S17**. Detail information of the common m^6^A methylated genes appeared in enriched top 5 KEGG pathways under rice viruses' infection. **Table S18**. Primers used for qRT-PCR validation of the methylation of *OsAGO18* and *OsSLRL1* genes. **Table S19**. Primers used in qRT-PCR qualification of the candidate genes.

**Additional file 3:.** Review history.

## Data Availability

All the datasets supporting the conclusions of this article are available in the Additional file 2: Tables included in this manuscript. The raw sequencing data were uploaded and deposited in NCBI (https://www.ncbi.nlm.nih.gov/bioproject/PRJNA732795) [114]. The accession number was from SRR14675282 to SRR14675293. The details were showed as below: RNA-sequencing data were SRR14675288 (Mock_Rice_1), SRR14675292 (Mock_Rice_2), SRR14675284 (RBSDV_Rice_1), SRR14675286 (RBSDV_Rice_2), SRR14675282 (RSV_Rice_1), and SRR14675290 (RSV_Rice_2), respectively. m^6^A-IP-sequencing data were SRR14675289 (Mock_Rice_1), SRR14675293 (Mock_Rice_2), SRR14675285 (RBSDV_Rice_1), SRR14675287 (RBSDV_Rice_2), SRR14675283 (RSV_Rice_1), and SRR14675291 (RSV_Rice_2), respectively. The uncropped western blot images were shown in Additional file 1: Fig. S12.
