# Supplementary material for: The dynamics of N6-methyladenine RNA modification in interactions between rice and plant viruses
Source: Genome Biol. 2021 Jun 24;22:189. doi: 10.1186/s13059-021-02410-2 (PMC8229379; doi:10.1186/s13059-021-02410-2)
Supplement: Supplementary file 1 — Additional file 1: Fig. S1 Distribution pattern of sequenced m6A-IP-seq reads along the transcripts. Fig. S2 Intersection among m6A peaks identified in two biological replicates of three treatments. Fig. S3 Relative expression of jasmine acid (JA) biosynthesis and response genes in rice infected with viruses. Fig. S4 Relative expression of salicylic acid (SA) biosynthesis and response genes in rice infected with viruses. Fig. S5 Relative expression levels of abscisic acid (ABA) biosynthesis and deactivation genes in rice infected with viruses. Fig. S6 Relative expression levels of auxin biosynthesis, transportation, and signaling genes in rice infected with viruses. Fig. S7. Relative expression levels of cytokinin (CTK) biosynthesis, oxidation, and response genes in rice infected with viruses. Fig. S8 Relative expression levels of ethylene (ET) biosynthesis genes in rice infected with viruses. Fig. S9 Relative expression levels of brassinosteroids (BR) biosynthesis and signaling genes in rice infected with viruses. Fig. S10 Integrated analyses of the seven main phytohormone related genes with m6A methylation and relative expression levels in rice infected with viruses. Fig. S11 Loading control that corresponding to the dot-blot analyses. Fig. S12 The uncropped western blot membranes that showed in Fig. 1B and C. [file 13059_2021_2410_MOESM1_ESM.pptx]

## Slide 1
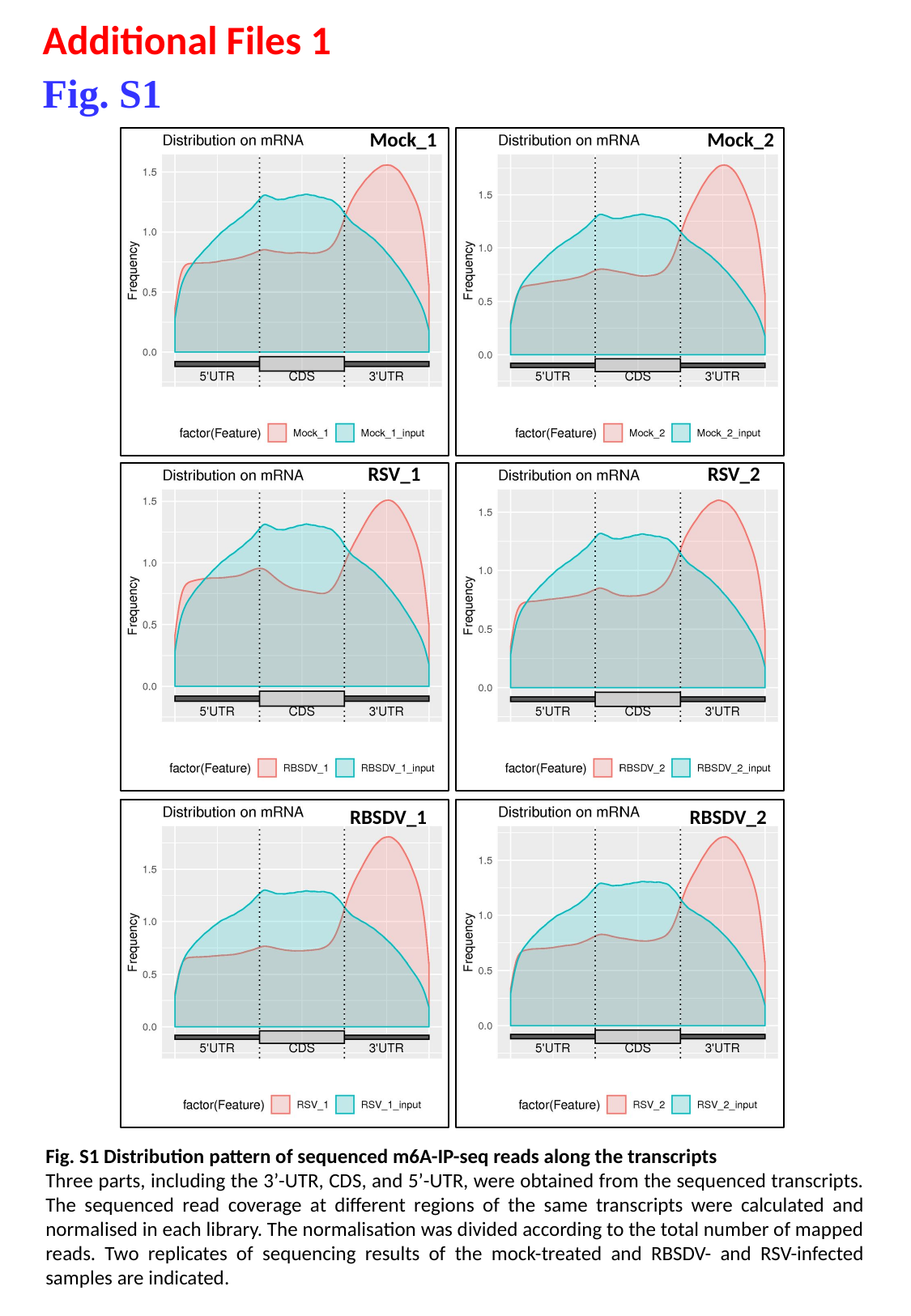

Additional Files 1
Fig. S1
Mock_1
Mock_2
RSV_1
RSV_2
RBSDV_1
RBSDV_2
Fig. S1 Distribution pattern of sequenced m6A-IP-seq reads along the transcripts
Three parts, including the 3’-UTR, CDS, and 5’-UTR, were obtained from the sequenced transcripts. The sequenced read coverage at different regions of the same transcripts were calculated and normalised in each library. The normalisation was divided according to the total number of mapped reads. Two replicates of sequencing results of the mock-treated and RBSDV- and RSV-infected samples are indicated.

## Slide 2
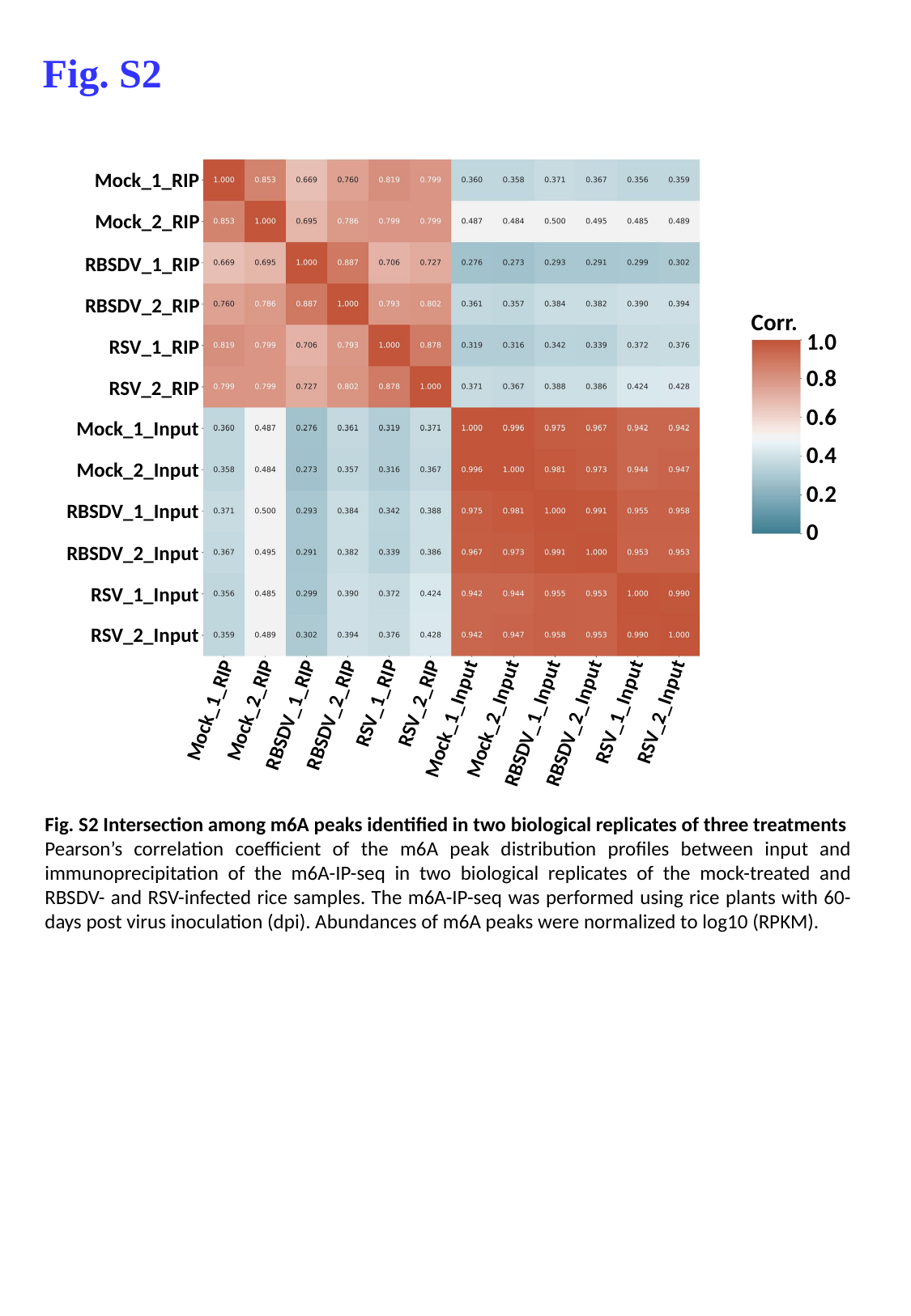

Fig. S2
Mock_1_RIP
Mock_2_RIP
RBSDV_1_RIP
RBSDV_2_RIP
Corr.
1.0
RSV_1_RIP
0.8
RSV_2_RIP
0.6
Mock_1_Input
0.4
Mock_2_Input
0.2
RBSDV_1_Input
0
RBSDV_2_Input
RSV_1_Input
RSV_2_Input
Mock_1_RIP
Mock_2_RIP
RBSDV_1_RIP
RBSDV_2_RIP
RSV_1_RIP
RSV_2_RIP
RSV_1_Input
RSV_2_Input
Mock_2_Input
RBSDV_2_Input
RBSDV_1_Input
Mock_1_Input
Fig. S2 Intersection among m6A peaks identified in two biological replicates of three treatments
Pearson’s correlation coefficient of the m6A peak distribution profiles between input and immunoprecipitation of the m6A-IP-seq in two biological replicates of the mock-treated and RBSDV- and RSV-infected rice samples. The m6A-IP-seq was performed using rice plants with 60-days post virus inoculation (dpi). Abundances of m6A peaks were normalized to log10 (RPKM).

## Slide 3
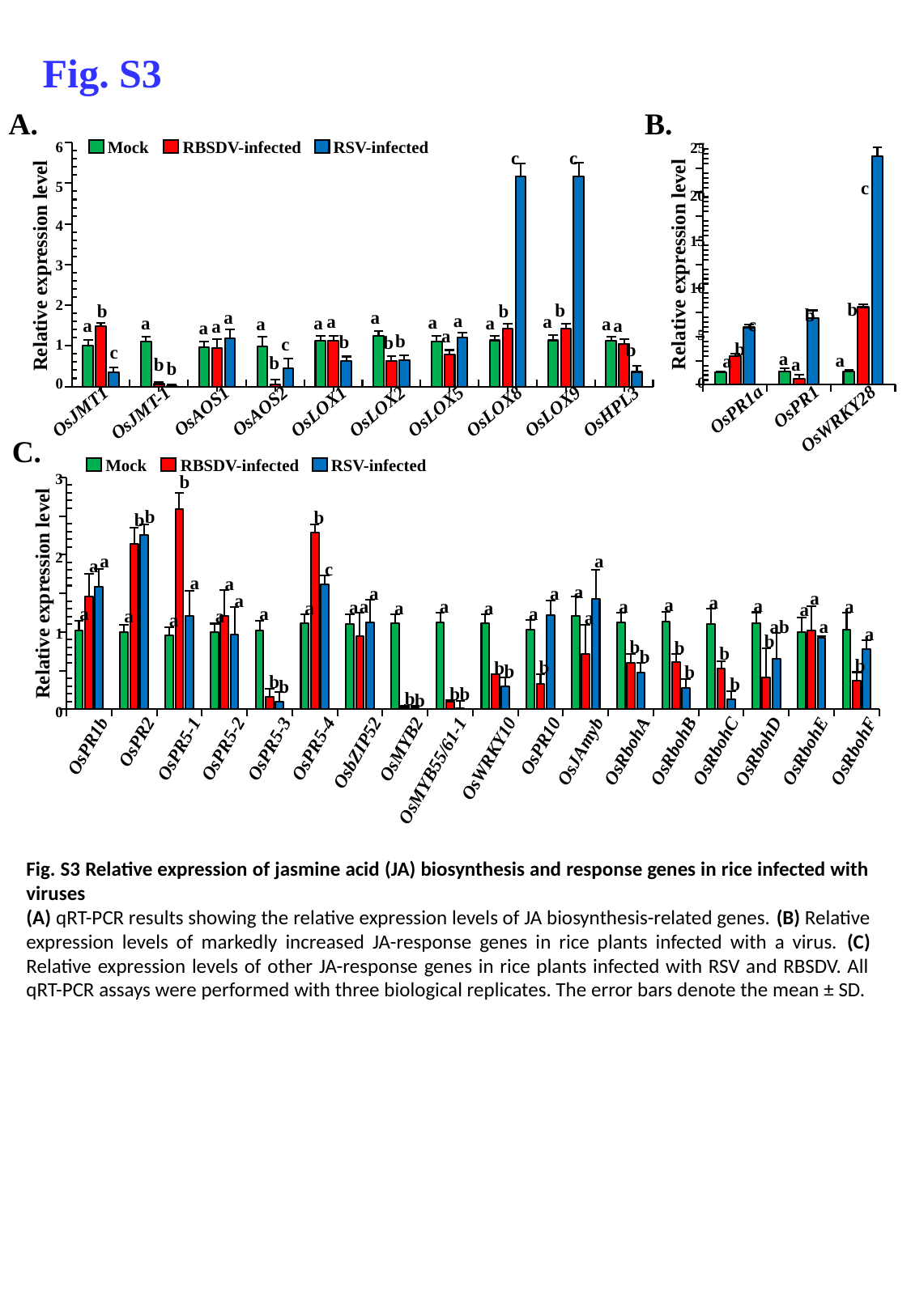

25
Fig. S3
A.
B.
### Chart
| Category | Mock | RBSDV-infected | RSV-infected |
|---|---|---|---|
| OsJMT1 | 1.012 | 1.49 | 0.345249839 |
| OsJMT-1 | 1.112 | 0.06 | 0.004065892 |
| OsAOS1 | 0.963 | 0.94 | 1.179765591 |
| OsAOS2 | 0.987 | 0.05 | 0.456794823 |
| OsLOX1 | 1.125 | 1.13 | 0.621245021 |
| OsLOX2 | 1.236 | 0.62 | 0.643632855 |
| OsLOX5 | 1.109 | 0.78 | 1.207640324 |
| OsLOX8 | 1.135 | 1.43 | 5.168813664 |
| OsLOX9 | 1.135 | 1.43 | 5.168813664 |
| OsHPL3 | 1.123 | 1.04 | 0.358878346 |
### Chart
| Category | | | |
|---|---|---|---|
| OsPR1a | 1.012 | 2.393789141 | 4.772678631 |
| OsPR1 | 1.115 | 0.480295642 | 5.556716041 |
| OsWRKY28 | 1.124 | 6.449594207 | 19.0022897 |Mock
RBSDV-infected
RSV-infected
6
25
c
c
c
5
20
4
15
Relative expression level
Relative expression level
3
10
2
b
b
b
b
b
a
a
a
a
a
a
a
a
a
a
a
c
a
a
a
a
a
5
b
b
b
c
1
b
b
c
a
a
a
b
a
b
b
0
0
OsJMT1
OsAOS1
OsAOS2
OsLOX1
OsLOX2
OsLOX5
OsLOX8
OsLOX9
OsHPL3
OsPR1a
OsPR1
OsJMT-1
OsWRKY28
C.
Mock
RBSDV-infected
RSV-infected
3
b
### Chart
| Category | Mock | RBSDV-infected | RSV-infected |
|---|---|---|---|
| OsPR1b | 1.023 | 1.453947897 | 1.582857499 |
| OsPR2 | 0.998 | 2.138305139 | 2.258847547 |
| OsPR5-1 | 0.958 | 2.588734615 | 1.21096873 |
| OsPR5-2 | 0.994 | 1.209098866 | 0.964331797 |
| OsPR5-3 | 1.023 | 0.159399999 | 0.098443501 |
| OsPR5-4 | 1.114 | 2.282653194 | 1.61191995 |
| OsbZIP52 | 1.105 | 0.947053734 | 1.119564863 |
| OsMYB2 | 1.112 | 0.02778414 | 0.022746131 |
| OsMYB55/61-L | 1.125 | 0.100830404 | 0.007984455 |
| OsWRKY10 | 1.114 | 0.451105223 | 0.292396834 |
| JIOsPR10 | 1.025 | 0.330017229 | 1.214546261 |
| OsJAmyb | 1.211 | 0.715700498 | 1.424707276 |
| OsRbohA | 1.123 | 0.600646783 | 0.478774312 |
| OsRbohB | 1.132 | 0.612631977 | 0.277448935 |
| OsRbohC | 1.102 | 0.524200145 | 0.129389057 |
| OsRbohD | 1.112 | 0.409426248 | 0.65079288 |
| OsRbohE | 0.994 | 1.023165772 | 0.926852977 |
| OsRbohF | 1.024 | 0.3643313 | 0.776360196 |OsPR10
OsPR2
OsPR1b
OsRbohE
OsRbohC
OsPR5-4
OsRbohF
OsRbohA
OsPR5-3
OsJAmyb
OsRbohD
OsRbohB
OsPR5-1
OsPR5-2
OsMYB2
OsbZIP52
OsWRKY10
OsMYB55/61-1
b
b
b
2
a
a
a
c
a
a
a
a
a
Relative expression level
a
a
a
a
a
a
a
a
a
a
a
a
a
a
a
a
a
a
a
a
a
ab
a
a
1
b
b
b
b
b
b
b
b
b
b
b
b
b
b
b
b
b
0
Fig. S3 Relative expression of jasmine acid (JA) biosynthesis and response genes in rice infected with viruses
(A) qRT-PCR results showing the relative expression levels of JA biosynthesis-related genes. (B) Relative expression levels of markedly increased JA-response genes in rice plants infected with a virus. (C) Relative expression levels of other JA-response genes in rice plants infected with RSV and RBSDV. All qRT-PCR assays were performed with three biological replicates. The error bars denote the mean ± SD.

## Slide 4
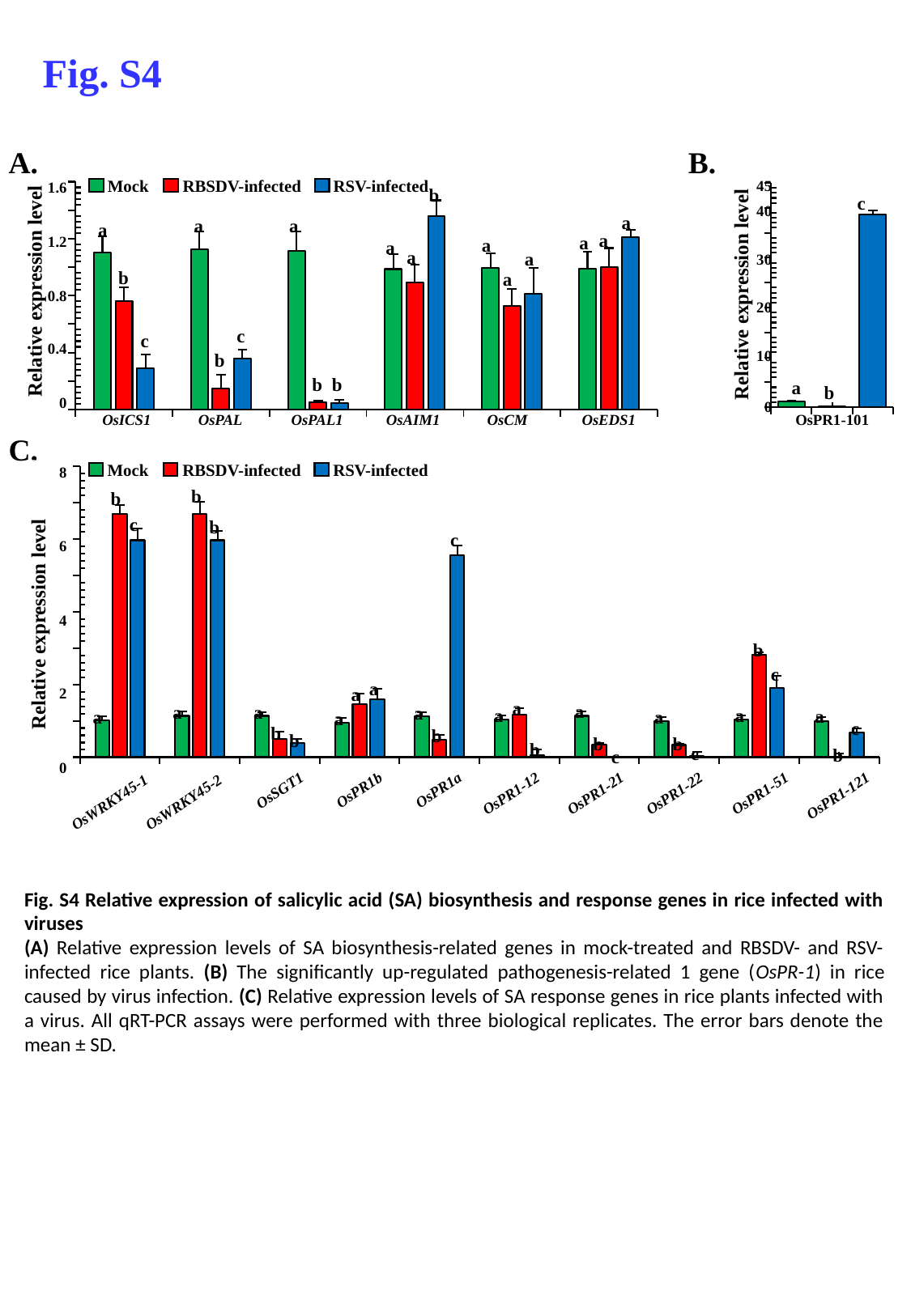

Fig. S4
A.
B.
### Chart
| Category | Mock | RBSDV-infected | RSV-infected |
|---|---|---|---|
| OsICS1 | 1.102 | 0.760231862 | 0.290958688 |
| OsPAL | 1.125 | 0.14664503 | 0.359086621 |
| OsPAL1 | 1.112 | 0.048427638 | 0.044992714 |
| OsAIM1 | 0.986 | 0.892529293 | 1.356967701 |
| OsCM | 0.995 | 0.7284337 | 0.813212289 |
| OsEDS1 | 0.987 | 0.998731032 | 1.211539207 |1.6
1.2
0.8
0.4
0
### Chart
| Category | OsPR1-101 |
|---|---|
| Mock | 1.112 |
| RBSDV-infected | 0.13739932 |
| RSV-infected | 38.7282995 |Mock
RBSDV-infected
RSV-infected
45
b
c
40
a
a
a
a
a
a
a
a
a
a
30
b
a
Relative expression level
Relative expression level
20
c
c
10
b
b
b
a
b
0
OsICS1
OsPAL
OsPAL1
OsAIM1
OsCM
OsEDS1
OsPR1-101
C.
### Chart
| Category | Mock | RBSDV-infected | RSV-infected |
|---|---|---|---|
| OsWRKY45-1 | 1.023 | 6.69874134 | 5.970849838 |
| OsWRKY45-2 | 1.135 | 6.69874134 | 5.970849838 |
| OsSGT1 | 1.135 | 0.495909376 | 0.39898287 |
| OsPR1b | 0.956 | 1.453947897 | 1.582857499 |
| OsPR1a | 1.125 | 0.480295642 | 5.556716041 |
| OsPR1-12 | 1.025 | 1.174154598 | 0.064946868 |
| OsPR1-21 | 1.136 | 0.354774045 | 0.0 |
| OsPR1-22 | 0.985 | 0.332956443 | 0.026031341 |
| OsPR1-51 | 1.025 | 2.824170944 | 1.910471612 |
| OsPR1-121 | 0.984 | 0.0 | 0.679687182 |Mock
RBSDV-infected
RSV-infected
8
b
b
c
b
c
6
4
Relative expression level
b
c
a
a
2
a
a
a
a
a
a
a
a
a
a
a
c
b
b
b
b
b
b
c
b
c
0
OsWRKY45-1
OsWRKY45-2
OsSGT1
OsPR1b
OsPR1a
OsPR1-12
OsPR1-21
OsPR1-22
OsPR1-51
OsPR1-121
Fig. S4 Relative expression of salicylic acid (SA) biosynthesis and response genes in rice infected with viruses
(A) Relative expression levels of SA biosynthesis-related genes in mock-treated and RBSDV- and RSV-infected rice plants. (B) The significantly up-regulated pathogenesis-related 1 gene (OsPR-1) in rice caused by virus infection. (C) Relative expression levels of SA response genes in rice plants infected with a virus. All qRT-PCR assays were performed with three biological replicates. The error bars denote the mean ± SD.

## Slide 5
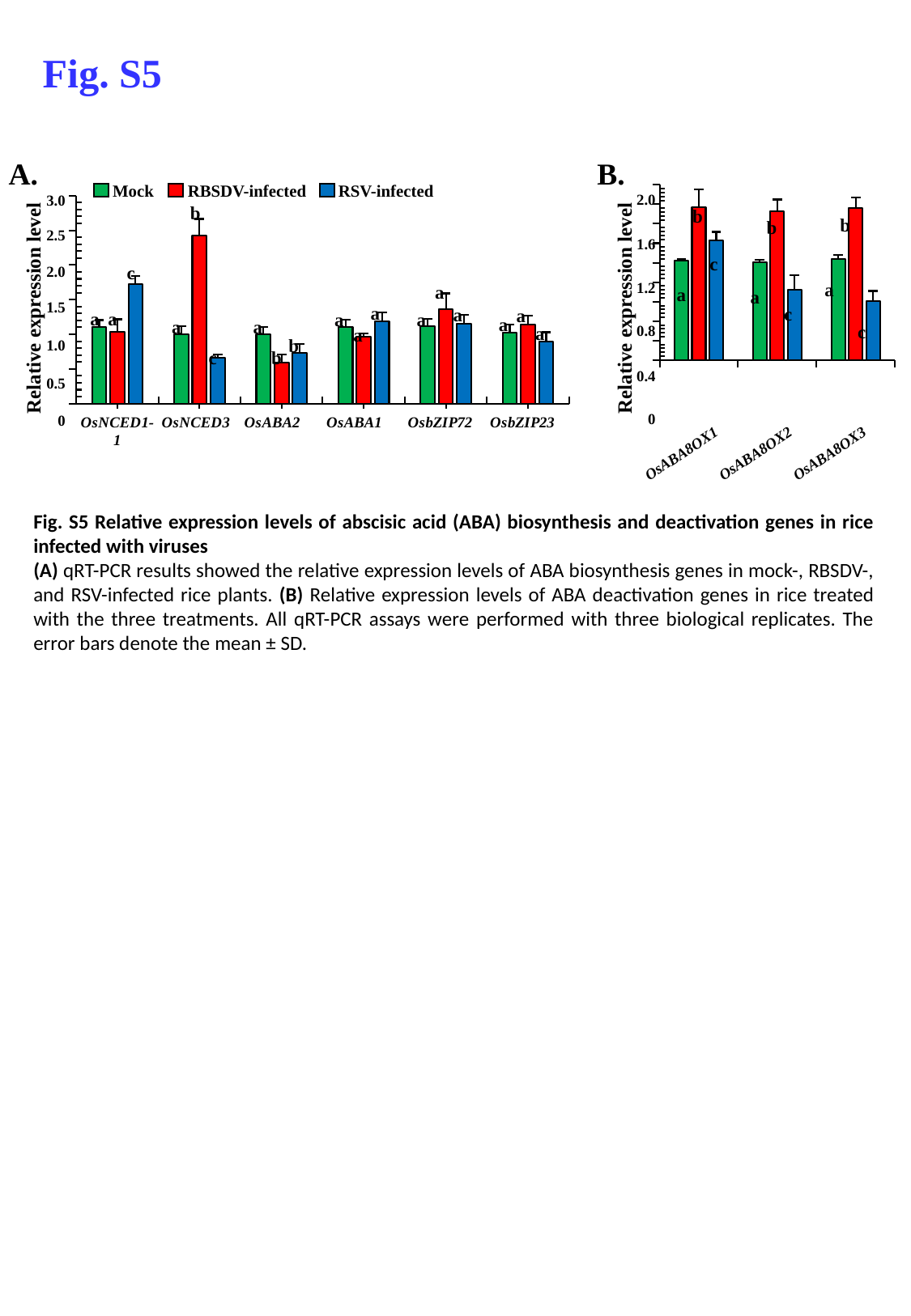

Fig. S5
A.
B.
Mock
RBSDV-infected
RSV-infected
### Chart
| Category | Mock | RBSDV-infection | RSV-infection |
|---|---|---|---|
| OsNCED1-1 | 1.102 | 1.037817167 | 1.722263917 |
| OsNCED3 | 1.002 | 2.419687172 | 0.663045141 |
| OsABA2 | 1.003 | 0.587908511 | 0.726374022 |
| OsABA1 | 1.102 | 0.962175622 | 1.187456071 |
| OsbZIP72 | 1.112 | 1.359326782 | 1.149529048 |
| OsbZIP23 | 1.023 | 1.141059505 | 0.897054874 |
### Chart
| Category | Mock | RBSDV-infected | RSV-infected |
|---|---|---|---|
| OsABA8OX1 | 1.021 | 1.572972381 | 1.231765094 |
| OsABA8OX2 | 1.003 | 1.525448839 | 0.725805583 |
| OsABA8OX3 | 1.041 | 1.564643885 | 0.603153918 |
2.0
3.0
b
b
b
b
2.5
1.6
c
c
2.0
1.2
a
a
a
a
Relative expression level
Relative expression level
1.5
a
c
a
a
a
a
a
a
a
a
a
c
0.8
a
a
b
1.0
c
b
0.4
0.5
0
0
OsABA8OX1
OsABA8OX2
OsABA8OX3
Fig. S5 Relative expression levels of abscisic acid (ABA) biosynthesis and deactivation genes in rice infected with viruses
(A) qRT-PCR results showed the relative expression levels of ABA biosynthesis genes in mock-, RBSDV-, and RSV-infected rice plants. (B) Relative expression levels of ABA deactivation genes in rice treated with the three treatments. All qRT-PCR assays were performed with three biological replicates. The error bars denote the mean ± SD.

## Slide 6
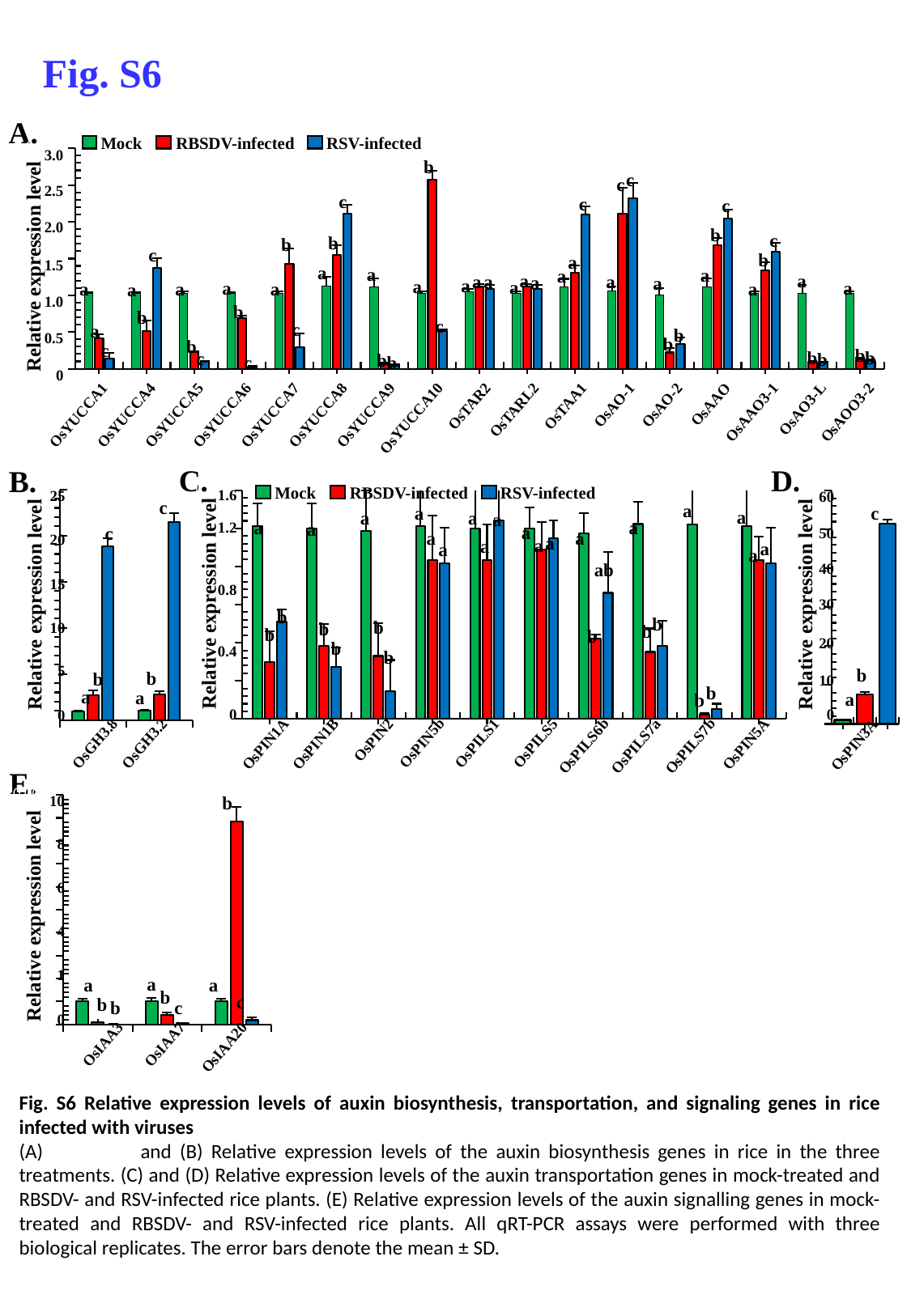

Fig. S6
A.
Mock
RBSDV-infected
RSV-infected
### Chart
| Category | Mock | RBSDV-infected | RSV-infected |
|---|---|---|---|
| OsYUCCA1 | 1.021 | 0.415077999 | 0.136312367 |
| OsYUCCA4 | 1.021 | 0.515604389 | 1.37086487 |
| OsYUCCA5 | 1.025 | 0.227889128 | 0.0927943 |
| OsYUCCA6 | 1.023 | 0.692578623 | 0.023056097 |
| OsYUCCA7 | 1.024 | 1.425673627 | 0.292361985 |
| OsYUCCA8 | 1.123 | 1.553934672 | 2.114355815 |
| OsYUCCA9 | 1.114 | 0.064135738 | 0.045335232 |
| OsYUCCA10 | 1.025 | 2.570202474 | 0.513661805 |
| OsTAR2 | 1.045 | 1.122814453 | 1.088344904 |
| OsTARL2 | 1.025 | 1.122814453 | 1.088344904 |
| OsTAA1 | 1.112 | 1.301872839 | 2.094865541 |
| OsAO-1 | 1.058 | 2.113423424 | 2.316438293 |
| OsAO-2 | 0.998 | 0.221080107 | 0.338384756 |
| OsAAO | 1.115 | 1.676404166 | 2.043688218 |
| OsAAO3-1 | 1.025 | 1.340077803 | 1.595551822 |
| OsAO3-L | 1.023 | 0.089300502 | 0.080648034 |
| OsAAO3-2 | 1.025 | 0.128042935 | 0.106317477 |
3.0
b
c
c
2.5
c
c
c
2.0
b
c
b
b
c
b
a
Relative expression level
1.5
a
a
a
a
a
a
a
a
a
a
a
a
a
a
a
a
a
a
a
a
a
1.0
b
b
c
c
a
b
0.5
b
b
c
b
b
b
c
b
b
b
c
0
OsYUCCA1
OsYUCCA4
OsYUCCA5
OsYUCCA6
OsYUCCA7
OsYUCCA8
OsYUCCA9
OsYUCCA10
OsTAR2
OsTARL2
OsTAA1
OsAO-1
OsAO-2
OsAAO
OsAAO3-1
OsAO3-L
OsAOO3-2
C.
D.
B.
### Chart
| Category | | | |
|---|---|---|---|
| OsGH3.8 | 0.954 | 2.702066522 | 18.87125536 |
| OsGH3.2 | 0.995 | 2.7642296 | 21.48011619 |Mock
RBSDV-infected
RSV-infected
### Chart
| Category | Mock | RBSDV-infection | RSV-infection |
|---|---|---|---|
| OsPIN1A | 1.012 | 0.298592983 | 0.511947263 |
| OsPIN1B | 0.998 | 0.385213712 | 0.272434259 |
| OsPIN2 | 0.987 | 0.330533157 | 0.144371305 |
| OsPIN5b | 1.013 | 0.835407673 | 0.818146268 |
| OsPILS1 | 1.001 | 0.832304846 | 1.043866616 |
| OsPILS5 | 0.999 | 0.888507412 | 0.947988719 |
| OsPILS6b | 0.973 | 0.422357381 | 0.661975673 |
| OsPILS7a | 1.023 | 0.351783374 | 0.384147259 |
| OsPILS7b | 1.021 | 0.021662814 | 0.050806185 |
| OsPIN5A | 1.013 | 0.835407673 | 0.818146268 |
### Chart
| Category | OsPIN3A |
|---|---|1.6
25
60
c
a
a
c
a
a
a
a
a
a
1.2
a
a
c
50
a
a
20
a
a
a
a
a
a
ab
40
15
0.8
Relative expression level
Relative expression level
Relative expression level
30
b
b
b
b
10
b
b
b
20
b
0.4
b
5
b
b
b
10
b
a
a
a
b
0
0
0
OsGH3.8
OsGH3.2
OsPIN1A
OsPIN1B
OsPIN2
OsPIN5b
OsPILS1
OsPILS5
OsPILS6b
OsPILS7a
OsPILS7b
OsPIN5A
OsPIN3A
E.
### Chart
| Category | Mock | RBSDV-infected | RSV-infected |
|---|---|---|---|
| OsIAA3 | 1.012 | 0.083276237 | 0.002475333 |
| OsIAA7 | 1.001 | 0.418434221 | 0.059173558 |
| OsIAA20 | 0.998 | 8.829922616 | 0.184924411 |10
b
8
6
Relative expression level
4
1
a
a
a
b
c
b
c
b
0
OsIAA3
OsIAA7
OsIAA20
Fig. S6 Relative expression levels of auxin biosynthesis, transportation, and signaling genes in rice infected with viruses
(A)	and (B) Relative expression levels of the auxin biosynthesis genes in rice in the three treatments. (C) and (D) Relative expression levels of the auxin transportation genes in mock-treated and RBSDV- and RSV-infected rice plants. (E) Relative expression levels of the auxin signalling genes in mock-treated and RBSDV- and RSV-infected rice plants. All qRT-PCR assays were performed with three biological replicates. The error bars denote the mean ± SD.

## Slide 7
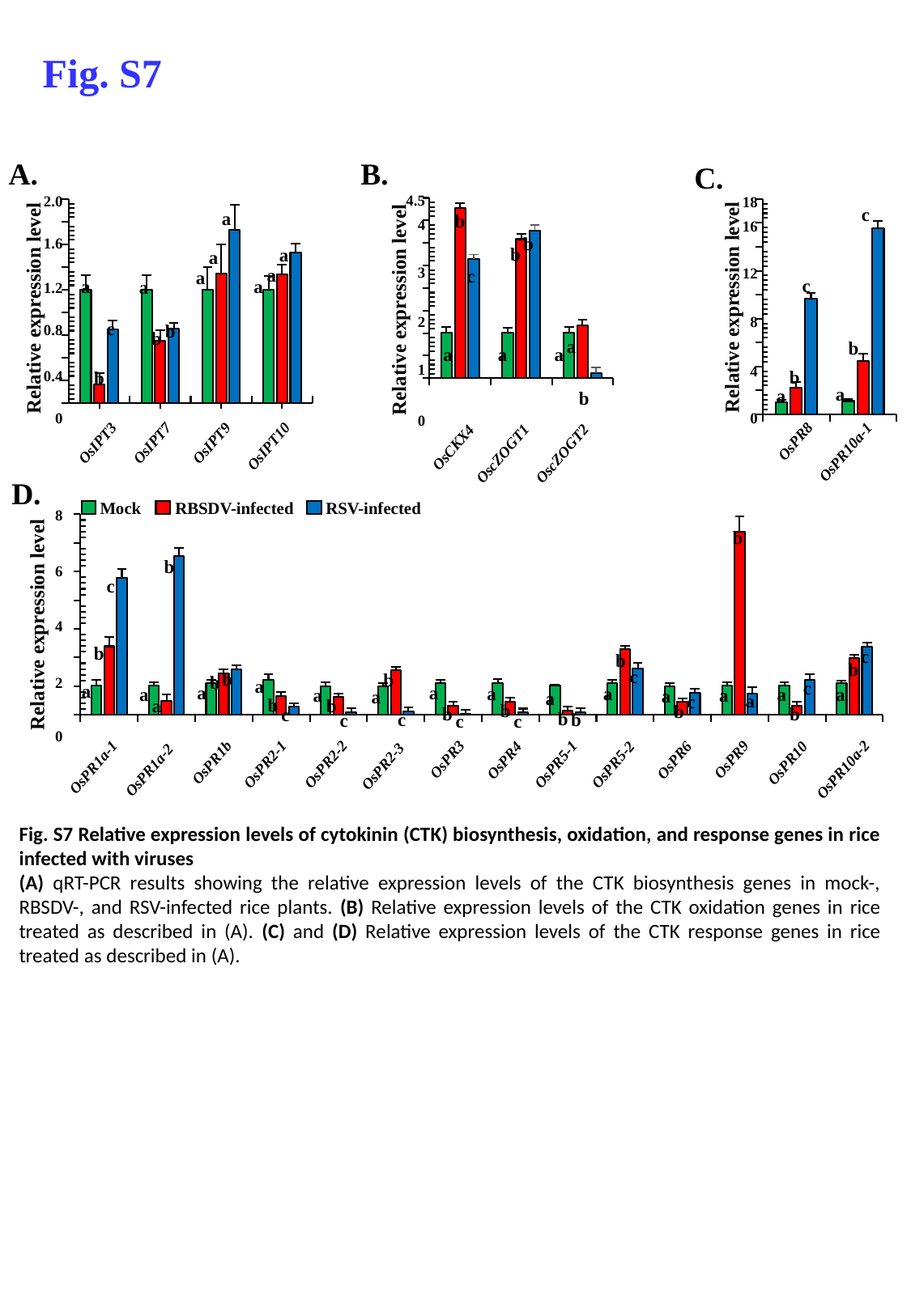

Fig. S7
A.
B.
C.
### Chart
| Category | Mock | RBSDV-infected | RSV-infected |
|---|---|---|---|
| OsCKX4 | 1.012 | 3.780743616 | 2.640139407 |
| OscZOGT1 | 1.011 | 3.083790701 | 3.279598289 |
| OscZOGT2 | 1.009 | 1.175879644 | 0.115670718 |
### Chart
| Category | Mock | RBSDV-infected | RSV-infected |
|---|---|---|---|
| OsIPT3 | 1.001 | 0.162244355 | 0.652872059 |
| OsIPT7 | 1.002 | 0.553116126 | 0.655101719 |
| OsIPT9 | 0.998 | 1.144936956 | 1.526769861 |
| OsIPT10 | 0.999 | 1.137095315 | 1.327305879 |
### Chart
| Category | | | |
|---|---|---|---|
| OsPR8 | 0.987 | 2.241692019 | 9.643070639 |
| OsPR10a-1 | 1.112 | 4.469076841 | 15.55335077 |4.5
2.0
18
c
a
b
4
16
b
1.6
b
a
a
3
a
12
c
a
c
a
a
a
1.2
Relative expression level
Relative expression level
Relative expression level
2
8
c
b
0.8
b
a
b
a
a
a
1
4
b
b
0.4
a
a
b
0
0
0
OsIPT3
OsIPT7
OsIPT9
OsIPT10
OsPR8
OsPR10a-1
OsCKX4
OscZOGT1
OscZOGT2
D.
Mock
RBSDV-infected
RSV-infected
### Chart
| Category | Mock | RBSDV-infected | RSV-infected |
|---|---|---|---|
| OsPR1a-1 | 1.021 | 2.393789141 | 4.772678631 |
| OsPR1a-2 | 1.013 | 0.480295642 | 5.556716041 |
| OsPR1b | 1.112 | 1.453947897 | 1.582857499 |
| OsPR2-1 | 1.213 | 0.649133496 | 0.283114823 |
| OsPR2-2 | 1.005 | 0.629378445 | 0.097071963 |
| OsPR2-3 | 0.998 | 1.568058933 | 0.121775292 |
| OsPR3 | 1.115 | 0.322255161 | 0.045715596 |
| OsPR4 | 1.115 | 0.466473664 | 0.095438754 |
| OsPR5-1 | 1.021 | 0.159399999 | 0.098443501 |
| OsPR5-2 | 1.112 | 2.282653194 | 1.61191995 |
| OsPR6 | 0.998 | 0.451958735 | 0.776200605 |
| OsPR9 | 1.023 | 6.396313737 | 0.736769324 |
| OsPR10 | 1.025 | 0.330017229 | 1.214546261 |
| OsPR10a-2 | 1.097 | 1.973149182 | 2.387642124 |8
b
b
6
c
Relative expression level
4
b
c
b
b
c
b
b
b
2
a
c
a
a
a
a
a
a
a
a
a
a
a
a
a
a
c
b
b
a
b
b
b
b
c
b
c
b
c
c
c
0
OsPR10a-2
OsPR1a-1
OsPR1b
OsPR2-1
OsPR2-2
OsPR5-1
OsPR9
OsPR3
OsPR5-2
OsPR10
OsPR6
OsPR4
OsPR2-3
OsPR1a-2
Fig. S7 Relative expression levels of cytokinin (CTK) biosynthesis, oxidation, and response genes in rice infected with viruses
(A) qRT-PCR results showing the relative expression levels of the CTK biosynthesis genes in mock-, RBSDV-, and RSV-infected rice plants. (B) Relative expression levels of the CTK oxidation genes in rice treated as described in (A). (C) and (D) Relative expression levels of the CTK response genes in rice treated as described in (A).

## Slide 8
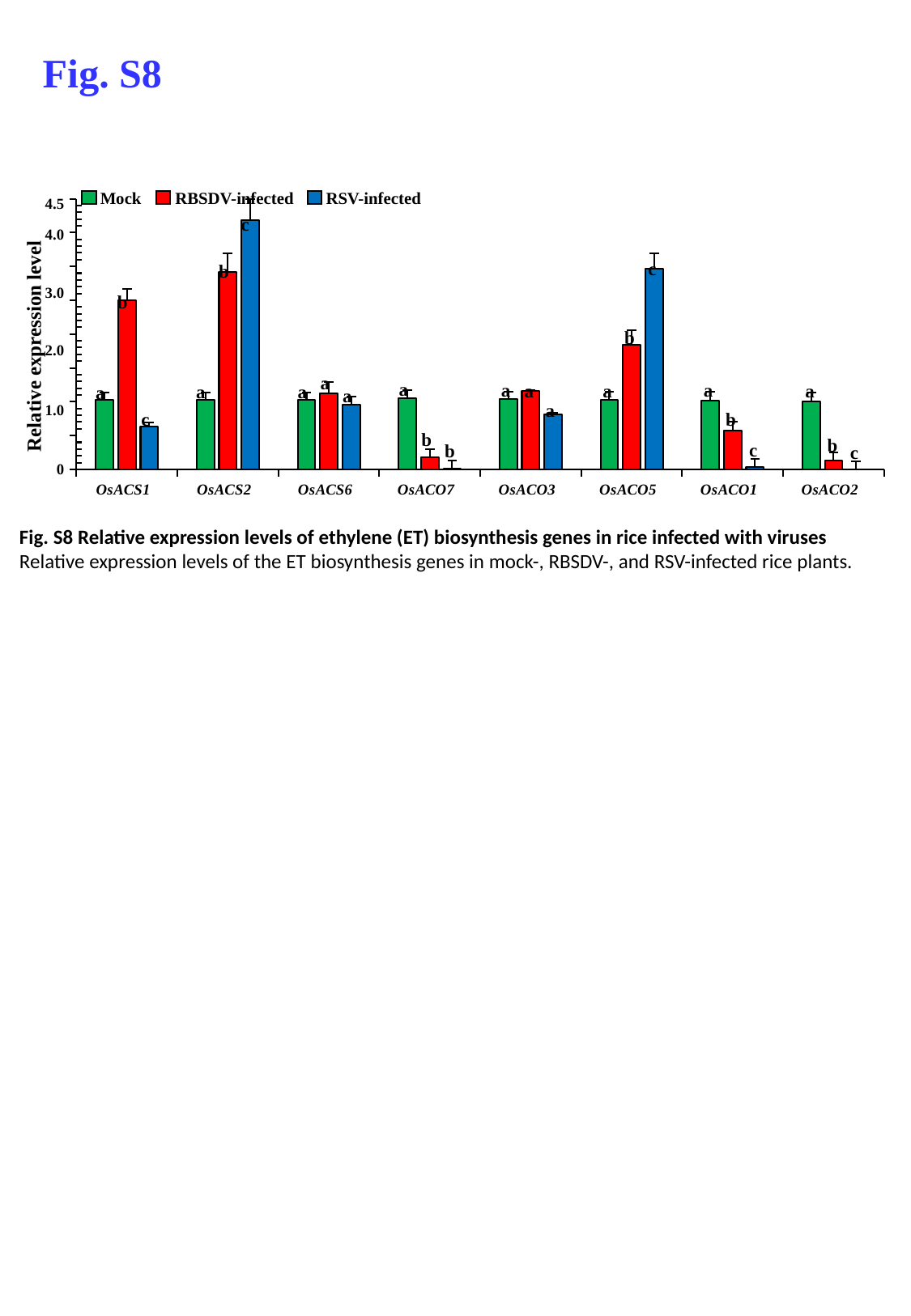

Fig. S8
Mock
RBSDV-infected
RSV-infected
### Chart
| Category | Mock | RBSDV-infected | RSV-infected |
|---|---|---|---|
| OsACS1 | 1.023 | 2.498330818 | 0.633236396 |
| OsACS2 | 1.025 | 2.917719267 | 3.68664762 |
| OsACS6 | 1.032 | 1.11831618 | 0.960793689 |
| OsACO7 | 1.054 | 0.17753446 | 0.011875055 |
| OsACO3 | 1.045 | 1.153973111 | 0.815946303 |
| OsACO5 | 1.032 | 1.837023151 | 2.967380925 |
| OsACO1 | 1.014 | 0.577960089 | 0.030267351 |
| OsACO2 | 1.009 | 0.128775549 | 0.002581206 |4.5
c
4.0
c
b
3.0
b
b
Relative expression level
2.0
a
a
a
a
a
a
a
a
a
a
a
a
1.0
b
c
b
b
c
b
c
0
Fig. S8 Relative expression levels of ethylene (ET) biosynthesis genes in rice infected with viruses
Relative expression levels of the ET biosynthesis genes in mock-, RBSDV-, and RSV-infected rice plants.

## Slide 9
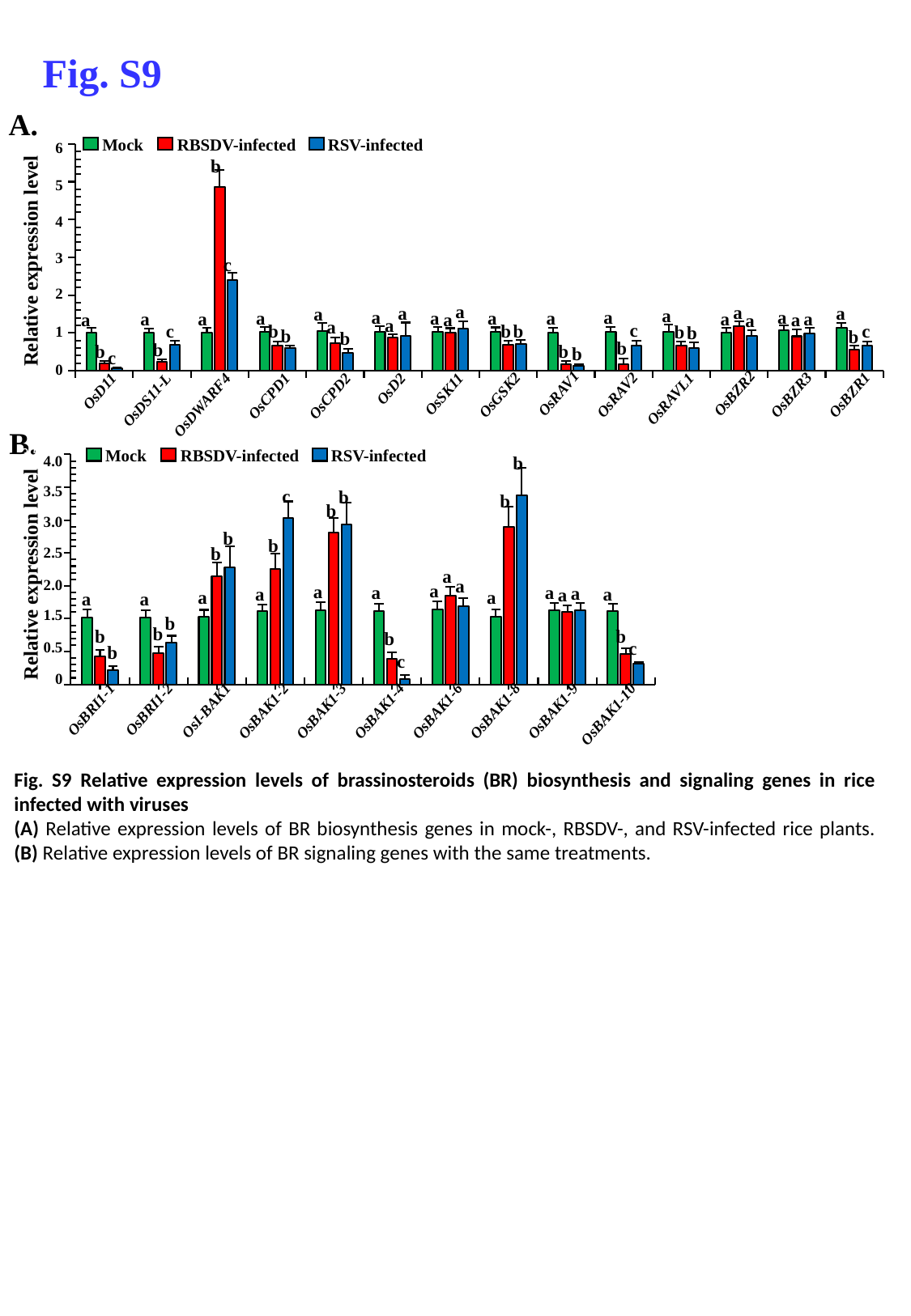

Fig. S9
A.
Mock
RBSDV-infected
RSV-infected
### Chart
| Category | Mock | RBSDV-infected | RSV-infected |
|---|---|---|---|
| OsD11 | 1.002 | 0.196972596 | 0.054221227 |
| OsD11-L | 1.001 | 0.229469323 | 0.685543378 |
| OsDWARF4 | 0.998 | 4.862159754 | 2.393165739 |
| OsCPD1 | 1.023 | 0.671962483 | 0.605977182 |
| OsCPD2 | 1.056 | 0.728218274 | 0.46747131 |
| OsD2 | 1.032 | 0.867406503 | 0.924460314 |
| OsSK11 | 1.022 | 1.000810889 | 1.105200048 |
| OsGSK2 | 1.021 | 0.674862106 | 0.700171902 |
| OsRAV1 | 1.012 | 0.160936533 | 0.123 |
| OsRAV2 | 1.022 | 0.163950106 | 0.666728333 |
| OsRAVL1 | 1.032 | 0.670928781 | 0.592473849 |
| OsBZR2 | 1.015 | 1.176475803 | 0.914190207 |
| OsBZR3 | 1.066 | 0.908934268 | 0.993726768 |
| OsBZR1 | 1.143 | 0.554150358 | 0.654497646 |6
b
5
4
3
Relative expression level
c
2
a
a
a
a
a
a
a
a
a
a
a
a
a
a
a
a
a
a
a
a
a
a
a
c
c
b
b
b
c
b
b
1
b
b
b
b
b
b
b
b
c
0
OsBZR2
OsRAV1
OsGSK2
OsBZR1
OsRAV2
OsBZR3
OsSK11
OsD11
OsDS11-L
OsDWARF4
OsCPD1
OsCPD2
OsD2
OsRAVL1
B.
Mock
RBSDV-infected
RSV-infected
### Chart
| Category | Mock | RBSDV-infected | RSV-infected |
|---|---|---|---|
| OsBRI1-1 | 1.021 | 0.431506394 | 0.219312965 |
| OsBRI1-2 | 1.014 | 0.475048769 | 0.635729457 |
| OsI-BAK1 | 1.032 | 1.645465399 | 1.776875369 |
| OsBAK1-2 | 1.115 | 1.756918068 | 2.534422179 |
| OsBAK1-3 | 1.124 | 2.314927226 | 2.432227141 |
| OsBAK1-4 | 1.112 | 0.386757213 | 0.085645445 |
| OsBAK1-6 | 1.145 | 1.34618341 | 1.188864978 |
| OsBAK1-8 | 1.028 | 2.397646175 | 2.873609262 |
| OsBAK1-9 | 1.132 | 1.106150115 | 1.129374335 |
| OsBAK1-10 | 1.112 | 0.45789019 | 0.30996944 |4.0
b
3.5
c
b
b
b
3.0
b
b
b
2.5
Relative expression level
a
a
2.0
a
a
a
a
a
a
a
a
a
a
a
a
1.5
b
b
b
b
b
c
0.5
b
c
0
OsBRI1-1
OsBRI1-2
OsI-BAK1
OsBAK1-2
OsBAK1-3
OsBAK1-4
OsBAK1-6
OsBAK1-8
OsBAK1-9
OsBAK1-10
Fig. S9 Relative expression levels of brassinosteroids (BR) biosynthesis and signaling genes in rice infected with viruses
(A) Relative expression levels of BR biosynthesis genes in mock-, RBSDV-, and RSV-infected rice plants. (B) Relative expression levels of BR signaling genes with the same treatments.

## Slide 10
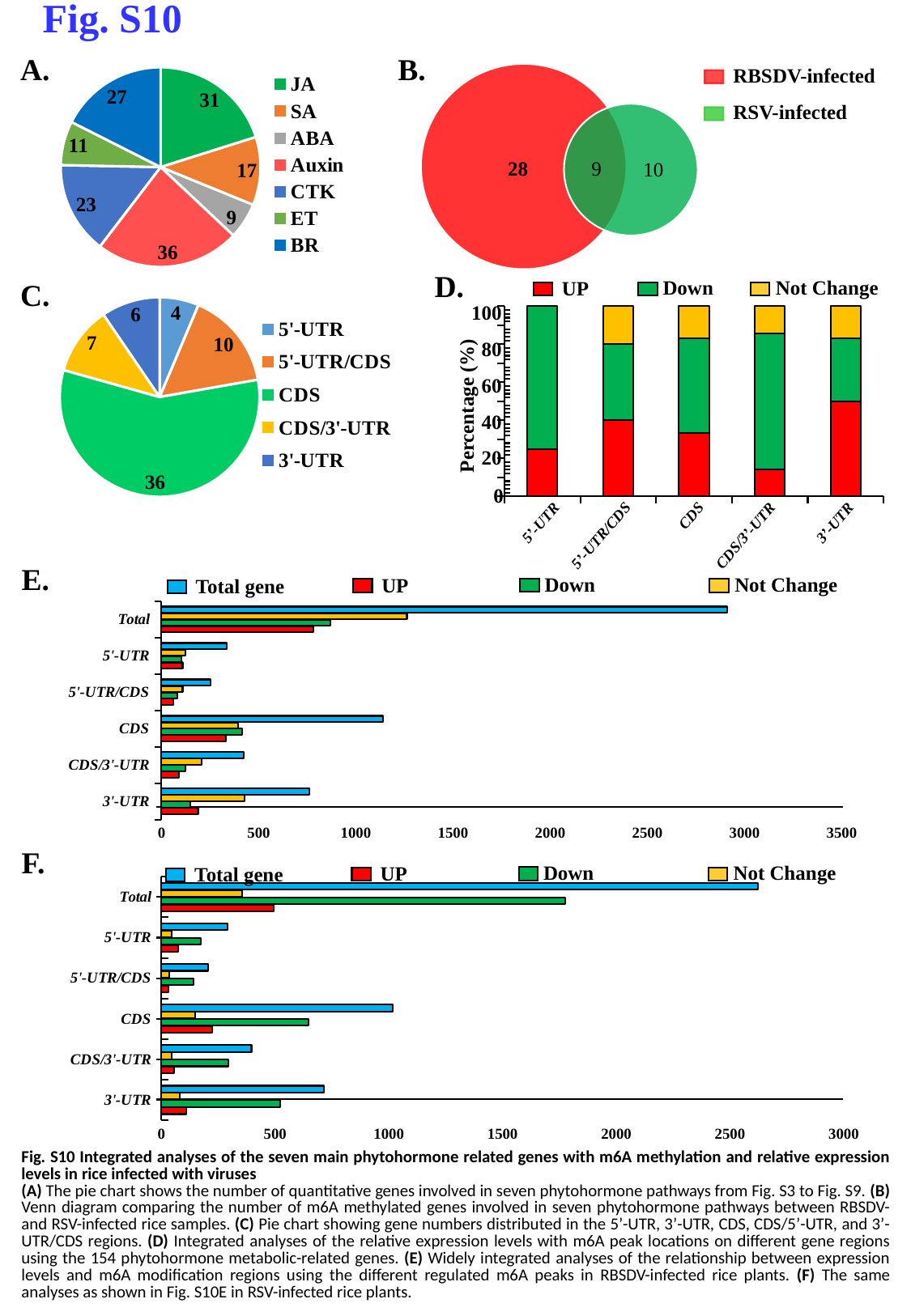

Fig. S10
A.
### Chart
| Category | |
|---|---|
| JA | 31.0 |
| SA | 17.0 |
| ABA | 9.0 |
| Auxin | 36.0 |
| CTK | 23.0 |
| ET | 11.0 |
| BR | 27.0 |B.
RBSDV-infected
RSV-infected
28
9
10
D.
### Chart
| Category | |
|---|---|
| 5'-UTR | 4.0 |
| 5'-UTR/CDS | 10.0 |
| CDS | 36.0 |
| CDS/3'-UTR | 7.0 |
| 3'-UTR | 6.0 |Down
Not Change
UP
C.
100
### Chart
| Category | Up-regulated | Down-regulated | Not change |
|---|---|---|---|
| 5'-UTR | 0.25 | 0.75 | 0.0 |
| 5'-UTR/CDS | 0.4 | 0.4 | 0.2 |
| CDS | 0.333 | 0.5 | 0.167 |
| CDS/3'-UTR | 0.143 | 0.714 | 0.143 |
| 3'-UTR | 0.5 | 0.333 | 0.17 |80
60
Percentage (%)
40
20
0
CDS
3’-UTR
5’-UTR
CDS/3’-UTR
5’-UTR/CDS
E.
Down
Not Change
UP
Total gene
### Chart
| Category | 上调（Up） | 下调（Down） | 不变（Unchanged） | |
|---|---|---|---|---|
| 3'-UTR | 188.0 | 147.0 | 426.0 | 761.0 |
| CDS/3'-UTR | 91.0 | 124.0 | 208.0 | 423.0 |
| CDS | 330.0 | 414.0 | 395.0 | 1139.0 |
| 5'-UTR/CDS | 62.0 | 83.0 | 109.0 | 254.0 |
| 5'-UTR | 109.0 | 102.0 | 125.0 | 336.0 |
| Total | 780.0 | 870.0 | 1263.0 | 2913.0 |F.
Down
Not Change
UP
Total gene
### Chart
| Category | 上调（Up） | 下调（Down） | 不变（Unchanged） | |
|---|---|---|---|---|
| 3'-UTR | 109.0 | 523.0 | 82.0 | 714.0 |
| CDS/3'-UTR | 57.0 | 293.0 | 46.0 | 396.0 |
| CDS | 222.0 | 646.0 | 149.0 | 1017.0 |
| 5'-UTR/CDS | 32.0 | 141.0 | 34.0 | 207.0 |
| 5'-UTR | 74.0 | 172.0 | 44.0 | 290.0 |
| Total | 494.0 | 1775.0 | 355.0 | 2624.0 |Fig. S10 Integrated analyses of the seven main phytohormone related genes with m6A methylation and relative expression levels in rice infected with viruses
(A) The pie chart shows the number of quantitative genes involved in seven phytohormone pathways from Fig. S3 to Fig. S9. (B) Venn diagram comparing the number of m6A methylated genes involved in seven phytohormone pathways between RBSDV- and RSV-infected rice samples. (C) Pie chart showing gene numbers distributed in the 5’-UTR, 3’-UTR, CDS, CDS/5’-UTR, and 3’-UTR/CDS regions. (D) Integrated analyses of the relative expression levels with m6A peak locations on different gene regions using the 154 phytohormone metabolic-related genes. (E) Widely integrated analyses of the relationship between expression levels and m6A modification regions using the different regulated m6A peaks in RBSDV-infected rice plants. (F) The same analyses as shown in Fig. S10E in RSV-infected rice plants.

## Slide 11
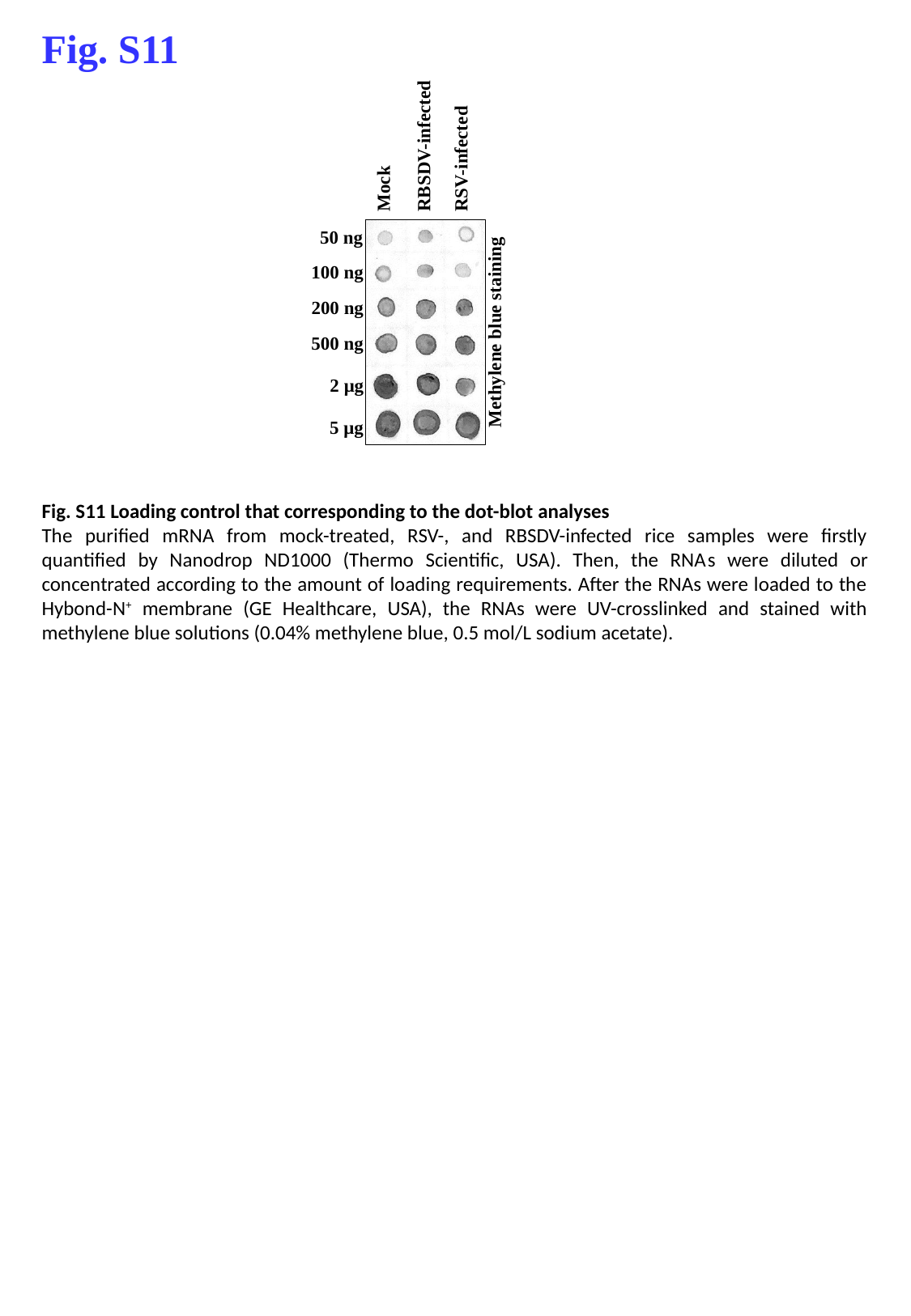

Fig. S11
RBSDV-infected
RSV-infected
Mock
50 ng
100 ng
200 ng
500 ng
2 μg
5 μg
Methylene blue staining
Fig. S11 Loading control that corresponding to the dot-blot analyses
The purified mRNA from mock-treated, RSV-, and RBSDV-infected rice samples were firstly quantified by Nanodrop ND1000 (Thermo Scientific, USA). Then, the RNAs were diluted or concentrated according to the amount of loading requirements. After the RNAs were loaded to the Hybond-N+ membrane (GE Healthcare, USA), the RNAs were UV-crosslinked and stained with methylene blue solutions (0.04% methylene blue, 0.5 mol/L sodium acetate).

## Slide 12
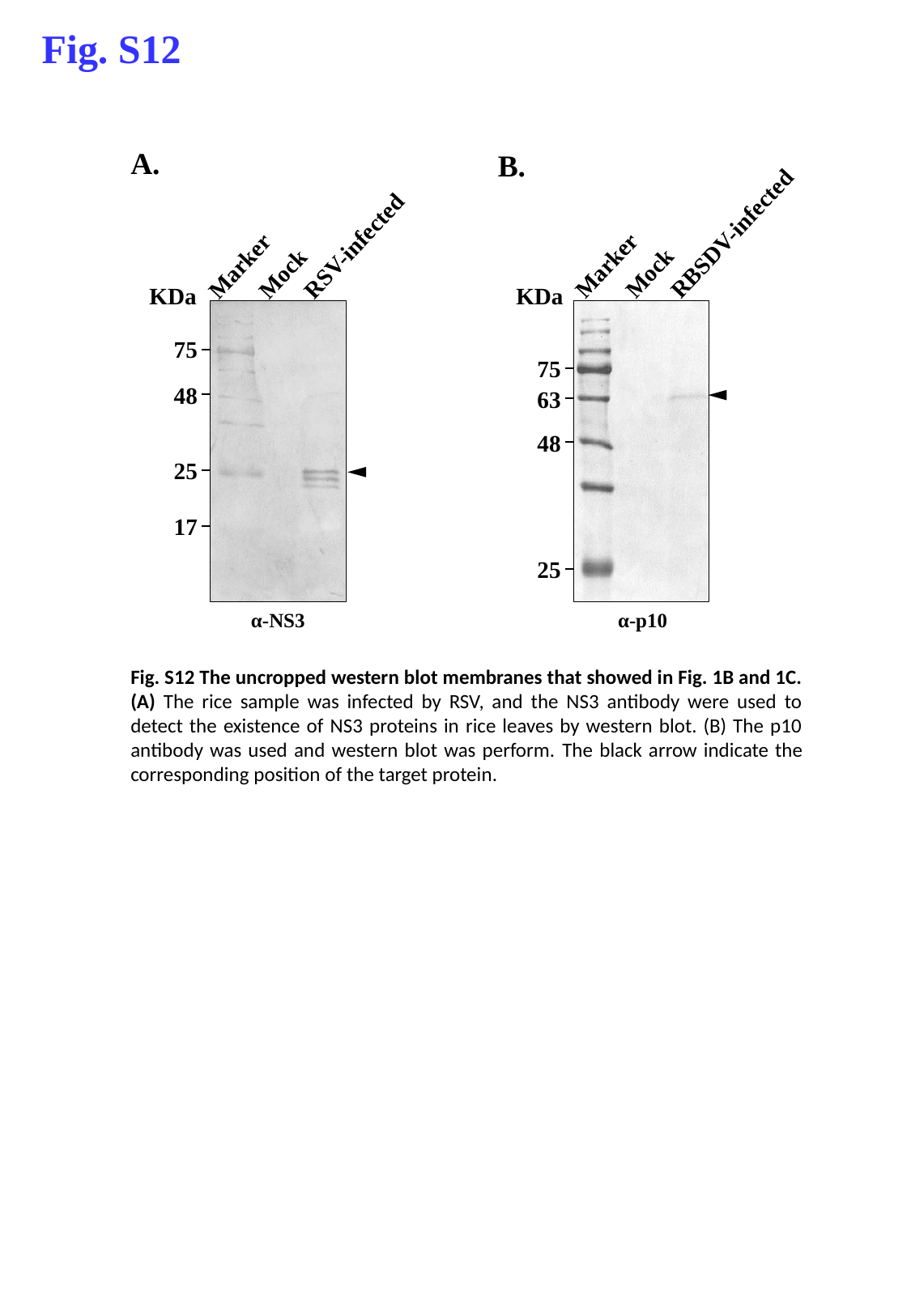

Fig. S12
A.
B.
RBSDV-infected
RSV-infected
Marker
Mock
Marker
Mock
KDa
KDa
75
75
48
63
48
25
17
25
α-p10
α-NS3
Fig. S12 The uncropped western blot membranes that showed in Fig. 1B and 1C. (A) The rice sample was infected by RSV, and the NS3 antibody were used to detect the existence of NS3 proteins in rice leaves by western blot. (B) The p10 antibody was used and western blot was perform. The black arrow indicate the corresponding position of the target protein.
