# Supplementary material for: The dynamics of N6-methyladenine RNA modification in interactions between rice and plant viruses
Source: Genome Biol. 2021 Jun 24;22:189. doi: 10.1186/s13059-021-02410-2 (PMC8229379; doi:10.1186/s13059-021-02410-2)
Supplement: Supplementary file 2 — Additional file 2: Table S1. Primers used for RSV and RBSDV detections in this study. Table S2. Sequenced and rice genome mapped reads in m6A-IP-seq, input RNA-seq rice samples. Table S3. Nucleotide localization and enrichment of the top 10 m6A peaks identified in Mock-, RSV-, and RBSDV-infected rice transcripts by m6A-IP-Seq. Table S4. The category of the differential m6A peaks upon two viruses' infection in rice. Table S5. Nucleotide localization and enrichment of the m6A peaks identified in RSV and RBSDV genomics by m6A-IP-Seq. Table S6. Gene ID and their fpkm analyses. Table S7. Different peaks statistic. Table S8. The m6A peaks appeared in different treatments. Table S9. The most abundant consensus motif in Mock, RSV-, and RBSDV-infected rice plant using suite of DREME and MEME. Table S10. Analyses of the m6A peaks that containing most four common consensus appeared in other species. Table S11. Detail information of ListHits Gene and m6A methylated genes under rice viruses’ infection derived from Additional file 2: Table S16. Table S12. Integrated analyses of the m6A methylation related genes with m6A modifications and expression profiles. Table S13. Integrated analyses of the anti-viral RNA silencing pathway related genes with m6A modification and expression profile. Table S14. Integrated analyses of the plant hormone metabolic genes with m6A modifications and expression profiles. Table S15. Integrated analyses of the relationship betwwen relative expression and m6A positions. Table S16. Summary of the m6A RNA methylation level in enriched top 5 KEGG pathways related genes from RNA-seq under rice viruses’ infection. Table S17. Detail information of the common m6A methylated genes appeared in enriched top 5 KEGG pathways under rice viruses' infection. Table S18. Primers used for qRT-PCR validation of the methylation of OsAGO18 and OsSLRL1 genes. Table S19. Primers used in qRT-PCR qualification of the candidate genes. [file 13059_2021_2410_MOESM2_ESM.zip › Table S11.docx]

**Table S11:** Detail information of ListHits Gene and m6A methylated genes under rice viruses’ infection derived from Supplementary Table S15

**Common ListHits Gene (RBSDV & RSV):**

**ko00940 (97):**

Os02g0236800 Os10g0109600 Os05g0427400 Os08g0509200 Os01g0378100 Os03g0234900 Os06g0165800 Os04g0474500 Os03g0212800 Os01g0962700 Os11g0661600 Os05g0494000 Os01g0326000 Os08g0113000 Os12g0191500 Os02g0177600 Os08g0543400 Os12g0530100 Os09g0262000 Os07g0115300 Os06g0592400 Os02g0187800 Os02g0240300 Os06g0656500 Os09g0127300 Os02g0236600 Os08g0509400 Os04g0518100 Os04g0498700 Os07g0104100 Os02g0812000 Os11g0210100 Os09g0491100 Os06g0683300 Os03g0749500 Os04g0518400 Os01g0205900 Os01g0543100 Os05g0320700 Os07g0156200 Os06g0306300 Os02g0697400 Os02g0161800 Os03g0703000 Os01g0813700 Os01g0327400 Os03g0121200 Os01g0283600 Os07g0677500 Os07g0471300 Os04g0229100 Os08g0205000 Os02g0626400 Os04g0474900 Os02g0626600 Os06g0548100 Os04g0500700 Os04g0656800 Os03g0339300 Os04g0688300 Os06g0695500 Os08g0441500 Os05g0499400 Os07g0677600 Os05g0499300 Os06g0274800 Os02g0627100 Os10g0109300 Os01g0508000 Os02g0611800 Os03g0434800 Os06g0681600 Os09g0511900 Os10g0512400 Os02g0833900 Os05g0135500 Os04g0513100 Os07g0677100 Os12g0112000 Os02g0626100 Os08g0245200 Os07g0694300 Os01g0294700 Os10g0536700 Os02g0180700 Os03g0703100 Os02g0811600 Os01g0263300 Os10g0323500 Os09g0507500 Os01g0327100 Os08g0277200 Os03g0368900 Os11g0112400 Os07g0656200 Os03g0749300 Os04g0474700

**ko04075 (70):**

Os01g0646300 Os09g0459500 Os05g0572700 Os06g0137400 Os06g0335500 Os02g0787300 Os07g0592600 Os01g0764800 Os04g0456900 Os04g0432000 Os02g0769100 Os01g0190300 Os07g0671500 Os11g0141900 Os06g0696400 Os10g0571300 Os11g0143300 Os01g0231000 Os09g0437400 Os02g0445600 Os03g0782500 Os10g0392400 Os03g0707600 Os03g0402800 Os09g0325700 Os05g0563400 Os03g0633800 Os01g0221100 Os08g0474500 Os09g0439200 Os05g0186100 Os12g0601300 Os04g0662400 Os01g0785400 Os06g0211200 Os10g0147400 Os01g0178500 Os06g0696600 Os05g0230700 Os01g0670800 Os12g0626200 Os10g0391400 Os04g0395800 Os08g0508700 Os03g0181100 Os05g0523300 Os07g0182400 Os03g0180800 Os11g0169200 Os02g0445100 Os03g0797800 Os04g0617050 Os12g0601400 Os05g0178600 Os04g0662200 Os06g0183100 Os01g0194300 Os03g0180900 Os12g0138500 Os02g0228900 Os06g0597000 Os07g0155600 Os11g0221000 Os03g0742900 Os07g0129200 Os01g0869900 Os02g0557800 Os07g0615200 Os07g0580500 Os01g0718300

**ko00520 (47):**

Os01g0891000 Os08g0526100 Os08g0129700 Os07g0139400 Os04g0640700 Os12g0443500 Os05g0415700 Os06g0356800 Os04g0493400 Os01g0174300 Os07g0674100 Os01g0296700 Os03g0268400 Os05g0580000 Os11g0131900 Os09g0504000 Os07g0604800 Os01g0837300 Os09g0531900 Os02g0605900 Os11g0701600 Os03g0330000 Os10g0543400 Os06g0712500 Os04g0494100 Os01g0969100 Os04g0608100 Os03g0757900 Os12g0128700 Os06g0227250 Os01g0660200 Os01g0814900 Os01g0860500 Os07g0632000 Os08g0327100 Os03g0132900 Os05g0399400 Os12g0443600 Os07g0686900 Os08g0113100 Os09g0480400 Os05g0115900 Os04g0376400 Os11g0462100 Os02g0791500 Os07g0681700 Os03g0278000

**ko00500 (41):**

Os01g0508000 Os01g0813700 Os02g0139300 Os02g0661100 Os02g0733300 Os02g0753000 Os03g0141200 Os03g0212800 Os03g0386500 Os03g0401300 Os03g0703000 Os03g0703100 Os03g0736300 Os03g0749300 Os03g0749500 Os04g0413500 Os04g0474500 Os04g0474700 Os04g0474900 Os04g0497200 Os04g0513100 Os05g0580000 Os06g0194900 Os06g0256900 Os06g0675700 Os06g0683300 Os07g0510200 Os07g0543100 Os07g0543300 Os07g0627000 Os07g0656200 Os08g0113100 Os08g0224500 Os08g0414700 Os08g0509200 Os08g0509400 Os09g0397300 Os09g0469400 Os09g0491100 Os09g0511900 Os09g0530200

**ko00270 (37):**

Os01g0290600 Os01g0293000 Os01g0323600 Os01g0580500 Os01g0649100 Os01g0814800 Os01g0829800 Os02g0222100 Os02g0302700 Os02g0611200 Os03g0196600 Os03g0221200 Os03g0231600 Os03g0338000 Os03g0747800 Os03g0760700 Os03g0850400 Os04g0578000 Os05g0135700 Os05g0149400 Os05g0475400 Os06g0149700 Os06g0345200 Os06g0564700 Os08g0342400 Os08g0434300 Os09g0294000 Os09g0424300 Os09g0451000 Os09g0451400 Os10g0104900 Os10g0419500 Os11g0455500 Os11g0552000 Os12g0263000 Os12g0623900 Os12g0624000

**m6A Methylated Gene No.**

- **ko00940 (RBSDV-infected): 15**

Os01g0205900 Os01g0327100 Os01g0508000 Os01g0962700 Os01g0813700 Os02g0161800 Os02g0626100 Os03g0339300 Os07g0471300 Os03g0703000 Os03g0703100 Os04g0498700 Os04g0688300 Os08g0205000 Os08g0509400

**ko00940 (RSV-infected): 13**

Os01g0327100 Os01g0508000 Os01g0962700 Os01g0294700 Os02g0626100 Os03g0339300 Os07g0471300 Os03g0434800 Os03g0703100 Os04g0513100 Os04g0656800 Os06g0681600 Os07g0656200

**ko00940 Common**:

Os01g0327100 Os01g0508000 Os01g0962700 Os02g0626100 Os03g0339300 Os03g0762400 Os03g0703100

- **ko04075 (RBSDV-infected): 8**

Os01g0785400 Os01g0718300 Os01g0646300 Os02g0787300 Os05g0230700 Os05g0186100 Os10g0147400 Os11g0221000

**ko04075 (RSV-infected): 11**

Os01g0785400 Os01g0646300 Os01g0178500 Os02g0787300 Os05g0563400 Os07g0671500 Os07g0592600 Os08g0508700 Os11g0221000 Os11g0141900 Os12g0601300

**ko04075 Common: 4**

Os01g0785400 Os01g0646300 Os02g0787300 Os11g0221000

- **ko00520** **(RBSDV-infected): 2**

Os11g0131900 Os03g0330000

**ko00520** **(RSV-infected): 4**

Os09g0504000 Os08g0526100 Os04g0608100 Os01g0969100

- **ko00500 (RBSDV-infected): 11**

Os01g0813700 Os01g0508000 Os02g0661100 Os02g0139300 Os03g0703000 Os04g0413500 Os06g0675700 Os06g0256900 Os07g0627000 Os08g0509400 Os08g0224500

**ko00500 (RSV-infected): 15**

Os01g0508000 Os02g0753000 Os02g0733300 Os02g0661100 Os02g0139300 Os03g0703100 Os04g0513100 Os06g0675700 Os06g0256900 Os07g0656200 Os07g0510200 Os08g0414700 Os08g0224500 Os08g0414700 Os09g0469400

**ko00500 Common: 8**

Os01g0508000 Os02g0661100 Os02g0139300 Os03g0703100 Os06g0675700 Os06g0256900 Os07g0656200 Os08g0224500

- **ko00270 (RBSDV-infected):9**

Os01g0814800 Os01g0323600 Os01g0290600 Os03g0338000 Os03g0221200 Os04g0578000 Os06g0345200 Os08g0342400 Os10g0419500

**ko00270 (RSV-infected):4**

Os01g0323600 Os09g0294000 Os10g0419500 Os10g0104900

**ko00270 Common: 2**

Os01g0323600 Os10g0419500
