# Supplementary material for: The dynamics of N6-methyladenine RNA modification in interactions between rice and plant viruses
Source: Genome Biol. 2021 Jun 24;22:189. doi: 10.1186/s13059-021-02410-2 (PMC8229379; doi:10.1186/s13059-021-02410-2)
Supplement: Supplementary file 2 — Additional file 2: Table S1. Primers used for RSV and RBSDV detections in this study. Table S2. Sequenced and rice genome mapped reads in m6A-IP-seq, input RNA-seq rice samples. Table S3. Nucleotide localization and enrichment of the top 10 m6A peaks identified in Mock-, RSV-, and RBSDV-infected rice transcripts by m6A-IP-Seq. Table S4. The category of the differential m6A peaks upon two viruses' infection in rice. Table S5. Nucleotide localization and enrichment of the m6A peaks identified in RSV and RBSDV genomics by m6A-IP-Seq. Table S6. Gene ID and their fpkm analyses. Table S7. Different peaks statistic. Table S8. The m6A peaks appeared in different treatments. Table S9. The most abundant consensus motif in Mock, RSV-, and RBSDV-infected rice plant using suite of DREME and MEME. Table S10. Analyses of the m6A peaks that containing most four common consensus appeared in other species. Table S11. Detail information of ListHits Gene and m6A methylated genes under rice viruses’ infection derived from Additional file 2: Table S16. Table S12. Integrated analyses of the m6A methylation related genes with m6A modifications and expression profiles. Table S13. Integrated analyses of the anti-viral RNA silencing pathway related genes with m6A modification and expression profile. Table S14. Integrated analyses of the plant hormone metabolic genes with m6A modifications and expression profiles. Table S15. Integrated analyses of the relationship betwwen relative expression and m6A positions. Table S16. Summary of the m6A RNA methylation level in enriched top 5 KEGG pathways related genes from RNA-seq under rice viruses’ infection. Table S17. Detail information of the common m6A methylated genes appeared in enriched top 5 KEGG pathways under rice viruses' infection. Table S18. Primers used for qRT-PCR validation of the methylation of OsAGO18 and OsSLRL1 genes. Table S19. Primers used in qRT-PCR qualification of the candidate genes. [file 13059_2021_2410_MOESM2_ESM.zip › Table S19.docx]

**Table S19 Primers used in qRT-PCR qualification of the candidate genes**

| **Pathway Description** | **Gene Name** | **Accession Number** | **Sequences (5′-3′)** | **Tm Value (^o^C)** | **Products (bp)** |
| --- | --- | --- | --- | --- | --- |
| m6A Erasing Proteins | *OsMTA1* | Os01g0267100 | CATCTTTTGTCTTCCTTTGGGTTG | 62.1 | 177 |
|  |  |  | GCTCCTTTGAGTGTTGCAGTATTG | 61.1 |  |
|  | *OsMTA2* | Os02g0672600 | GTGGTTCCCAGCTGAAGGAAT | 61.2 | 168 |
|  |  |  | TTGTGTGACGGCAAGTGTCG | 61.8 |  |
|  | *OsMTA3* | Os03g0147700 | ATGGCAGGGATTCTCAAAGAGTT | 61.2 | 64 |
|  |  |  | AGAGGACCAAAAGGAGGGGG | 61.8 |  |
|  | *OsMTA4* | Os10g0447600 | AGAGCCTCCGTCCCAAGTCA | 61.8 | 138 |
|  |  |  | TTTCAGAGCCAACAACCGTCA | 61.4 |  |
|  | *OsFIP* | Os06g0474200 | TTCAACTCGTCCCCTTCGCC | 64.6 | 123 |
|  |  |  | TTCCACCTTTGTCTTTCCCTTCT | 61.3 |  |
| m6A Methyl-transfererase | *ALKBH10B* | Os10g0116900 | AAGGAAGCACGTTCAGTCGG | 60.1 | 58 |
|  |  |  | AGGAGGTGTCAATGGGGGAG | 60.8 |  |
|  | *ALKBH9B-1* | Os06g0138200 | TCAATATCCTCGCTGGCCTG | 61.0 | 146 |
|  |  |  | CCACCTTTGGGGTTCCGTA | 60.4 |  |
|  | *ALKBH10B-L1* | Os05g0401500 | TTTGGTCTGCCTGTAATTTAGCC | 60.7 | 144 |
|  |  |  | CATCCAGTAGTTCTTTCTCGCAAC | 60.3 |  |
|  | *ALKBH9B-2* | Os12g0122000 | TGGATACACGCTTATTGAGGAGAA | 61.0 | 72 |
|  |  |  | GAGGCAGTGAGAAGGCACAGAT | 60.8 |  |
|  | *ALKBH10B-L2* | Os03g0238800 | CTCACCCTCACCAATCCCCT | 60.8 | 180 |
|  |  |  | CAACATCAGCACTCTTCCCATG | 60.4 |  |
| m6A Reading proteins | *OsYTH01* | Os01g0329800 | GTCATCCTTCCTACAGCCCTTACC | 62.6 | 90 |
|  |  |  | CAGCAGCATCACCAACATATTCAA | 62.7 |  |
|  | *OsYTH02* | Os01g0679900 | ATGGTCGGTCCGGTAGATTTC | 60.4 | 164 |
|  |  |  | CTGCTGTTTGTCACAGGTTTGTTT | 61.2 |  |
|  | *OsYTH03* | Os03g0158500 | GATCTGTGAGCGAGTGGGATG | 60.1 | 137 |
|  |  |  | AACTGGGGAACTAGCAGGGGAG | 63.6 |  |
|  | *OsYTH04* | Os03g0317000 | CGGATGGTGGAGCATTTGTT | 60.5 | 119 |
|  |  |  | ACCGTTTTCTGGTGGGTTAGAA | 60.5 |  |
|  | *OsYTH05* | Os03g0748000 | TGCCCAGTCTTCTTGTTCTTCTC | 60.4 | 123 |
|  |  |  | CCCATTCCACTTGTCTTGTTGC | 61.9 |  |
|  | *OsYTH06* | Os04g0129300 | AATCCAACCATGCTATTTCCCA | 61.1 | 175 |
|  |  |  | CCAACTACATCTCCTGACCCGT | 60.5 |  |
|  | *OsYTH07* | Os04g0608800 | GAAGGGAAGCTTTTTGGGTAAAG | 61.0 | 53 |
|  |  |  | TGGGCTGGTAGATGAAATGTTGT | 61.2 |  |
|  | *OsYTH08* | Os05g0105600 | AGCAGCCTTACTTTTCCCCTTC | 61.2 | 113 |
|  |  |  | TTTGGTTCTCATTTCCCCGAC | 61.3 |  |
|  | *OsYTH09* | Os06g0677700 | GGGCATGTTTCCTATGGGGC | 63.8 | 147 |
|  |  |  | CGGTTATTTGGTGGAGGTGGT | 61.2 |  |
|  | *OsYTH10* | Os07g0170300 | TCAATGGCAAGCAGCAAAAA | 60.3 | 142 |
|  |  |  | GGTGGTCCCAACGGAGAAGTAG | 62.4 |  |
|  | *OsYTH11* | Os08g0224200 | AGGCTAGTTTTTTGGGTAAAGGTG | 60.8 | 75 |
|  |  |  | TTGTTTGTGGCTGAGGTGCG | 62.7 |  |
|  | *OsYTH12* | Os08g0556000 | AGGCGTTGATTGCGAGTTGT | 60.5 | 147 |
|  |  |  | TTGTTCCCGTTTGTGGTGCT | 61.1 |  |
| S-adenosyl-  methionine synthases | *OsSAMS1* | Os05g0135700 | ACACTTATGGTGGCTGGGGAG | 61.3 | 163 |
|  |  |  | CCGATGGCGTATGATACTTGGAC | 63.4 |  |
|  | *OsSAMS1-Like* | Os01g0207300 | CCGAGTCCAGGAACGAGGGTC | 65.4 | 129 |
|  |  |  | CCATGTTTGTCTTGGTGCAGGT | 62.2 |  |
|  | *OsSAM2* | Os01g0323600 | AGACGTTCCTCTTCACCTCCGAGTC | 66.3 | 116 |
|  |  |  | CACCTTGCTGTCGGGGTCCTG | 67.1 |  |
|  | *OsSAM2-like* | Os07g0477250 | CTCCGAGTCCGTGAACGAGGGT | 67.2 | 154 |
|  |  |  | GGTGATCTCGCCGAACACCAT | 64.3 |  |
|  | *OsSAMS3* | Os01g0293000 | GACACCTTCCTCTTCACTTCCG | 60.4 | 117 |
|  |  |  | AACCTTGCTTTCAGGGTCTTCA | 60.6 |  |
| Dicer-like proteins | *OsDCL1a* | Os03g0121800 | TTTTGGGCTTGGGGATTGTA | 60.4 | 159 |
|  |  |  | ATCGGAAGGGTCTCTGGCGT | 63.2 |  |
|  | *OsDCL1b* | Os06g0358800 | TTAGGCAAGCATCCTGTAAGCG | 62.4 | 128 |
|  |  |  | CCAACCATCTCACCGTCAATCA | 62.8 |  |
|  | *OsDCL1c* | Os05g0271300 | TTGCATGAACTATGCCAGAAACAC | 62.0 | 111 |
|  |  |  | ACCAATACCAACTAGCTCCCCA | 60.4 |  |
|  | *OsDCL2a* | Os03g0583900 | AAGGAGGTTGTTTGGAGAAGCA | 60.6 | 168 |
|  |  |  | CCGAGCAATGACCGATGAGA | 61.5 |  |
|  | *OsDCL2b-1* | Os09g0315050 | AAGGAGGTTGTTTGGAGAAGCA | 60.6 | 170 |
|  |  |  | ACCCGAGCAATGACCGATGAGA | 66.3 |  |
|  | *OsDCL2b-2* | Os09g0315100 | AACAACAAAGAAATGCCAAGAAGA | 60.4 | 68 |
|  |  |  | TGAGGAAAGAATCCCCAAGTGT | 60.2 |  |
|  | *OsDCL3a* | Os01g0909200 | CATTTCTGATCCCCTGCTTGG | 61.9 | 68 |
|  |  |  | TGCGTGGTCCTCCTTTACTCG | 62.6 |  |
|  | *OsDCL3b* | Os10g0485600 | CTTGCATTGCCACCTTACCG | 61.5 | 180 |
|  |  |  | TGCTTTTGCCTTTGCCCTCT | 62.4 |  |
|  | *OsDCL4* | Os04g0509300 | TGGGACAGGATGCGGTAAGA | 61.0 | 109 |
|  |  |  | GATGGTTGGGGCAAGGAAGA | 62.0 |  |
| RNA-dependent RNA polymerases | *OsRDR1* | Os02g0736200 | GTGTTCATTCGAGCTACTTCGGG | 62.9 | 94 |
|  |  |  | CTGGGTGGAGGCAAGGGTTT | 63.5 |  |
|  | *OsRDR2* | Os04g0465700 | CGGTGGGAGAAGCCGATGTA | 63.1 | 119 |
|  |  |  | GGTTGTGCTGAGGATGATGAGAG | 61.1 |  |
|  | *OsRDR3* | Os01g0197900 | TGACGGTGACATTTATTGGGTCT | 61.0 | 117 |
|  |  |  | TCCTGAGGTTTCTTTTGCTTGG | 61.2 |  |
|  | *OsRDR4* | Os01g0198000 | GCAAAAGGGACCCGAGGATT | 62.8 | 103 |
|  |  |  | TGCCGTAGCACGCGCATAAC | 65.3 |  |
|  | *OsRDR6* | Os01g0527600 | TGATTGTTTGGTGTTCCCCC | 60.2 | 57 |
|  |  |  | CCAGATGCCTCGTTAGCATGT | 60.2 |  |
| Argonaute proteins | *OsAGO1a* | Os02g0672200 | TCGTGTTCTACCACCCCCCT | 61.4 | 63 |
|  |  |  | GGCAAGACATCCTTCTCTCTGC | 60.6 |  |
|  | *OsAGO1b* | Os04g0566500 | GCCTACCACAAGGTACTCACCAG | 60.5 | 98 |
|  |  |  | AACCACGCCAACTTTCCAAAC | 60.5 |  |
|  | *OsAGO1c* | Os02g0831600 | GATCAACTGGTGAGAAGCCCC | 60.8 | 54 |
|  |  |  | CACTAACGCCATCCCTGTAAAATA | 60.7 |  |
|  | *OsAGO1d* | Os06g0729300 | AGACTGCCTGGGTTTGGTGA | 60.0 | 106 |
|  |  |  | GTGGGTGGTATTGCTGTGCC | 60.8 |  |
|  | *OsAGO2* | Os04g0615700 | CTATTCGGTTATGCCGTTTCG | 60.2 | 176 |
|  |  |  | CAATGTACTTCTGCTTTGTCCTCC | 60.1 |  |
|  | *OsAGO3* | Os04g0615800 | GCCGCAAAACTCGACGAAAA | 63.6 | 146 |
|  |  |  | GCACCGATGAACATGAAATCC | 60.1 |  |
|  | *OsAGO4a* | Os01g0275600 | AAGACAGCATTCCGCAAAACA | 60.8 | 105 |
|  |  |  | CTACAGCCCATCACACCACCA | 61.3 |  |
|  | *OsAGO4b* | Os03g0682600 | CATGTTTACGGCTGGTGCATT | 61.0 | 87 |
|  |  |  | TCCCCCTGGTTTCCTCTCTC | 60.2 |  |
|  | *OsAGO5c* | Os03g0800200 | TGAATCGGAGGAGTTTGTGGTT | 61.0 | 171 |
|  |  |  | ACTTGAATGGTTTCTTGGGGC | 60.6 |  |
|  | *OsAGO6* | Os07g0265600 | AAGATGATGGCATAATAAGGGAACT | 60.1 | 82 |
|  |  |  | AAAATGATTATCTGCTTCGGCTTT | 61.2 |  |
|  | *OsAGO7* | Os03g0449200 | TGGATAATGCAATAGCACGGAG | 60.5 | 96 |
|  |  |  | CAGGATCAAACTGGACAAGGAAA | 60.9 |  |
|  | *OsAGO11* | Os03g0682200 | GCCATTCTGGTATCAAGGGGA | 61 | 128 |
|  |  |  | GGTGCATCGGGCATAGGTGTA | 63 |  |
|  | *OsAGO12* | Os03g0789500 | GGCAACAGCACTTCTCTGTGTG | 60.6 | 135 |
|  |  |  | GATGGGTCCAGGATTTCTTCAAC | 61.6 |  |
|  | *OsAGO13* | Os03g0789466 | TGTCCAGATCCTTCTTCTCTACCC | 60.8 | 58 |
|  |  |  | AGTCCCTCACCAATGTCACCC | 60.7 |  |
|  | *OsAGO14* | Os07g0188000 | TCGTTTCTCGGTCGTTCTACTCC | 62.5 | 60 |
|  |  |  | CCAGCCCATCACCAATGTCTT | 62.0 |  |
|  | *OsAGO15* | Os01g0275200 | AGCGGCCAACATATATCCCA | 60.4 | 160 |
|  |  |  | TCACTGTCCCTCAACACACCAG | 60.4 |  |
|  | *OsAGO17* | Os02g0169400 | AGATGGGCGGAAGAAATACTGTA | 60.1 | 112 |
|  |  |  | CCTGGATGTGGATGGGTGAC | 60.2 |  |
| Resistance Gene | *OsGDI1* | Os05g0418000 | GGATTCCACCTCGCTCAACC | 61.6 | 51 |
|  |  |  | TCCCCTCTAAACCTCTTCCACAG | 61.6 |  |
|  | *OsGDI2* | Os05g0304400 | GAGATTCCACCTCGCTCAACC | 61.0 | 52 |
|  |  |  | TCCCCTCTAAACCTCTTCCACA | 60.4 |  |
|  | *OsGDI3* | Os03g0277000 | GAGATTCCACCTCGCTCAACC | 61.0 | 52 |
|  |  |  | TCCCCTCTAAACCTCTTCCACA | 60.4 |  |
|  | *OsGDIα* | Os07g0271000 | CTCTCCGTCGATCGCCTCAA | 63.3 | 59 |
|  |  |  | TCTCCACCGTAGTAATCATTCCTG | 60.3 |  |
|  | *OsGDI-1-1* | Os01g0913600 | GCTTCCGCTTCAGCTTCATC | 60.0 | 163 |
|  |  |  | GTGGTCTCCTCTTCACCCTCATAT | 60.5 |  |
|  | *OsGDI-1-2* | Os02g0719000 | TTGTCCCGAACCCGGAATCC | 66.3 | 89 |
|  |  |  | GAGCGTGGCAAGGCAAAGCA | 66.7 |  |
|  | *OsGDI-2* | Os06g0318300 | CTTCTTGGCAGCGTCGATTT | 60.5 | 80 |
|  |  |  | GACAGGATGCACAGGCTTGTG | 61.1 |  |
|  | *OsSOT1* | Os11g0505300 | ACTCACATCCCCTACTCCCTCC | 60.9 | 86 |
|  |  |  | ACGTACACGATCCGGCAACC | 62.9 |  |
|  | *OsStvb-i* | Os12g0477700 | GGTCGGCGTCACTGGAGAAT | 62.0 | 111 |
|  |  |  | CAGCGAGAGCAGGAACTTGGT | 61.9 |  |
| JA Biosynthesis | *OsJMT1* | Os05g0102000 | CCCAACGAGGGGAGGATGTA | 61.9 | 112 |
|  |  |  | AGGAGAGGAAGAGGCGGAAGT | 61.1 |  |
|  | *OsJMT-1* | Os06g0314600 | TCTTAGGGTGTCGAGGCGTG | 60.9 | 165 |
|  |  |  | TTGGATCTTGAGATAGGGTTTTCC | 60.9 |  |
|  | *OsAOS1* | Os03g0767000 | TGGTGAAGAAGGACTACGACCGC | 65.3 | 144 |
|  |  |  | CGCCGAACGAGTTGAAGCAGA | 65.9 |  |
|  | *OsAOS2* | Os03g0225900 | AATGGGCGAGAGACGGAGAA | 61.9 | 175 |
|  |  |  | CGGAGGTTGAAGCTTTGGTGA | 62.5 |  |
|  | *OsLOX1* | Os02g0194700 | CCTCCTCCACCTCCACCAACAT | 64.7 | 138 |
|  |  |  | CGTCTCTGAACCACGAGAACCTAT | 61.7 |  |
|  | *OsLOX5* | Os03g0700700 | TGCCTGCACCTTTGATCCCT | 62.6 | 67 |
|  |  |  | CCAATTTTACCGTCCCCTCGT | 62.8 |  |
|  | *OsLOX9* | Os08g0508800 | CTAAGTCGGAGACGAGGAAGGG | 62.1 | 177 |
|  |  |  | AGAAGGACGGGAACGGAAGG | 62.8 |  |
|  | *OsLOX2* | Os03g0179900 | TCAATGCGATAGATGGACGGA | 61.2 | 112 |
|  |  |  | GGCTTGGCTGGAGGAAGACT | 60.7 |  |
|  | *OsLOX8* | Os08g0509100 | ACGACCGCGTCTACGACTACG | 62.7 | 106 |
|  |  |  | GGGTAAGGGAACTGCTTGCTG | 61.4 |  |
|  | *OsHPL3* | Os02g0110200 | CACGGCATCAGCAAGGAGGA | 64.1 | 96 |
|  |  |  | GACCAGGAACGGCAGGAACA | 62.9 |  |
| JA Responsive | *OsPR1a* | Os07g0129200 | TGTCGGAGAAGCAGTGGTACG | 61.0 | 162 |
|  |  |  | AGTTGCAGGTGATGAAGACGC | 60.1 |  |
|  | *OsPR1* | Os07g0129300 | CGGCGAGAACATCTTCTGGG | 62.8 | 101 |
|  |  |  | TGCTGTCGTGGTCGTACCAC | 61.1 |  |
|  | *OsPR1b* | Os01g0382000 | AAGCTGGCCATTGCTTTGGC | 64.9 | 73 |
|  |  |  | AGTCCTGCGGGGAGTTTTGA | 61.8 |  |
|  | *OsPR2* | Os01g0194300 | AGGACAAGGAGGAGAATGACAGG | 61.3 | 159 |
|  |  |  | GAAGGCAACCATCAGGTGAGTAG | 60.5 |  |
|  | *OsPR5-1* | Os01g0122000 | CAGGAGGAAGAGGAGGCGGAGGA | 69.8 | 146 |
|  |  |  | AGGCAGGCATGGCAGTGGAGC | 69.1 |  |
|  | *OsPR5-2* | Os03g0661600 | GACGCCTCCAGCACCTACACT | 61.6 | 126 |
|  |  |  | TCATACAAATCCCAAGATCATCAGA | 60.6 |  |
|  | *OsPR5-3* | Os04g0689900 | AGAGGTGGTGTTGTGTGGTTGG | 61.9 | 102 |
|  |  |  | CGTCTTGCACTGGTTGATGATG | 61.3 |  |
|  | *OsPR5-4* | Os12g0628600 | CTGTTCCCCGAAGACAACACC | 61.6 | 72 |
|  |  |  | TGGGCAGAAGACGACTTGGTAG | 61.8 |  |
|  | *OsbZIP52* | Os06g0662200 | CAGAGTACACTGGACGGAAGCG | 62.3 | 148 |
|  |  |  | AGGTGGTTGGTTTGGGGTCAT | 62.7 |  |
|  | *OsMYB2* | Os01g0285300 | TACACTCGCAGCAATCCCACT | 60.6 | 90 |
|  |  |  | CCCTCCCAAGAAACTTCCACA | 61.4 |  |
|  | *OsMYB55/61-L* | Os05g0140100 | CCAACCCTGTATTTTCCTTTCCA | 62.3 | 56 |
|  |  |  | GTGCCACATTCATTACTGTAGCCT | 60.4 |  |
|  | *OsWRKY10* | Os01g0186000 | CGGCTGTTCCTTTGGTGAATT | 61.6 | 124 |
|  |  |  | CGACGACACGCCATACTGATC | 61.2 |  |
|  | *OsWRKY28* | Os06g0649000 | GTGAAGGATGGGTATCAATGG | 60.8 | 89 |
|  |  |  | GCGAACGAGCACCTGAAGTAG | 60.2 |  |
|  | *JIOsPR10* | Os03g0300400 | CTCAGCCATGCCATTCAGCC | 63.6 | 69 |
|  |  |  | TTGCACTCGCACTTGTCCAC | 60 |  |
|  | *OsJAmyb* | Os11g0684000 | GATAGCGGAGACTACAGCAACAAC | 60.3 | 89 |
|  |  |  | CTCGGAGAGCCATCAAAACAA | 60.3 |  |
|  | *OsRbohA* | Os01g0734200 | ACAAGCCAAGCACTGAGCCA | 61.5 | 132 |
|  |  |  | CCCAATCCACAATGCAAGCA | 62.4 |  |
|  | *OsRbohB* | Os01g0360200 | CCAAGGGAATAACGGACGAAA | 61.6 | 114 |
|  |  |  | CCAGCCCGATGAGAAGTAGCA | 62.6 |  |
|  | *OsRbohC* | Os05g0528000 | CTACCAAGAAAGCCTTACCCAAA | 60.1 | 107 |
|  |  |  | GCCAATTCCTAATCCAACCAGC | 62.7 |  |
|  | *OsRbohD* | Os05g0465800 | CCCAGATGTTTCGCCTTTTG | 60.6 | 158 |
|  |  |  | CCTTCTTGGAACTTACTTGTGCCT | 61.3 |  |
|  | *OsRbohE* | Os01g0835500 | TGGCATCCCTTCTCCATCAC | 60.2 | 114 |
|  |  |  | CTCGCAAGCCTTCCCAAACA | 63.5 |  |
|  | *OsRbohF* | Os08g0453700 | TAGGCATGGCAGAACAGAAGAGT | 60.8 | 106 |
|  |  |  | AGGCCGACAAGGAGAAGAACA | 61.3 |  |
| SA Biosynthesis | *OsICS1* | Os09g0361500 | ATGTTTAAAGGTTGAGGGCCAA | 60.7 | 68 |
|  |  |  | CAAATGCAGGAGCATCAGGTG | 61.3 |  |
|  | *OsPAL* | Os02g0626400 | TTGGACTATGGGTTCAAGGGC | 61.4 | 89 |
|  |  |  | ACATGGTTGGTCACCGGGTT | 61.9 |  |
|  | *OsPAL1* | Os02g0626100 | TCGTTCCCGCTCTACCGCTT | 64.5 | 96 |
|  |  |  | CACCTTGTTGCACTCCTCGC | 61.3 |  |
|  | *OsAIM1* | Os02g0274100 | GCTCTTCTTGTTAGCAATACATCCG | 61.9 | 112 |
|  |  |  | AGCCCCTCTTGACTAGACCCTC | 60.7 |  |
|  | *OsCM* | Os01g0764400 | GTGGATGGATTTGATGGCTCTT | 60.5 | 70 |
|  |  |  | CAACCTGTTGATGTAGTTTTTCGG | 60.9 |  |
|  | *OsEDS1* | Os09g0392100 | CTTTGGGGCTCCTCTTGTTGG | 63.4 | 113 |
|  |  |  | GTATGCGGGGGATGATGTCTAC | 60.9 |  |
| SA Responsive | *OsPR1-101* | Os10g0191300 | GGCCAAGTAGACGACCACTCAC | 61.1 | 71 |
|  |  |  | ATGTACTGCGGCAGCGACATA | 61.7 |  |
|  | *OsWRKY45-1* | Os05g0322900 | TGGAGGACATGGAGAAGGGG | 61.8 | 109 |
|  |  |  | CGGGAGAAGGTGTGGAGAATC | 60.8 |  |
|  | *OsWRKY45-2* | Os05g0322900 | TGGAGGACATGGAGAAGGGG | 61.8 | 109 |
|  |  |  | CGGGAGAAGGTGTGGAGAATC | 60.8 |  |
|  | *OsSGT1* | Os09g0518200 | AGGGAGGTGATGGAAGGGGA | 62.7 | 115 |
|  |  |  | CGACGATATTTTTGTCGGAGC | 60.1 |  |
|  | *OsPR1b* | Os01g0382000 | AAGCTGGCCATTGCTTTGGC | 64.9 | 73 |
|  |  |  | AGTCCTGCGGGGAGTTTTGA | 61.8 |  |
|  | *OsPR1a* | Os07g0129300 | CGGCGAGAACATCTTCTGGG | 62.8 | 101 |
|  |  |  | TGCTGTCGTGGTCGTACCACT | 61.1 |  |
|  | *OsPR1-12* | Os01g0382400 | CGTGGTAGCCGCTACAATGTT | 60.5 | 122 |
|  |  |  | GCTTCGTGCTCCAGCTCATC | 60.6 |  |
|  | *OsPR1-21* | Os02g0786500 | AACCAACCAATCTTGCTGTCTCA | 61.3 | 112 |
|  |  |  | GATGTTGCAGCGTGCAGTAGAA | 61.4 |  |
|  | *OsPR1-22* | Os02g0786900 | ACGATTCTTTCAGGTTTCTTCACA | 60.1 | 51 |
|  |  |  | CCACCAAGACTTTCACGGATTT | 60.8 |  |
|  | *OsPR1-51* | Os05g0595000 | TGGTTTCTGCTGCTGTCTGTGG | 63.4 | 61 |
|  |  |  | ATGCTGATGCGTCGTCGTTG | 62.6 |  |
|  | *OsPR1-121* | Os12g0633400 | TGGAAGAAGACGGTGGACGA | 61.0 | 106 |
|  |  |  | AGACGATGGCGGTGTAGTGG | 60.6 |  |
| ABA Biosynthesis | *OsNCED1* | Os03g0645900 | GGAGGGGTTTGTGGCGAATG | 64.7 | 153 |
|  |  |  | TTGATGAAGGGCGGGATGCG | 68.4 |  |
|  | *OsNCED3* | Os07g0154100 | GTGGTATACGACAGGGAGAAGGC | 61.9 | 115 |
|  |  |  | TCCAGAGGTGGAAGCAGAAGC | 61.7 |  |
|  | *OsABA2* | Os03g0810800 | CATGGGATACGCGTGAACTGC | 63.6 | 122 |
|  |  |  | CCACCAACAAAGGCGAAAAAG | 62.3 |  |
|  | *OsABA1* | Os04g0448900 | TGGATTACCGACAACGAAGGTAG | 61.1 | 67 |
|  |  |  | AGGGATGGAAACGGACAGGG | 63.3 |  |
|  | *OsbZIP72* | Os09g0456200 | CCGCCGTCGCTATGCAAACA | 67.4 | 120 |
|  |  |  | TTCTCCCCCTCACCAACCCTTC | 66.1 |  |
|  | *OsbZIP23* | Os02g0766700 | CGGTGCCGTACGTTTTCAAA | 62 | 85 |
|  |  |  | ATCCGCCGCTGTCTTCTCTC | 61.6 |  |
| ABA Deactivation | *OsABA8OX1* | Os02g0703600 | GCCGTGGAGGACGTGGAAT | 62.9 | 180 |
|  |  |  | GGGAGGGAAGGGAACAAAAA | 60.5 |  |
|  | *OsABA8OX2* | Os08g0472800 | TGGACGCAGACGAGGAGCAT | 63.6 | 137 |
|  |  |  | CACCCCTTTGGAATCAGGAAAC | 62.4 |  |
|  | *OsABA8OX3* | Os09g0457100 | TCGTCCTCATCCACCACCTG | 61.0 | 139 |
|  |  |  | TCCACACTGACACTATCATCCCTC | 60.5 |  |
| Auxin Biosynthesis | *OsYUCCA1* | Os01g0645400 | GAGCAGTGAAGGAGATGACGAGG | 62.8 | 173 |
|  |  |  | GCAGTGCGGACAGAAAGAAAAATA | 62.9 |  |
|  | *OsYUCCA4* | Os01g0224700 | GTTTCTCAAGGCGTGGTCTCTC | 60.5 | 68 |
|  |  |  | CTGCACAATATCCTGCACAATGTT | 61.9 |  |
|  | *OsYUCCA5* | Os12g0512000 | GAAATAAAGGTGGTCCCAGCAA | 60.9 | 83 |
|  |  |  | GCATCAAAGTCCTCCCTACAGC | 60.6 |  |
|  | *OsYUCCA6* | Os07g0437000 | TACCAAAGCAACGTCCCCCA | 63.4 | 98 |
|  |  |  | GACTCCCCCTTCCAACCATCT | 61.6 |  |
|  | *OsYUCCA7* | Os04g0128900 | AAGGGTGAGTCGGGGCTGTA | 61.7 | 137 |
|  |  |  | GCCGTGCTCTTCTTGGTTGG | 62.9 |  |
|  | *OsYUCCA8* | Os03g0162000 | GATGCCGTCATCTTCGCCAC | 63.4 | 73 |
|  |  |  | TGAACAACTCACCGTCCTCCTG | 62.2 |  |
|  | *OsYUCCA9* | Os01g0273800 | GGAGGGGTTCAAGGGGAAGT | 61.6 | 110 |
|  |  |  | TGCGGATGACGATGGAGGTG | 64.6 |  |
|  | *OsYUCCA10* | Os01g0274100 | CTTCGACGCCATCGTCTTCG | 63.9 | 138 |
|  |  |  | TACAGCCCGTTCTCCCCCTT | 63.2 |  |
|  | *OsTAR2* | Os01g0169800 | TGCGGTGCGATAGGGAGGAT | 64.9 | 164 |
|  |  |  | CGACGAGAGGCGGTTGATGA | 64.1 |  |
|  | *OsTARL2* | Os01g0169800 | GCTTCAGAGCATAACCAAACGG | 61.7 | 62 |
|  |  |  | ACTTCAATCAACCTCACTCCCATT | 61.0 |  |
|  | *OsTAA1* | Os01g0717700 | GCTGATAAGGGACGAGAAGGTG | 60.6 | 158 |
|  |  |  | CATAGCCGAAGGCGAAGATGT | 61.9 |  |
|  | *OsAO-1* | Os03g0790700 | ATTCCATATCACAACGTCCGTATC | 60.0 | 67 |
|  |  |  | TCATTGCCTTTCCACCAAAAC | 60.1 |  |
|  | *OsAO-2* | Os07g0281800 | GTGCAACATGCTGATTGAGAGATT | 61.1 | 103 |
|  |  |  | TTCCTGAGAGGCCTGGGAAA | 61.9 |  |
|  | *OsAAO* | Os07g0164900 | CAGCTAAAGAACTCTACGTTCCCG | 62 | 178 |
|  |  |  | AAAATGCTCACCTGCCCCAA | 62.7 |  |
|  | *OsAAO3-1* | Os03g0790900 | CCCATTTTTAGCTCCTACGCC | 60.9 | 93 |
|  |  |  | GATTCAAGTTTCACCTCCCCATC | 61.6 |  |
|  | *OsAO1-L* | Os03g0791200 | GCTCGGCGATGACTTTCCAGA | 64.9 | 148 |
|  |  |  | TGCAAGCGAAGCCAATGAACC | 65.7 |  |
|  | *OsAO3-L* | Os07g0282500 | CCAAGCAGCGAGGATAGAGTTT | 60.6 | 102 |
|  |  |  | AAGCCGCACAGTTCCTTCACC | 63.9 |  |
|  | *OsAAO3-2* | Os07g0282300 | CCACTGAGGGTCTGGGGAAT | 60.8 | 82 |
|  |  |  | CCACACTGGGAGGCATGAAA | 61.3 |  |
|  | *OsGH3.8* | Os07g0592600 | GCGTGTGGCCGAACACCAAGTA | 67.6 | 92 |
|  |  |  | AAGCCCGCCGCTGTAGAACT | 63.8 |  |
|  | *OsGH3.2* | Os01g0764800 | TCATCTCCTCCCACCCCATC | 61.5 | 175 |
|  |  |  | TAGAGCCCCTTGCCCTTGTC | 61.6 |  |
| Auxin Transport | *OsPIN1A* | Os06g0232300 | TACGGGTCGGTGAAGTGGTG | 61.3 | 126 |
|  |  |  | GGTGTAGGGGTTGTTGGTGGA | 61.8 |  |
|  | *OsPIN1B* | Os02g0743400 | TACGGGTCGGTGAAGTGGTG | 61.3 | 119 |
|  |  |  | GGGTTGTTGGTGGAGATGAAGT | 60.3 |  |
|  | *OsPIN2* | Os01g0643300 | ATCCGCAACCCCAACACTTA | 60.3 | 160 |
|  |  |  | CCATGAACAATCCCAGGCTAAAC | 62.6 |  |
|  | *OsPIN5b* | Os01g0919800 | GCCCTACCTCAATCCATCACAT | 60.4 | 51 |
|  |  |  | CAGCCCGTATTCTTTCGCAA | 61.3 |  |
|  | *OsPILS1* | Os08g0191000 | CATACCACGGATCACCCCTTT | 61.1 | 160 |
|  |  |  | TCTCGTTCCCTTCGCCTACA | 60.6 |  |
|  | *OsPILS5* | Os01g0818000 | GCTCTTTGATTGGCTTTGTGGT | 61.1 | 59 |
|  |  |  | CTTGAAGTATGGATAAGGTGGTCG | 60.1 |  |
|  | *OsPILS6b* | Os05g0481900 | TGCTGGTATTGGCATCGTCTC | 60.9 | 70 |
|  |  |  | CTTGAACATCTTGTCTCCTTTTGG | 60.1 |  |
|  | *OsPILS7a* | Os09g0554300 | CAACCCGTTCGGAAAAGACC | 61.8 | 86 |
|  |  |  | TGAATAAGCCACCAAGAGCCAT | 61.4 |  |
|  | *OsPILS7b* | Os09g0555100 | CACGATTCCTTGCGTCACCC | 63.4 | 65 |
|  |  |  | GCTCCGACTTCCTTAGCCCTTT | 63.3 |  |
|  | *OsPIN5A* | Os01g0919800 | GCCCTACCTCAATCCATCACAT | 60.4 | 51 |
|  |  |  | CAGCCCGTATTCTTTCGCAA | 61.3 |  |
|  | *OsPIN3A* | Os08g0529000 | GGATGGGGAGATGTGTACAAGG | 60.7 | 167 |
|  |  |  | AAGTCGAAGGTGAAGAAGGGC | 60.1 |  |
| Auxin Signaling | *OsIAA3* | Os12g0601400 | AGACGCAGCAGAAGGAGGATG | 61.8 | 158 |
|  |  |  | GCCGGAGAAGCAGAGGAACAT | 63.1 |  |
|  | *OsIAA7* | Os02g0228900 | AGCCCAAAGAGACCCCCTCA | 63.1 | 118 |
|  |  |  | CGCCTTCATAATCCTCATAAACCA | 62.6 |  |
|  | *OsIAA20* | Os06g0166500 | CGGTGGCGGATATGTGAAGG | 63.4 | 176 |
|  |  |  | CGTAGGTGACAGCGTATGGGTG | 62.8 |  |
| CTK Biosynthesis | *OsIPT3* | Os05g0311801 | AAGGTAGTGGTGGTGATGGGC | 61.1 | 106 |
|  |  |  | CCTGGATCTTGTCGGAGTTGAT | 60.5 |  |
|  | *OsIPT7* | Os05g0551700 | CGAGGATACGAGGATGGTGG | 60 | 113 |
|  |  |  | GAATCTTGTCGGCGTTGACAA | 61 |  |
|  | *OsIPT9* | Os01g0968700 | GCTCTTGTTAGCCCATTCCTCTT | 61.2 | 169 |
|  |  |  | GGTTGTTCGGGTGTATCCTTTG | 61 |  |
|  | *OsIPT10* | Os06g0729800 | ATTGGATCACAAGGACACAGAGG | 60.5 | 82 |
|  |  |  | GCGGGAAAAGAGGGGGAAGA | 65.1 |  |
| CTK Oxidase | *OsCKX4* | Os01g0940000 | TGGAGATTGTGACAGGGAGGG | 61.9 | 80 |
|  |  |  | GCCAAGAGCAGCGTAGAAGAGA | 61.6 |  |
|  | *OscZOGT1* | Os04g0556500 | GAAAGGCTTGGATGACTTCGTT | 60.2 | 53 |
|  |  |  | AGACATTCCTGCTTCATCTTGTGA | 60.7 |  |
|  | *OscZOGT2* | Os04g0556600 | GGTTCATCTGGGTGCTTCGC | 62.6 | 109 |
|  |  |  | ACTCCCTCGGTTTCTGCGGT | 63.2 |  |
| CTK Response | *OsPR1a-1* | Os07g0129200 | CCTGCTGCTTGCTGGTGCTCG | 68.7 | 82 |
|  |  |  | TTGTGCGGGTCCACGAAGTCC | 67.5 |  |
|  | *OsPR1a-2* | Os07g0129300 | CGGCGAGAACATCTTCTGGG | 62.8 | 101 |
|  |  |  | TGCTGTCGTGGTCGTACCAC | 61.1 |  |
|  | *OsPR1b* | Os01g0382000 | AAGCTGGCCATTGCTTTGGC | 64.9 | 73 |
|  |  |  | AGTCCTGCGGGGAGTTTTGA | 61.8 |  |
|  | *OsPR2-1* | Os01g0940700 | AACACTGGCATTGGTCCTTGG | 62.1 | 133 |
|  |  |  | GATGCCGTTGGACTTGTAGAGC | 61.4 |  |
|  | *OsPR2-2* | Os01g0944700 | GAGAACCAGAAGAACGGGGATC | 61.6 | 66 |
|  |  |  | GGGCGACTTGTTAGGGTAGAAGAG | 62.9 |  |
|  | *OsPR2-3* | Os05g0375400 | CCTTTGCCTCCATTCCTCAAA | 61.7 | 53 |
|  |  |  | ATGCCGTAGCACACCCCGA | 64.7 |  |
|  | *OsPR3* | Os04g0493400 | GTGTCTGTGGAGAGCGTGGTCA | 63.1 | 163 |
|  |  |  | GCTTGGAGTCGTCGTTGGTG | 61.1 |  |
|  | *OsPR4* | Os11g0592200 | ACGTGCGAGCCACGTATCAT | 61.2 | 151 |
|  |  |  | GGACCAGCAGGTCCACAGAA | 60.3 |  |
|  | *OsPR5-1* | Os04g0689900 | AGAGGTGGTGTTGTGTGGTTGG | 61.9 | 102 |
|  |  |  | CGTCTTGCACTGGTTGATGATG | 61.3 |  |
|  | *OsPR5-2* | Os12g0628600 | CTGTTCCCCGAAGACAACACC | 61.6 | 79 |
|  |  |  | TGCATTATGGGCAGAAGACGA | 61.5 |  |
|  | *OsPR6* | Os12g0437800 | GAGCATAGAGGAGGCCAAGAAG | 60.1 | 110 |
|  |  |  | GGACACGGTTGAAGCGAAAA | 60.9 |  |
|  | *OsPR8* | Os10g0416500 | ACGAGACCAAGGCTCAGGAGA | 61.1 | 54 |
|  |  |  | ACCGTTCGATGGACGATCAGT | 61.5 |  |
|  | *OsPR9* | Os07g0677200 | GGACGGTTCTGTTGGGGAGA | 61.8 | 72 |
|  |  |  | GGGCAGGGAGGTCGGTATTT | 62.6 |  |
|  | *OsPR10* | Os03g0300400 | CTCAGCCATGCCATTCAGCC | 63.6 | 69 |
|  |  |  | TTGCACTCGCACTTGTCCAC | 60 |  |
|  | *OsPR10a-1* | Os12g0555000 | TGGATGATGGGGGGTCATTC | 62.2 | 133 |
|  |  |  | TTCGTCTCTGTCACGTGCGA | 60.6 |  |
|  | *OsPR10a-2* | Os12g0555500 | TGGTCCGGGCACCATCTACA | 64 | 143 |
|  |  |  | GCTTCCCCACCTTGCTTTCT | 60.8 |  |
| ET Biosynthesis | *OsACS1* | Os03g0727600 | TCGGAGCAGAGAGGGTACAAGG | 63.2 | 134 |
|  |  |  | TATGGGGTGGGGATGAGGAA | 62 |  |
|  | *OsACS2* | Os04g0578000 | TGGAGCAAGAACCACCCCGA | 65.7 | 140 |
|  |  |  | CCCCTCACCTGCCCCATAAA | 64.2 |  |
|  | *OsACS6* | Os06g0130400 | GTTGATACCTGTTCCTTGCCGT | 60.8 | 98 |
|  |  |  | CCTTTATTCCTCGCTTCTTTGC | 60.5 |  |
|  | *OsACO7* | Os01g0580500 | GTGGAGGCGGCGTTAATGGA | 65.4 | 91 |
|  |  |  | CGAGGTCGGAGGCGTAGAAT | 61.6 |  |
|  | *OsACO3* | Os02g0771600 | TCCATCGCCTCCTTCTACAACC | 63 | 108 |
|  |  |  | CTCGAACACGAACCTCGGGT | 62.1 |  |
|  | *OsACO5* | Os05g0149400 | TCGGGTGAAGAAGGTGTGCT | 60.2 | 176 |
|  |  |  | ACTGGTTGTCGTCGTGGAGG | 60.3 |  |
|  | *OsACO1* | Os09g0451000 | TCTACAACCCTGGCAGCGAC | 60.9 | 93 |
|  |  |  | CGAACACGAACTTGGGATACGT | 61.6 |  |
|  | *OsACO2* | Os09g0451400 | ACGTGAATAAGGCGGAGAAGC | 61.1 | 52 |
|  |  |  | TGGCGGACGAAGAAGGTGCT | 65.2 |  |
| BR Biosynthesis | *OsD11* | Os04g0469800 | TGCGAGGAGGCAAGAAAGTT | 60 | 146 |
|  |  |  | CCTGGGATGTAGAGAGGGAAAGA | 61.5 |  |
|  | *OsD11-L* | Os06g0317200 | AATCTCCTTCTGCTTGGTGTCTTC | 61.0 | 113 |
|  |  |  | GGCTTCACATTCTTCCCCTCA | 61.4 |  |
|  | *OsDWARF4* | Os03g0227700 | CCCTCGCCATCTTCTTCCTT | 60.7 | 97 |
|  |  |  | TCCCCTCTTAGCCTTTGTCTC | 61.6 |  |
|  | *OsCPD1* | Os11g0143200 | CGGGAGGTGATAAAGAAGAGGA | 60.2 | 167 |
|  |  |  | ACCAGCAGAGAAAGGCAGAAAT | 60 |  |
|  | *OsCPD2* | Os12g0139300 | ATCCCCTTTCCTCTCGCCTA | 60.4 | 155 |
|  |  |  | TTCCCCTCATCATCCCCAAT | 61.1 |  |
|  | *OsD2* | Os01g0197100 | ATGAGCCCTACAAGTTCAACCCA | 62.6 | 86 |
|  |  |  | CCTCTGCCCACCACCAAAAG | 62.6 |  |
|  | *OsSK11* | Os01g0252100 | ACCAGCGTATGCCACTTATCTATG | 60.6 | 144 |
|  |  |  | TGAGTTGATGGGTATGTGGGTTT | 60.9 |  |
|  | *OsGSK2* | Os05g0207500 | GGCACATCATCTCCACCACC | 60.3 | 142 |
|  |  |  | GCAACAGTCTCTCCTGTCTCCAA | 60.9 |  |
|  | *OsRAV1* | Os01g0140700 | CAGCAGGCCGAGAGGCATTT | 64.9 | 160 |
|  |  |  | ACCCCTTGGTGAGCACGTAA | 60.2 |  |
|  | *OsRAV2* | Os01g0141000 | ATCAGCGTACTCCTGCCCTAGG | 62.5 | 118 |
|  |  |  | TGAAACAACAGACGATGAACAACAA | 62.3 |  |
|  | *OsRAVL1* | Os04g0581400 | GGAGCAGGAGCAGGATGAAGA | 61.6 | 110 |
|  |  |  | ATGTCGGGGAGGACGATGAG | 61.8 |  |
|  | *OsBZR2* | Os01g0203000 | GGTGGTTCCATTAGTGCCCC | 61.4 | 78 |
|  |  |  | CCAATCTGTCTTCATGCGAGG | 60.4 |  |
|  | *OsBZR3* | Os06g0552300 | CTTGATTGGTGGTGTCGAGGG | 62.4 | 178 |
|  |  |  | TAAGCGAGGTGTGCGAGTAGGG | 64.3 |  |
|  | *OsBZR1* | Os07g0580500 | CCTACAACCTCGTCAACCCG | 60.3 | 62 |
|  |  |  | GGCCGTCCCTTCTATCTCCAT | 62 |  |
| BR Signaling | *OsBRI1-1* | Os01g0718300 | GAGGAGCGGTTGTTGGTGTATG | 61.9 | 177 |
|  |  |  | TCGGTGAATGATGTGAGGAATG | 60.5 |  |
|  | *OsBRI1-2* | Os10g0114400 | TGGGAGCAGCGGAAGAAGGT | 64.3 | 61 |
|  |  |  | TGTAGTGGAGGAAGCACAGGC | 60.1 |  |
|  | *OsBAK1-4* | Os01g0171000 | CGAGGAAATGGTTCAGGTGG | 60.1 | 60 |
|  |  |  | CTGGGACGATGAGATGGGTAGT | 60.4 |  |
|  | *OsBAK1-9* | Os02g0236100 | ACTGATCACTGGGCCTAAAACCT | 61.2 | 167 |
|  |  |  | CGTCAACAGAACACTCCAACTCG | 62.2 |  |
|  | *OsBAK1-10* | Os03g0266800 | CGGATCGATTCCACCCTCTC | 61.5 | 154 |
|  |  |  | CTGCTTCCCACATAAACCACG | 60.6 |  |
|  | *OsI-BAK1* | Os03g0440900 | CCAAGGGAGCTGGCCAAACT | 63.8 | 90 |
|  |  |  | GAAGGGACCGTCAACAGGAATA | 60.7 |  |
|  | *OsBAK1-6* | Os03g0703200 | ATATCTGTCCACAGGGAAGTCATCG | 63.5 | 138 |
|  |  |  | TGGTCAAGCAGTAGCACATCGTC | 62.7 |  |
|  | *OsBAK1-2* | Os04g0457800 | ATTCTCACTCCGGGAGCTTCA | 61.2 | 152 |
|  |  |  | CACCAGGGGTACGTTCTTCTTTTA | 61.9 |  |
|  | *OsBAK1-3* | Os06g0225300 | GCAACCCCAAGATTATCCACC | 63.2 | 79 |
|  |  |  | TTCTTCCTGATGGCACCGCT | 62.8 |  |
|  | *OsBAK1-8* | Os11g0607200 | GCAACCCCAAGATTATCCACC | 60.8 | 90 |
|  |  |  | CCAACCCAAAGTCTCCTACCAC | 60.2 |  |
