# Supplementary material for: The dynamics of N6-methyladenine RNA modification in interactions between rice and plant viruses
Source: Genome Biol. 2021 Jun 24;22:189. doi: 10.1186/s13059-021-02410-2 (PMC8229379; doi:10.1186/s13059-021-02410-2)
Supplement: Supplementary file 2 — Additional file 2: Table S1. Primers used for RSV and RBSDV detections in this study. Table S2. Sequenced and rice genome mapped reads in m6A-IP-seq, input RNA-seq rice samples. Table S3. Nucleotide localization and enrichment of the top 10 m6A peaks identified in Mock-, RSV-, and RBSDV-infected rice transcripts by m6A-IP-Seq. Table S4. The category of the differential m6A peaks upon two viruses' infection in rice. Table S5. Nucleotide localization and enrichment of the m6A peaks identified in RSV and RBSDV genomics by m6A-IP-Seq. Table S6. Gene ID and their fpkm analyses. Table S7. Different peaks statistic. Table S8. The m6A peaks appeared in different treatments. Table S9. The most abundant consensus motif in Mock, RSV-, and RBSDV-infected rice plant using suite of DREME and MEME. Table S10. Analyses of the m6A peaks that containing most four common consensus appeared in other species. Table S11. Detail information of ListHits Gene and m6A methylated genes under rice viruses’ infection derived from Additional file 2: Table S16. Table S12. Integrated analyses of the m6A methylation related genes with m6A modifications and expression profiles. Table S13. Integrated analyses of the anti-viral RNA silencing pathway related genes with m6A modification and expression profile. Table S14. Integrated analyses of the plant hormone metabolic genes with m6A modifications and expression profiles. Table S15. Integrated analyses of the relationship betwwen relative expression and m6A positions. Table S16. Summary of the m6A RNA methylation level in enriched top 5 KEGG pathways related genes from RNA-seq under rice viruses’ infection. Table S17. Detail information of the common m6A methylated genes appeared in enriched top 5 KEGG pathways under rice viruses' infection. Table S18. Primers used for qRT-PCR validation of the methylation of OsAGO18 and OsSLRL1 genes. Table S19. Primers used in qRT-PCR qualification of the candidate genes. [file 13059_2021_2410_MOESM2_ESM.zip › Table S2.docx]

**Table S2** Sequenced and rice genome mapped reads in MeRIP-seq, input RNA-seq rice samples

| **Replicate 1** | | | | | | | | | | | |
| --- | --- | --- | --- | --- | --- | --- | --- | --- | --- | --- | --- |
|  | **Sample^a^** | **Treatment** | **Raw reads** | **Clear reads** | **Clear rate %** | **Mapping to Rice genome** | | | **Mapping to viral genome** | | |
|  |  |  |  |  |  | **Reads number** | **Map%** | **Reads Number** | | **Map%** |  |
| **IP** | **HD5** | HD5_Mock | 122759158 | 108036244 | 88.00 | 69400434 | 64.24 | 0 | | 0 |  |
|  |  | HD5_RSV | 119784458 | 105778498 | 88.31 | 60939347 | 57.61 | 52889249 | | 1.25 |  |
|  |  | HD5_RBSDV | 101463514 | 87523208 | 86.26 | 42071740 | 48.07 | 43761604 | | 0.01 |  |
| **Input** |  | HD5_Mock | 112794356 | 110072492 | 97.59 | 108379990 | 98.46 |  | | 0 |  |
|  |  | HD5_RSV | 174973258 | 171203352 | 97.85 | 99925500 | 58.37 | 85601676 | | 31.80 |  |
|  |  | HD5_RBSDV | 134364157 | 131244762 | 97.68 | 127190163 | 96.91 | 65622381 | | 1.49 |  |
| **Replicate 2** | | | | | | | | | | | |
|  |  | **Treatment** | **Raw reads** | **Clear reads** | **Clear rate %** | **Mapping to Rice genome** | | | **Mapping to viral genome** | | |
|  |  |  |  |  |  | **Reads Number** | **Map%** | **Reads Number** | | **Map%** |  |
| **IP** | **HD5** | HD5_Mock | 112604213 | 99537440 | 88.40 | 51885788 | 52.13 | 0 | | 0 |  |
|  |  | HD5_RSV | 116233256 | 103768376 | 89.28 | 52132803 | 50.24 | 51884188 | | 0.91 |  |
|  |  | HD5_RBSDV | 143663984 | 127562870 | 88.79 | 79940143 | 62.67 | 63781435 | | 0.02 |  |
| **Input** |  | HD5_Mock | 121504892 | 118174329 | 97.26 | 116291156 | 98.41 | 0 | | 0 |  |
|  |  | HD5_RSV | 166831254 | 163198600 | 97.82 | 94317811 | 57.79 | 81599300 | | 32.28 |  |
|  |  | HD5_RBSDV | 175132545 | 170803172 | 97.53 | 165547399 | 96.92 | 85401586 | | 1.45 |  |

Note: ^a^ stands for Rice cultivar “Huaidao 5”, HD5;
