# Supplementary material for: The dynamics of N6-methyladenine RNA modification in interactions between rice and plant viruses
Source: Genome Biol. 2021 Jun 24;22:189. doi: 10.1186/s13059-021-02410-2 (PMC8229379; doi:10.1186/s13059-021-02410-2)
Supplement: Supplementary file 3 — Additional file 3:. Review history. [file 13059_2021_2410_MOESM3_ESM.docx]

Review History

**First round of review**

**Reviewer 1**

**Are you able to assess all statistics in the manuscript, including the appropriateness of statistical tests used?**

**Yes, and I have assessed the statistics in my report.**

**Comments to author:**

m6A RNA methylation is one of the most common RNA modifications in prokaryotes and eukaryotes. However, the conservation and divergence of m6A distribution patterns and functions in plant virus disease are still not clear. In this manuscript, the authors analyzed m6A methylomes in rice plants infected with two devastating viruses. They found the m6A methylation levels were increased in rice infected with viruses, and the m6A methylation were mainly associated with genes that were not actively expressed in rice infected with viruses. They also identified several antiviral pathway-related genes. This study is interesting and contributes to our understanding of m6A modifications in interaction between plant and viruses.

Despite the findings presented, there are many major concerns which need further clarifications before the manuscript could be considered for publication (see below):

1. The purpose of this paper is to explore the conservation and divergence of m6A distribution patterns and functions in plant virus disease. However, there are NO any figures or texts of m6A distribution patterns in main figures and context! What is the m6A distribution in mock, and if it changes (increase or decrease) after infection? Are there any specific features that are closely related to expression pattern? Are they conserved or divergent to other known m6A patterns? Are they specific in plant virus disease? This is very important point and conclusion in this paper.

2. Line 145, "a total of 26,390, 27,038, and 26,675 unique m6A peaks with high confidence (p < 0.05, fold change > 1.5)", the author described these numbers three times (Line 169,228), don't have to mention it as summary three times! Also some related figure are repeats, please revise it.

3. The peaks from m6A-IP-seq include both plant and virus. The authors should add the data, how many peaks (%) are from plant, and how many from virus? The same analysis should be done on RNA-seq.

4. The main data that was missing in this paper is the genes corresponding to peaks! How many genes were identified as m6A marked genes which are corresponding to all peaks? How did they change after infection? Are m6A are closely related to these changes?

5. Line 177, "There were 8,010 and 6,602 unique differential m6A peaks for RBSDV- and RSV infected rice plants, respectively", since the authors identified 26,390, 27,037, and 26,674 peaks, how did they identify 8,010 and 6,602 unique peaks? Are these peaks are new after infection?

They author should clearly state that among these peaks, how many common peaks in both mock and virus-infected plants, how many new peaks in infected plants, then analyze how they change?

6. Line 241, "Rice m6A methylation modifications display sensitive, dynamic, and diverse responses to infection of rice plants by virus", based on the results in this part, the author cannot give this subtitle. How sensitive and how diverse?

7. Line 127, where is Figure 1B?

**Reviewer 2**

**Are you able to assess all statistics in the manuscript, including the appropriateness of statistical tests used?**

**Yes, and I have assessed the statistics in my report.**

**Comments to author:**

N6-methyladenosine (m6A) is the most abundant RNA post-transcriptional modification. m6A has been shown to regulate numerous biological processes in animals, the function of m6A and m6A machinery are relatively less understood in plants, especially in host-virus interaction. In this ms, the authors profiled the m6A map of the rice and two viruses using m6A-IP-seq, together with RNA-seq and qRT-PCR. The authors tried to reveal the relationship between the m6A modification and gene expression of the m6A machinery, antiviral RNA silencing pathway and antiviral phytohormone metabolic-related pathway.

Overall speaking, this is a piece of interesting work, and the idea is novel, and of broad interest in biology field. The results, if validated, would have significant impact in the host-microbe interaction. However, the manuscript at the current form has many flaws, some of which are very serious. They authors reported many observations/ relationships but many of such analyses and conclusions lack vigor; and need to be carefully re-examined and clarified.

Major points:

1. Logical flow of the manuscripts needs to be re-sorted. The current manuscript is very sloppy and there are so many mistakes in the writing; and it is impossible for the readers to understand the contents without substantial rewriting.

2. Many conclusions are not grounded and also inconsistent through the manuscript. For examples:

Line 196-197: "In contrast, a significant number of methylated genes were enriched in the low expression gene group (Fig. 5B and 5C). According to Fig. 5B and 5C, the number of unmethylated genes is more than the one of the methylated genes. How did the authors draw this conclusion?

In Fig. 5D, no matters in mock or infected rice, the methylated genes are more than unmethylated genes. But in line 200, the authors have a conclusion: Hence, m6A modification was associated with genes that were not expressed actively in rice infected with viruses. This reviewer thought that based on Fig. 5D, the result is not specific in infected rice.

3. In Fig. 8A, the increased m6A level in infected samples may come from m6A in viral RNAs. How did the authors exclude this possibility?

4. In the session titled "Correlation of m6A levels with expression of key genes involved in plant antiviral RNA silencing pathway and hormone metabolism". This reviewer did not find out whether the two genes were specifically selected based on their m6A modification in the m6A-IP-seq. Addition to this confusion is that Line 281-283: m6A-IP-qPCR was performed. What is the rationale for such a m6A-IP-qPCR? Also, the two genes, osAGO18 expression is increased, whereas the OsSLRL1 is reduced. How are the opposite patterns are related to m6A change? In addition, the m6A levels in position 12 of OsAGO18, not position 1 or other regions, was increased by approximately 13-fold and 11-fold in RBSDV- and RSV-infected samples, respectively, compared with mock-infected rice plants (Fig. 8E-F) what is the standard for significance here?

5. This reviewer cannot see any relationship between m6A and the expression of m6A machinery in Fig.9. For example, MTA3 and MTA4 are both methylated in RBSDV- and RSV-infected samples (line 321-322), but only in RSV-infected samples, MTA3 and MTA4 are up-regulated (Fig. 9A). In addition, the authors mentioned that YTH01, 10, 11 and 12 are all methylated in RBSDV-infected samples (line 324-325), but all of them do not change upon RBSDV infection. While YTH05 and YTH08 are methylated in RSV-infected samples, YTH05 is upregulated and YTH08 is downregulated in RSV-infected samples. Because their expression patterns were totally different and do not obey any rules, the conclusion regarding the relationship between m6A and the expression of m6A machinery is rocky.

6. In Fig. 10, OsAGO1c, OsAGO2, OsAGO18, and OsRDR1 were m6A methylated in RBSDV-infected rice, while only RDR1 and AGO18 were up-regulated. OsDCL1a, OsDCL3b, OsDCL4, OsAGO2, OsAGO12, OsAGO5c, OsAGO17, OsAGO18, OsRDR1, and OsRDR3 were methylated in RSV- infected rice, while only AGO5c and AGO12 were up-regulated. This reviewer cannot see any relation between m6A and expression of these core components in RNA silencing pathway. How could the authors get the conclusion that m6A involves in the antiviral RNA silencing pathway?

7. Many computational analyses need to be redone as well. What was exactly the peak-calling method for m6A? How the m6A-IP is normalized relative to the input? Any control IP? In Fig.3 A and B, majority reads of input are higher than those of the IP samples. Based on the peaks here, it is hard to distinguish true m6A peaks. The reviewer suggests to draw the calling peak instead of reads coverage.

8. Fig. 7: the most consensus motifs even do not have A? How does this come?

Minor points:

1. In line 174, the author calculated the correlation between the peak number and q-value. But in corresponding fig. 4B, the label is peak number and p-value. Which one is correct? In addition, please indicate what kind of p-value you used.

2. line 180: The median number of the peak enrichment-fold was approximately 25-27.

In the corresponding Fig. 4D, the label of y-axis is not consistent with 25-27 fold enrichment. Please check your calculation or label.

3. In Fig 4C, is y-axis number of genes or number of peaks?

4. The authors defined the high-expressed genes (FPKM ≥ 1) and genes which are not highly expressed (FPKM < 1) in line 193-194, but the authors further separated high-expressed genes into groups of FPKM = 1-5 and FPKM ≥ 5 in Fig. 5B without any explanation. It is quite confused when one reads line 192-200 regarding high- and low- expressed genes.

5. The authors mentioned to use mRNAs to perform LC-MS/MS and dot blot. What method did the authors use to isolate mRNAs? Please indicate in the Method section.

6. In Fig. 8B, please include a loading control to show the same amount of RNAs in Mock, RBSDV- and RSV-infected samples was loaded.

7. The authors mentioned that OsALKBH9B-2 was up-regulated in RSV-infected samples. Where is OsALKBH9B-2 in Fig. 9B?

8. There are lots of incorrect labeling in the figures. Please carefully check and correct them. For example, in Fig. 4D, according to your y-axis, some of the fold-enrichment reach unbelievable fold change (2^(2^8) fold). In Fig. 9 and 10, the authors did not mention what the meaning of a, b and c above the column is. These are only two examples, please check carefully through the whole ms.

9. Line 136: Samples of these series libraries came from mock-, RSV-,and RBSDV-infected rice leaves at 60 dpt. However, the most severe symptoms are in 20- and 60 dpt respectively. Thus, the sampling timings do not catch the best time for one viral infection.

10. Fig. 2 and 3: the better presentation way is needed: IP/Input.

11. M6A location: 5'UTR, Coding-sequence or 3'UTR? There are many conflicted statements through the ms.

Line 480: most m6A sites were located in the CDS region? What about in Fig. 6A?

**Authors Response**

**Point-by-point responses to the reviewers’ comments:**

Reviewer #1:

1. The purpose of this paper is to explore the conservation and divergence of m6A distribution patterns and functions in plant virus disease. However, there are NO any figures or texts of m6A distribution patterns in main figures and context! What is the m6A distribution in mock, and if it changes (increase or decrease) after infection? Are there any specific features that are closely related to expression pattern? Are they conserved or divergent to other known m6A patterns? Are they specific in plant virus disease? This is very important point and conclusion in this paper.

*Response: Thanks very much for your constructive and critical suggestions, your professional advices will improve the manuscript a lot. The responses to your questions are listed below one by one.*

(1) However, there are NO any figures or texts of m6A distribution patterns in main figures and context!

*Response: we described the m6A distribution pattern in the manuscript in Fig. 6A, Fig. S1, Fig. 2, Fig. 3, and Fig. 4. However, we did not give a conclusion sentence to summarize these results. We revised the sentences and given the conclusions to each part.*

*The reads distribution analysis (Fig. S1) showed that all the m6A-IP samples were highly enriched around the stop codon and within 3’-untranslated regions (3’-UTRs), which are in line with the previous reports in HIV-infected T-cells and maize and suggested the m6A-IP sequencing data are reliable. The revised sentence was showed as “Read distribution analysis showed that all the m6A-IP samples were highly enriched around the stop codon and within 3’-untranslated regions (3’-UTRs), which are in line with the previous reports in HIV-infected T-cells and maize and suggested the m6A-IP sequencing data are reliable and with a high authenticity [6, 15] (Supplemental Fig. S1)”.*

*We also mapped the m6A reads obtained by m6A-IP sequencing to the viral (Fig. 3) and rice reference genome (Fig. 2). In rice, we found that most of the m6A peaks were common in mock-, RBSDV-, and RSV-infected plant, but some m6A peaks were random distributed and different in RBSDV- and RSV-infected rice, and the common m6A peaks were mainly distributed in rice chromosomes 1, 2, and 3 (Fig. 2). Besides, some common m6A peaks were simultaneously and specifically distributed in chromosomes 4, 5, and 10 in RBSDV- and RSV-infected rice (Fig. 2). We already revised the sentence that description of the m6A distribution pattern in manuscript, which was showed as “Most of the peaks were common in mock-, RBSDV-, and RSV-infected rice, but some m6A peaks were random distributed and different in RBSDV- and RSV-infected rice, and the common m6A peaks were mainly distributed in rice chromosomes 1, 2, and 3 (Fig. 2). Besides, some common m6A peaks were simultaneously and specifically distributed in chromosomes 4, 5, and 10 in RBSDV- and RSV-infected rice (Fig. 2)”.*

*For RSV, we found that the main m6A clusters were located on the genome segment of RNA1, RNA2, and RNA3, and compared to the input, several clearly m6A peaks were in the RNA1 to RNA3, two m6A peaks located to the 3’ terminal of RNA1 (Fig. 3B and 3D, Red arrows).*

*For RBSDV, clusters of m6A peaks were clearly observed in the 5’ terminal of RBSDV genomic S1, S2, S3, S5, S6, S9, and S10, and some discrete peaks appeared in S4, and S7 (Fig. 3A and 3C, Red Arrows). we revised the sentences and conclusions, which were showed as “In particular, clusters of m6A peaks were clearly observed in the 5’ terminal of RBSDV genomic S1, S2, S3, S4, S5, S6, S9, and S10, and some discrete peaks appeared in S4, and S7 (Fig. 3A, Red Arrows). In RSV-infected sample, the main m6A peak clusters were located on the genomic RNA2, RNA3, and RNA4, and compared to the input, several clearly m6A peaks were in the RNA1 to RNA4, two m6A peaks located to the 3’ terminal of RNA1 (Fig. 3B, Red Arrows). We also exhibited the fine m6A peaks that distributed to the each viral genomic RNAs (Supplementary Table S6), and the viral specific m6A peaks that distributed on RBSDV S5, S6, S9 (Fig. 3C) and RSV RNA1, RNA2, and RNA3 (Fig. 3D) were selected and exhibited. Our results suggested that the m6A modification often occurred in the 5’-terminal of the genomic RNAs of RBSDV, while they were random distributed on RSV genome. These maybe resulted from the characteristic of the two completely different plant viruses (RSV is a single-strand RNA virus, while RBSDV is a double-stranded RNA virus)”.*

(2) What is the m6A distribution in mock, and if it changes (increase or decrease) after infection?

*Response: Thanks very much for your valuable remaining.*

*For the single transcript, m6A distribution pattern in mock were in line with samples that were infected with RBSDV and RSV (Fig. 6A, Fig. S1). The m6A peaks were enriched around the stop codon and within 3’-untranslated regions (3’-UTRs) of the transcripts. Our results were in accordance with previous research in HIV-infected T-cells of human beings and maize (Nature Microbiology, 2016, Fig.2; Plant Physiology, 2019, Fig. 1A). We have revised the sentence, which was showed as “Read distribution analysis showed that all the m6A-IP samples were highly enriched around the stop codon and within 3’-untranslated regions (3’-UTRs), which are in line with the previous reports in HIV-infected T-cells and maize and suggested the m6A-IP sequencing data are reliable and with a high authenticity [6, 15] (Supplemental Fig. S1)”.*

*In genomic level, the m6A peak distributions were showed in Fig. 2. The m6A peaks distribution density were highly consistent with the corresponding gene density on the same chromosome position. We revised the sentence that description of the m6A distribution pattern in the mock-treated sample, which were showed as “The gene density according to the previously reported data is presented in Fig. 2, and the m6A peak distribution density were highly consistent with the corresponding gene density on the same chromosome position in mock sample”.*

*In the m6A methylated peaks, the total number of m6A peaks or differential m6A peaks were also increased in rice infected with viruses compared to mock-treated sample, which was showed as “The findings clearly indicated that rice m6A RNA methylation was enriched under RSV and RBSDV infection. In terms of differential m6a peak number, the enriched peaks were also increased in rice infected with viruses (Fig. 4A, Supplementary Table S7)” and “There were 8,010 and 6,602 unique and different m6A peaks for RBSDV- and RSV-infected rice plants compared to mock-treated sample, respectively (Fig. 4C)”, in Supplementary Table S7, and Fig. 4A and 4C.*

*Further, we determined the m6A content in mock, RBSDV-, and RSV-infected rice samples through LC-MS/MS analysis, the m6A content of total mRNAs were greatly increased in both RBSDV- and RSV-infected sample compared to the mock-treated sample (Fig. 8A). Besides, we also measured the m6A methylated RNA levels in total mRNAs (including majority of plant mRNAs and microscale amount of viral RNAs) using the specific anti-m6A antibody (#ab208577; Abcam) by dot-blot, the results showed that the color intensity were evidently stronger in both RBSDV- and RSV-infected sample compared to the mock-treated sample (Fig. 8B). Taken together, these results showed that the m6A methylation level were significantly improved in virus-infected rice sample than mock-treated sample.*

(3) Are there any specific features that are closely related to expression pattern?

*Response: Thanks very much for your constructive and critical suggestion, your question was significant and was considered in our study firstly. We also want to find the regular pattern between m6A methylation and expression level of the target gene through integrated analyses of the m6A-IP sequencing date and the gene expression levels.*

*To answer the questions, we firstly used the genes that involved in biosynthesis and metabolic of the seven main phytohormone as analysis material. We found that 37 and 19 genes in RBSDV- and RSV-infected samples were methylated (Supplementary Fig. S10B, Supplementary Table S12) in total 154 genes whose expression level were determined. We divided the gene body to five parts (5’-UTR, CDS, 3’-UTR, 5’-UTR/CDS, and CDS/3’-UTR, the 5’-UTR/CDS and CDS/3’-UTR mean the m6A methylated reads that containing the start codon or stop codon, respectively), and the detail m6A methylated region on the target gene body were mapped and counted one by one (Fig. 10C). Then, we combined the qRT-PCR results of the target genes (Supplementary Fig. S3 to S9) with the location of m6A methylation region, and we found the target gene was down-regulated if the m6A region was located in the 5’-UTR (75%), CDS (50%), and CDS/3’-UTR (71%), with the location in the 3’-UTR often being associated with up-regulation (50%) of the target gene (Supplementary Fig. S10D).*

*In addition, to obtain the general regular pattern between m6A methylation and expression level of the target gene. That widely integrated analyses of the relationship between m6A methylation regions and expression levels using RNA-seq and m6A-IP-seq were performed (Supplementary Fig. S10E and 10F). we analyzed 3,734 and 3,336 m6A peaks in RBSDV- and RSV-infected plants, respectively (Supplementary Table S13). And we did not obtain the same regular pattern to the seven main phytohormone related genes. From the Supplementary Fig. S10E and 10F, we known that the expression levels of most m6A methylated genes were not changed in RSV-infected sample (Fig. S10F), whereas the expression levels of most m6A methylated genes were down-regulated in RBSDV-infected sample (Fig. S10E), and the m6A methylation were most occurred in CDS and 3’-UTR region in both RSV- and RBSDV-infected samples. In sum, these results indicated that the regulatory roles of post-transcriptional m6A modification differed upon RBSDV or RSV infection of rice plants, and that certain m6A sites on specific genes may have specific functions, the regular pattern between expression level and m6A methylation region, which suitable for all m6A methylated genes, have not been found so far. This may be due to the fact that difference virus cause plant disease through hijacking different host’s signal pathways, and different genes own different sequences characteristics. We have revised the sentences and conclusions, which was showed as “The common regular pattern between expression level and m6A methylation region, which are suitable for all m6A methylated genes, have not been found so far. This may be due to the fact that difference virus cause plant disease through hijacking different host’s signal pathways, and different genes own different sequences characteristics”.*

(4) Are they conserved or divergent to other known m6A patterns?

*Response: Thanks. The m6A distribution pattern in our study is conserved among human beings and plant (rice, and maize).*

*In rice callus and leaf, majority (around 90%) m6A peaks were with genic regions. Among them, more than 70% genic peaks were localized near CDSs and 3’-UTRs, where about 20% m6A peaks were found in intronic regions and 5’-UTRs (2014-RNA biology, Fig. 1A & Fig. 1B). In human beings, the m6A peaks that mapping to the CDS and 3’-UTR of human genome were 41% and 32% in HIV-infected T-cells, respectively, which account for majority of the total m6A peaks (2016-Nature Microbiology, Fig. 2B). In maize, m6A peaks in protein-coding genes were primarily enriched in the 3’-UTR (69.2%), and in the vicinity of the stop codon (20.4%), while less present in CDS (4.7%) (2019-Plant Physiology, Fig. 1C & 1D).*

*Taken together, the m6A distribution pattern in mock- and virus-infected samples were in line with previous studies in rice, human beings, and maize. Hence, the m6A distribution pattern in our study was conserved to other known m6A patterns. We already revised the sentence that description of the m6A distribution pattern in the manuscript, which was showed as “Read distribution analysis showed that all the m6A-IP samples were highly enriched around the stop codon and within 3’-untranslated regions (3’-UTRs), which are in line with the previous reports in HIV-infected T-cells and maize and suggested the m6A-IP sequencing data are reliable and with a high authenticity [6, 15] (Supplemental Fig. S1)”.*

*Reference:*

*[1]. Lichinchi G, Gao S, Saletore Y, Gonzalez GM, Bansal V, Wang Y, Mason CE, Rana TM: Dynamics of the human and viral m6A RNA methylomes during HIV-1 infection of T cells. Nature Microbiology 2016, 1:16011.*

*[2]. Luo JH, Wang Y, Wang M, Zhang LY, Peng HR, Zhou YY, Jia GF, He Y: Natural variation in RNA m6A methylation and its relationship with translational status. Plant Physiology 2019, 182:332-344.*

*[3]. Li Y, Wang X, Li C, Hu S, Yu J, Song S: Transcriptome-wide N6-methyladenosine profiling of rice callus and leaf reveals the presence of tissue-specific competitors involved in selective mRNA modification. RNA Biology 2014, 11:1180-1188.*

(5) Are they specific in plant virus disease?

*Response: Thank you very much for your attention. Actually, our study was the first case that reporting of the m6A methylation in plant-virus interactions. The m6A distribution pattern in plant virus (Fig. 3, Supplementary Table S6), especially in our investigated two representative rice viruses (RSV & RBSDV), was specific and novel. We already revised the sentences that description of the m6A distribution on viral genomic RNAs, which was showed as “The clear reads obtained by NGS were also aligned to the reference RBSDV and RSV genomic RNAs, and the m6A peaks spanning the full sequences of different segments of viruses were mapped (Fig. 3, Supplementary Table S6). In particular, clusters of m6A peaks were clearly observed in the 5’ terminal of RBSDV genomic S1, S2, S3, S4, S5, S6, S9, and S10, and some discrete peaks appeared in S4, and S7 (Fig. 3A, Red Arrows). In RSV-infected sample, the main m6A peak clusters were located on the genomic RNA2, RNA3, and RNA4, and compared to the input, several clearly m6A peaks were in the RNA1 to RNA4, two m6A peaks located to the 3’ terminal of RNA1 (Fig. 3B, Red Arrows). We also exhibited the fine m6A peaks that distributed to the each viral genomic RNAs (Supplementary Table S6), and the viral specific m6A peaks that distributed on RBSDV S5, S6, S9 (Fig. 3C) and RSV RNA1, RNA2, and RNA3 (Fig. 3D) were selected and exhibited. Our results suggested that the m6A modification often occurred in the 5’-terminal of the genomic RNAs of RBSDV, while they were random distributed on RSV genome. These maybe resulted from the characteristic of the two completely different plant viruses (RSV is a single-strand RNA virus, while RBSDV is a double-stranded RNA virus). Taken together, in addition to the mRNA of the host plant, viral mRNAs could also be N6-methyladenosine methylated under interactions between virus and rice, and the m6A distribution pattern on viral genomic RNA were specific and novel”.*

*To our knowledge, the m6A methylation was simply investigated in CMV/TNV infected Arabidopsis (2017-PNAS), TMV-infected tobacco (2018- Virology Journal), and Pyricularia oryzae infected rice (2018-FEMS Microbiology Letter), previously. However, the N6-adenosine-methyltransferase were widely investigated in these studies, and the m6A distributions patterns associated with host genomic or pathogens’ genomic were not explored so far. Therefore, our investigation was the first case that description of the m6A distribution pastern in both viral genome and host genome, and is specific and novel.*

*Reference:*

*[1]. M M-P, Aparicio F, MP L-G, JM B, JA S-N, V P: Arabidopsis m6A demethylase activity modulates viral infection of a plant virus and the m6A abundance in its genomic RNAs. Proc Natl Acad Sci U S A 2017, 114:10755.*

*[2]. Li Z, Shi J, Yu L, Zhao X, Ran L, Hu D, Song B: N6-methyl-adenosine level in Nicotiana tabacum is associated with tobacco mosaic virus. Virol J 2018, 15:87.*

*[3]. Shi Y K, Wang H, Wang J, Liu X H, Lin F C, Lu J P: N6-methyladenosine RNA methylation is involved in virulence of the rice blast fungus Pyricularia oryzae (syn. Magaporthe oryzae). FEMS Microbiology Letter 2018, 366:1.*

2. Line 145, "a total of 26,390, 27,038, and 26,675 unique m6A peaks with high confidence (p < 0.05, fold change > 1.5)", the author described these numbers three times (Line 169,228), don't have to mention it as summary three times! Also some related figure are repeats, please revise it.

*Response: Thanks very much for your profession and carefulness. We have already revised the mentioned three sentences that description of the unique m6A peaks. We revised the first sentences to “The m6A-IP-seq analyses detected more than 2,6000 m6A peaks in each individual treatment and biological replicate”, the second sentence was not changed, while the third sentence was revised to “Collectively, there were 15,977, 16,854, and 16,267 m6A methylated genes that corresponding to mock-, RBSDV-, and RSV-infected rice, respectively” in line 186-187. Thanks very much for your carefulness again.*

3. The peaks from m6A-IP-seq include both plant and virus. The authors should add the data, how many peaks (%) are from plant, and how many from virus? The same analysis should be done on RNA-seq.

*Response: Thanks very much for your constructive suggestions. Your concern is very important and essential for quality control of our m6A-IP sequencing experiment. The detail reads number of m6A-IP sequencing reads and input RNA-seq data were exhibited in Supplementary Table S4. We have revised the table, and added the mapping information of the viral reads.*

*In m6A-IP sequencing data, the obtained average clear reads were 103,786,857, 104,773,437, and 107,543,039 in mock-, RSV-, and RBSDV-infected rice samples, respectively. The average mapping rate to rice genome of the clear reads were 58.19%, 53.93%, and 55.37% in mock-, RSV-, and RBSDV-infected rice samples, respectively. The average of total reads number that mapped to RSV and RBSDV genomic RNA were 52,386,718, and 53,771,519, respectively, and the reads number were 0.02%, and 1.07%.*

*In input RNA-seq data, the obtained average clear reads were 114,123,410, 167,200,976, and 151,023,967 in mock-, RSV-, and RBSDV-infected rice samples, respectively. The average mapping rate to rice genome of the clear reads were 98.44%, 58.08%, and 96.92% in mock-, RSV-, and RBSDV-infected rice samples, respectively. Hence, the mapping rate to the two viral genome RNAs were about 32.04% and 1.47% for RSV and RBSDV in input RNA-seq data, respectively. The results suggested that the RBSDV viral titer was lower than the RSV in collected rice sample that were used for sequencing analyses in our manuscript.*

*The detail ratio and reads number were supplied in Supplementary Table S4. Thanks very much for your valuable suggestions again.*

4. The main data that was missing in this paper is the genes corresponding to peaks! How many genes were identified as m6A marked genes which are corresponding to all peaks? How did they change after infection? Are m6A are closely related to these changes?

*Response: Thanks very much for your profession and carefulness. Your mentioned that the gene information corresponding to the m6A peaks was missing. Yes, we checked the manuscript and find that* it *was actually a mistake caused by our careless. I am sorry for my careless in preparation of the manuscript. Our responses to your questions are listed one by one and showed below:*

(1) How many genes were identified as m6A marked genes which are corresponding to all peaks?

*Response: Thanks a lot, consequently, a total of 26,390, 27,038, and 26,675 unique m6A peaks with high confidence (p < 0.05, fold change > 1.5) for mock-, RBSDV-, and RSV-infected samples were detected, respectively (Fig. 4A). These peaks were aligned to the rice genomic DNA, and obtained 15,977, 16,845, and 16,267 methylated genes for mock-, RBSDV-, and RSV-infected samples, respectively (Supplementary Table S2). These results indicated that the m6A methylation was activated in viruses’ infected rice samples, the unique peak number and gene number were simultaneously increased. Further, compared to the mock-treated rice, there are 8,011 and 6,603 differential regulated peaks in RBSDV- and RSV-infected samples, respectively (Fig. 4C).*

(2) How did they change after infection? Are m6A are closely related to these changes?

*Response: The results of Fig. 4 and Supplementary Table S2 demonstrated that the host m6A methylation was activated upon viruses’ infection, and the m6A methylated genes number were increased in RBSDV- and RSV-infected samples, respectively (Fig. 4C). Further analyses of the expression level of these m6A methylated genes by combination with the transcriptome data, the genes were divided into highly expressed genes (FRKM ≥ 1) and genes that were not highly expressed (FRKM < 1). The expression of the genes that was high or low were showed in mock-, RBSDV-, and RSV-infected samples (Fig. 5B), and most methylated genes were enriched in low expression gene group (Fig. 5B and 5C). Hence, m6A modification was associated with genes that were not expressed actively in rice infected with viruses.*

*In addition, the m6A methylated genes were involved in multiple molecular functions, particularly in binding and catalytic activity through Gene Ontology (GO) analyses (Fig. 6B). Further analyses of the different m6A modification-related genes by Kyoto Encyclopedia of Genes and Genomes (KEGG) analyses, the carbohydrate metabolism, translation, protein folding/sorting/degradation, amino acid metabolism, and signal transduction pathways related genes were significantly enriched (Fig. 6C), these results suggested that m6A modification widely involved in and closely related to the intracellular carbohydrate metabolism, amino acid metabolism, and protein translation/folding/sorting/degradation in RSV- and RBSDV- infected rice sample. We have revised the sentence.*

*Thanks very much for your critical and valuable suggestions again.*

5. Line 177, "There were 8,010 and 6,602 unique differential m6A peaks for RBSDV- and RSV infected rice plants, respectively", since the authors identified 26,390, 27,037, and 26,674 peaks, how did they identify 8,010 and 6,602 unique peaks? Are these peaks are new after infection? They author should clearly state that among these peaks, how many common peaks in both mock and virus-infected plants, how many new peaks in infected plants, then analyze how they change?

*Response: Thanks very much for your careless and profession. From the m6A-IP-sequnceing data, 8,010 and 6,602 differential m6A peaks were identified in RBSDV- and RSV infected rice plants, respectively. Here, we used the peak calling method with software that described in method section, and the data were processed by the company of oebiotech that located in Shanghai. Among these peaks, there are 3,897 and 2,900 new peaks appeared upon RBSDV and RSV infection, and 4113, and 3702 common peaks in RBSDV- and RSV-infected sample compared with mock-treated rice, and 1503 differential m6A peaks were both appeared in RBSDV- and RSV-infected sample. These data were supplied in Supplementary Table S19. From the table, we known that 48.7% and 43.9% differential m6A peaks were newly appeared* *upon RBSDV- and RSV infection of rice, and the change of about half the total differential m6A peaks suggested that m6A methylation were tightly associated with viruses’ infection of plant. We have already revised the sentence that description of the results, which was showed as “Among these differential m6A peaks, there are 3,897 and 2,900 new peaks appeared upon RBSDV and RSV infection, and 4113, and 3702 common peaks in RBSDV- and RSV-infected sample compared with mock-treated rice, and 1503 differential m6A peaks were both appeared in RBSDV- and RSV-infected sample (Supplementary Table S19). These results suggested that 48.7% and 43.9% differential m6A peaks were newly appeared upon RBSDV- and RSV infection of rice, respectively, which also indicated that m6A methylation were tightly associated with viruses’ infection of plant.”.*

*Thanks very much for your critical suggestions again.*

6. Line 241, "Rice m6A methylation modifications display sensitive, dynamic, and diverse responses to infection of rice plants by virus", based on the results in this part, the author cannot give this subtitle. How sensitive and how diverse?

*Response: Thanks very much for your critical suggestions. We completely accepted your advices, and revised the subtitle to “Rice m6A methylation modifications display sensitive and dynamic responses to infection of rice plants by virus”, which was showed in line 290-296. We carefully described the change profile of m6A contents upon viruses’ infection from 0 to 16 days, and found that the m6A methylation was significantly changed just at 1st day post virus infection. Responses of plant m6A methylation to virus infection were sensitive and rapid. Hence, we conclude that rice m6A methylation modifications display sensitive and dynamic upon viruses’ infection of rice plant. Thanks very much for your critical suggestions again.*

7. Line 127, where is Figure 1B?

*Response: Thanks very much for your profession and carefulness. It’s our careless. We forget to addition the Fig. 1B in the behind the corresponding contents of the manuscript, we already have revised it, which was showed as “Leaves were collected from these symptomatic rice plants for RT-PCR and western blotting analyses to detect the target viruses (Fig. 1B and 1C)” and “These results and those of western blots using RSV NS3 and RBSDV p10 antisera (Fig. 1B and 1C) confirmed the independent infection of the plants by the viruses following inoculation”. Thanks very much for your critical suggestion and carefulness again, your help will be highly appreciated.*

Reviewer #2:

Major points:

1. Logical flow of the manuscripts needs to be re-sorted. The current manuscript is very sloppy and there are so many mistakes in the writing; and it is impossible for the readers to understand the contents without substantial rewriting.

*Response: Thanks very much for your constructive suggestion. According to your suggestions, we have revised the writing, figures, and supplementary files one by one. we checked the manuscript throughout, and re-examined the labeling of the figures.*

*Thanks very much for your critical suggestions, and your help will greatly improve our manuscript.*

2. Many conclusions are not grounded and also inconsistent through the manuscript. For examples:

Line 196-197: "In contrast, a significant number of methylated genes were enriched in the low expression gene group (Fig. 5B and 5C). According to Fig. 5B and 5C, the number of unmethylated genes is more than the one of the methylated genes. How did the authors draw this conclusion?

In Fig. 5D, no matters in mock or infected rice, the methylated genes are more than unmethylated genes. But in line 200, the authors have a conclusion: Hence, m6A modification was associated with genes that were not expressed actively in rice infected with viruses. This reviewer thought that based on Fig. 5D, the result is not specific in infected rice.

*Response: Thanks very much for your constructive and valuable suggestions. Responses to your question were listed as follows.*

*Besides, we also checked the results descriptions of other figures in the manuscript one by one, and checked whether other results descriptions are consistent and grounded.*

(1) Line 196-197: "In contrast, a significant number of methylated genes were enriched in the low expression gene group (Fig. 5B and 5C). According to Fig. 5B and 5C, the number of unmethylated genes is more than the one of the methylated genes. How did the authors draw this conclusion?

*Response: Thanks very much for your suggestions. To deal with these problems, we revised the sentences that description of the Fig. 5B, Fig. 5C, and Fig. 5D. we also remarked the Fig. 5C, and showed the significance difference of the certain comparisons. The revisions were showed as “The findings suggested that the experiments were reproducible between the two replicates. Based on the confident sequencing results, the m6A peaks of each treatment were annotated to the genes. Rice genes that could be annotated by the m6A peaks were called m6A gene, and that couldn’t be annotated were called non-m6A gene in the next analyses. According to the Fragments Per Kilobase of exon model per Million mapped fragments (FPKM) (Supplementary Table S7), these genes were divided to three groups (FPKM < 1, 1 < FPKM < 5, and FPKM > 5), and the ratio of gene number of each category were calculated and showed by the heatmap (Fig. 5B). Either in the mock-treated sample or the viruses’ infected rice, most of the analysed genes (m6A or non-m6A genes) were mainly distributed in the low expressed category (FRKM < 1) (Fig. 5B). Compared to the mock-treated sample, the ratio of m6A methylated genes were increased in low expressed category of RSV- and RBSDV-infected samples (Fig. 5B). When these genes (m6A and non-m6A) were divided into highly expressed genes (FRKM ≥ 1) and genes that were not highly expressed (FRKM < 1), the expression of genes that was high or low were showed in mock-, RBSDV-, and RSV-infected samples (Fig. 5C), and we found that most genes were non-methylated and distributed in low expression category in mock-, RBSDV-, and RSV-infected rice samples (Fig. 5C). The m6A methylated genes were slightly enriched in the low expression category upon viruses’ infection of rice compared to mock-treated sample (Fig. 5C). Either in mock-treated or in RSV-infected and RBSDV-infected samples, the m6A-methylated genes were faintly expressed at a higher level than those of non-m6A methylated genes (Fig. 5D), and the highly expressed genes displayed a lower m6A occupancy (Fig. 5B).”, and the remarked Fig. 5C was supplied in newly submitted PPT that containing the whole figures of the manuscript.*

(2) In Fig. 5D, no matters in mock or infected rice, the methylated genes are more than unmethylated genes. But in line 200, the authors have a conclusion: Hence, m6A modification was associated with genes that were not expressed actively in rice infected with viruses. This reviewer thought that based on Fig. 5D, the result is not specific in infected rice.

*Response: Thanks a lot. Either in the mock-treated sample or the viruses’ infected rice, most of the analyzed genes (m6A or non-m6A genes) were mainly distributed in the low expressed category (FRKM < 1) (Fig. 5B, 5C, and 5D). However, compared to the mock- treated sample, the ratio of m6A methylated genes were increased in low expressed category of RSV- and RBSDV-infected samples (Fig. 5B). In Fig. 5C and 5D, we also found that the m6A methylated genes were enriched in the low expression category upon viruses’ infection of rice compared to mock-treated sample.*

*Based on these observations above, we concluded that m6A modification was associated with genes that were not actively expressed in rice infected with viruses. We already have revised the sentence that description of the results of Figure 5. The remarked Fig. 5C was supplied in newly submitted PPT that containing the whole figures of the manuscript.*

3. In Fig. 8A, the increased m6A level in infected samples may come from m6A in viral RNAs. How did the authors exclude this possibility?

*Response: Thanks very much for your critical suggestion. The Fig.8A and 8B was done using the purified total tissue mRNAs. So, the increased m6A level in infected samples was mainly caused by the methylation of host mRNA. Due to the viruses’ genomic RNA have no poly(A) sequences, and there are trace amounts of contaminations using the Ambion® Poly(A)Purist™ MAG kit (InvitrogenTM, USA), the purified total mRNA that used for dot-blot and LC-MS/MS analyses were most are plant mRNAs. While the RNA samples for m6A-IP-sequencing analyses were purified by Ribosome RNA (rRNA) depleted samples using the RiboMinus™ Plant Kit (Thermo Fisher Scientific, USA), and the viral genome were not deleted in analyses. The m6A-IP-Sequencing data showed that the percentage of the mapped reads to viral genomic RNAs was about 1.97% and 3.26%, respectively (Supplementary Table S4). Hence, in viral genomic un-deleted condition, the percentage was low. Whereas the samples used in experiment of Fig. 8A and 8B were viral genomic deleted samples, the possibility of the increased m6A level in infected samples may come from m6A in viral RNAs was very low.*

*The Fig. 8A and 8B showed that the total m6A contents and m6A methylated mRNAs were increased with viruses’ infection of rice. Besides, we further selected two candidate genes (OsAGO18 and OsSLRL1), and m6A-IP-qPCR was performed. We found that the m6A methylation levels that corresponding to the specific region of gene body (position 6 and 12 to OsAGO18, and position 2, 4, and 3 to OsSLRL1) were increased with the extension of infection time. Hence, we concluded that viruses’ infection activated the m6A methylation of the rice plant (Virus cannot methylate itself, all methylation modifications on RNAs were done by host).*

*Although we did not perform any experiment that determine the m6A levels of viral RNAs with the extension of viruses’ infection, and could not completely rule out the possibility that viral RNAs contributed to the increased m6A level in our analyses. We could conclude that the infection of plants with viruses markedly increased the N6-methyladenosine level of plant mRNAs, and the N6-methyladenosine level was positively correlated with virus infection of rice plants.*

*Thanks very much for your valuable suggestion again.*

4. In the session titled "Correlation of m6A levels with expression of key genes involved in plant antiviral RNA silencing pathway and hormone metabolism". This reviewer did not find out whether the two genes were specifically selected based on their m6A modification in the m6A-IP-seq. Addition to this confusion is that Line 281-283: m6A-IP-qPCR was performed. What is the rationale for such a m6A-IP-qPCR? Also, the two genes, osAGO18 expression is increased, whereas the OsSLRL1 is reduced. How are the opposite patterns are related to m6A change? In addition, the m6A levels in position 12 of OsAGO18, not position 1 or other regions, was increased by approximately 13-fold and 11-fold in RBSDV- and RSV-infected samples, respectively, compared with mock-infected rice plants (Fig. 8E-F) what is the standard for significance here?

*Response: Thanks very much for your constructive and valuable suggestions. Responses that corresponding to your questions were listed one by one, as follows.*

(1) This reviewer did not find out whether the two genes were specifically selected based on their m6A modification in the m6A-IP-seq.

*Response: Thanks very much for your constructive suggestion. The selected two candidate genes, which including OsAGO18 (Os07g0471300) and OsSLRL1 (Os01g0646300), were both differential m6A methylated genes in RBSDV- and RSV-infected rice samples compared to mock-treated rice (Supplementary Table S15 and S16). As described in manuscript, OsAGO18 was reported to participate in anti-virus RNA silencing pathways in rice by binding small interfering RNAs (siRNAs) (2015-eLife). The expression of OsAGO18 can be activated by jasmonate (JA) signalling (2020-Cell Host Microbe). OsSLRL1 is a DELLA family protein that regulates plant hormone metabolism and mediates the growth and development of plants (2018-Front Plant Sci; 2016-Plant Physiol; 2020-Plant Physiol).*

*As two essential components of major antiviral pathways (RNA silencing and phytohormone-mediated basic resistance), validation of their m6A methylation levels could evidence the credibility of our m6A-IP-sequenceing result, and demonstrated that the m6A also occurred in genes that directly function in RNA silencing and phytohormone-mediated basic resistance pathways.*

*Reference:*

*[1]. Wu J, Yang Z, Wang Y, Zheng L, Ye R, Ji Y, Zhao S, Ji S, Liu R, Xu L, et al: Viral-inducible Argonaute18 confers broad-spectrum virus resistance in rice by sequestering a host microRNA. eLife 2015, 4:e05733.*

*[2]. Yang Z, Huang Y, Yang J, Yao S, Zhao K, Wang D, Qin Q, Bian Z, Li Y, Lan Y, et al: Jasmonate signaling enhances RNA silencing and antiviral defense in rice. Cell Host Microbe 2020, 28:89-103.e108.*

*[3]. Um TY, Lee HY, Lee S, Chang SH, Chung PJ, Oh KB, Kim JK, Jang G, Choi YD: Jasmonate Zim-domain protein 9 interacts with slender rice 1 to mediate the antagonistic interaction between jasmonic and gibberellic acid signals in rice. Front Plant Sci 2018, 9:1866.*

*[4]. De Vleesschauwer D, Seifi HS, Filipe O, Haeck A, Huu SN, Demeestere K, Höfte M: The DELLA protein SLR1 integrates andamplifies salicylic acid- and jasmonic acid-dependent innate immunity in rice. Plant Physiol 2016, 170:1831-1847.*

*[5]. Mo W, Tang W, Du Y, Jing Y, Bu Q, Lin R: PHYTOCHROME-INTERACTING FACTOR-LIKE14 and SLENDER RICE1 interaction controls seedling growth under salt stress. Plant Physiol 2020, 184:506-517.*

(2) Addition to this confusion is that Line 281-283: m6A-IP-qPCR was performed. What is the rationale for such a m6A-IP-qPCR?

*Response: Thanks very much for your suggestion. m6A-IP-qPCR, also is called MeRIP-qPCR. It is a quantitative analyses of m6A methylated gene fragments. Briefly, the N6-methyladenosine could be recognized and precipitated by the anti-m6A specific antibody (#ab98712; Abcam), then the relative expression level of target m6A methylated gene in total m6A methylated gene fragments could be determined by the qRT-PCR.*

*In the manuscript, we firstly determined the relative expression level of target genes (OsAGO18 & OsSLRL1) (Fig. 8C and 8D), then the m6A-IP-qPCR was performed (Fig. 8E and 8F). There are two reasons for m6A-IP-qPCR analyses, first is to further evidence the m6A-IP-sequencing results, and the second is to explore the detail m6A methylation region on target gene body, then try to conclusion the relationship between the m6A methylation modification and the relative expression levels for the target gene.*

*Thanks very much for your constructive suggestion again.*

(3) Also, the two genes, osAGO18 expression is increased, whereas the OsSLRL1 is reduced. How are the opposite patterns are related to m6A change?

*Response: Thanks very much for your valuable and professional suggestion. Yes, although the two selected candidate genes (OsAGO18 & OsSLRL1) were both m6A methylated upon viruses’ infection, whereas the relative expression pattern of OsAGO18 (Increased) and OsSLRL1 (Decreased) were completely opposite to each other. It is undeniable that changes in the relative expression of OsAGO18 and OsSLRL1 were tightly correlated with the changes in their m6A modification level. The detail m6A methylation region on target gene body was essential for their functions, and different m6A methylation sites often have different effects to the expression pattern.*

*Through m6A-IP-qPCR analyses, we could certain two things: validation the credibility of the m6A-IP-sequencing results, and demonstration the association of the m6A methylation with the relative expression of target gene.*

*Thanks very much for your valuable suggestion.*

(4) In addition, the m6A levels in position 12 of OsAGO18, not position 1 or other regions, was increased by approximately 13-fold and 11-fold in RBSDV- and RSV-infected samples, respectively, compared with mock-infected rice plants (Fig. 8E-F) what is the standard for significance here?

*Response: Thanks very much for your carefulness and profession. We already added the detail calculation method in the method section “Gene expression quantification”, which was showed in line 969-978. The results were performed statistical analyses by SPSS software (version 22.0, IBM) and analyzed by one-way analysis of variance (ANOVA). We already labeled the column with different letters in Fig. 8E and 8F. Different letters above bars denote statistically significant differences according to the Duncan's multiple range test (p < 0.05).*

*Thanks very much for your critical suggestion.*

5. This reviewer cannot see any relationship between m6A and the expression of m6A machinery in Fig.9. For example, MTA3 and MTA4 are both methylated in RBSDV- and RSV-infected samples (line 321-322), but only in RSV-infected samples, MTA3 and MTA4 are up-regulated (Fig. 9A). In addition, the authors mentioned that YTH01, 10, 11 and 12 are all methylated in RBSDV-infected samples (line 324-325), but all of them do not change upon RBSDV infection. While YTH05 and YTH08 are methylated in RSV-infected samples, YTH05 is upregulated and YTH08 is downregulated in RSV-infected samples. Because their expression patterns were totally different and do not obey any rules, the conclusion regarding the relationship between m6A and the expression of m6A machinery is rocky.

*Response: Thanks very much for your valuable suggestions, and your professional spirits. Yes, your concerns are essential for our manuscript improving. Actually, we want to find the common regular pattern between m6A methylation and the expression profiles of target gene. Unfortunately, we failed and couldn’t found the common regular pattern. This is due to different m6A methylation site on gene bodies will exert different effect to the target gene, and probably caused the up-regulation, down-regulation, or no-change of the target genes. As you expected, we have revised the conclusion of Fig. 9, and showed as “In summary, the m6A modifications occurred in the genes that encoding the plant m6A methylation machinery, and probably regulated the dynamics of the target gene expression, which may act as a main post-translational gene expression regulation strategy under plant virus infection” and “The relationship between the relative expression level of target genes and m6A modification sites was counted by combination analyses (Supplementary Table S10), and no any fixed regular pattern has been found”.*

*Thanks very much for your constructive suggestions again and your help will be highly appreciated.*

6. In Fig. 10, OsAGO1c, OsAGO2, OsAGO18, and OsRDR1 were m6A methylated in RBSDV-infected rice, while only RDR1 and AGO18 were up-regulated. OsDCL1a, OsDCL3b, OsDCL4, OsAGO2, OsAGO12, OsAGO5c, OsAGO17, OsAGO18, OsRDR1, and OsRDR3 were methylated in RSV- infected rice, while only AGO5c and AGO12 were up-regulated. This reviewer cannot see any relation between m6A and expression of these core components in RNA silencing pathway. How could the authors get the conclusion that m6A involves in the antiviral RNA silencing pathway?

*Response: Thanks very much for your critical suggestions. Yes, we didn’t find any common regular pattern between m6A and expression of these core components in RNA silencing pathway. However, our experiment validated the real methylation of OsAGO18 (Representative component of RNA silencing pathway), and its expression pattern (up-regulation) was tightly associated with the m6A methylation levels upon viruses’ infections (Fig. 8E). In Fig. 10, we further analyzed the genes of RNA silencing pathway, and want to explore the common regular pattern, similarly to Fig. 9, we failed. Hence, we have revised the conclusions. Which was showed as “These results indicated the possible involvement of m6A methylation in the main antiviral RNA silencing pathways. The methylation may act as a fine regulator to mediate the spatial and temporal expression of target genes in arm race of plant-virus interaction”.*

*Thanks very much for your constructive suggestions, your help will be highly appreciated.*

7. Many computational analyses need to be redone as well. What was exactly the peak-calling method for m6A? How the m6A-IP is normalized relative to the input? Any control IP? In Fig. 3A and B, majority reads of input are higher than those of the IP samples. Based on the peaks here, it is hard to distinguish true m6A peaks. The reviewer suggests to draw the calling peak instead of reads coverage.

*Response：Thanks very much for your critical suggestions. We revised the corresponding sites in our manuscripts according to your constructive suggestions, and many computational analyses were redone as well. Responses for your questions were listed as follow:*

(1) What was exactly the peak-calling method for m6A?

*Response: Thanks a lot. The m6A peak calling method were performed by the MeTDiff peak calling software, and the annotation were done by ChIPseeker. We already added theses method in method section, and added the subtitle “Peak calling analysis”, which was showed as “Peak calling analysis.*

*The obtained m6A-seq clear reads of mock-treated, RSV-, and RBSDV-infected rice samples were aligned to their respective reference genome as described above. The m6A enriched peaks in each m6A-IP sample were determined by MeTDiff peak calling software [107] with corresponding input sample serving as control. The running options of the MeTDiff software were set as: FRAGMENT_LENGTH = 200, PEAK_CUTOFF_PVALUE = 0.01, PEAKS_CUTOFF_FDR = 0.05. After the running, the m6A peak were detected, and the called peaks were annotated by intersection with gene architecture by ChIPseeker [108]”.*

*Thanks very much for your profession again.*

(2) How the m6A-IP is normalized relative to the input? Any control IP?

*Response: Detection of m6A sites, or peak calling in m6A-IP-Seq requires evaluation of read enrichment in IP samples relative to control samples and detection of sites spanning multiple exon-exon junctions. Moreover, differential site detection requires comparing IP/control read enrichments of m6A-IP-Seq samples from two conditions. The m6A peak calling for the m6A-seq data was computed by the HEPeak package based on a Hidden Markov Model (HMM)-based Exome Peak-finding algorithm (2015-BMC Genomics). HEPeak models the correlation between continuous bins in an m6A peak region and it is a model-based approach, which admits rigorous statistical inference.*

*Reference:*

*[1]. Cui X, Meng J, Rao MK, Chen Y, Huang Y. HEPeak: an HMM-based exome peak-finding package for RNA epigenome sequencing data. BMC Genomics. 2015;16 Suppl 4: S2. doi:10.1186/1471-2164-16-S4-S2*

(3) In Fig. 3A and B, majority reads of input are higher than those of the IP samples. Based on the peaks here, it is hard to distinguish true m6A peaks. The reviewer suggests to draw the calling peak instead of reads coverage.

*Response: Thanks very much for your constructive suggestion, your valuable comments will greatly improve the manuscript. We already fully accepted your suggestions, and re-exhibited the fine m6A peak distributions on two viral genomic RNAs in Fig. 3C, Fig. 3D, and Supplementary Table S6.*

*The following content was added in the manuscript. “We also exhibited the fine m6A peaks that distributed to the each viral genomic RNAs (Supplementary Table S6), and the viral specific m6A peaks that distributed on RBSDV S5, S6, S9 (Fig. 3C) and RSV RNA1, RNA2, and RNA3 (Fig. 3D) were selected and exhibited. Our results suggested that the m6A modification often occurred in the 5’-terminal of the genomic RNAs of RBSDV, while they were random distributed on RSV genome. These maybe resulted from the characteristic of the two completely different plant viruses (RSV is a single-strand RNA virus, while RBSDV is a double-stranded RNA virus).”*

*Thanks very much for your critical suggestions again.*

8. Fig. 7: the most consensus motifs even do not have A? How does this come?

*Response: Thanks very much for your constructive and valuable suggestion. The consensus we exhibited in the Fig. 7 was the most common consensus among the reads of m6A-IP. We used the MEME (http://meme-suite.org/tools/dreme) and DREME tools suite to explore the enhancement analyses. The detail searching parameters were described in subtitle “Identification of enriched motifs within m6A peaks”, which was showed as “The MEME (http://meme-suite.org/tools/dreme) [109] and Discriminative Regular Expression Motif Elicitation (DREME) [110] tools suites located in the MEME suite were used to perform motif enhancement analyses. The DREME suite was used to discover short (≤ 8 bp) and ungapped motifs enriched within series target m6A peak sequences relative to control sequences (shuffled m6A peak sequences). The series target m6A peak sequences were extracted from the rice reference genome (O. sativa. IRGSP-1.0) using the fastaFromBed function in BEDTools software v2.28 [111]. The control sequences were randomly produced by shuffling each m6A peak sequence while retaining the nucleotide frequencies. The performance was executed by “fasta-shuffle-letters” utility (k = 1) in the MEME suite. These two aforementioned types of sequences were loaded into DREME for discovery motifs. The parameter settings were as follows: meme-minw, 8; meme-maxw 30; meme-nmotifs, 10; E dreme e-value threshold, 0.05; Fimo-skip-spamo-skip-centrimo-score, 5.0; centrimo-ethresh, 10.0; The AME tools (http://meme-suite.org/tools/ame) package in the MEME suite was used to calculate the significance level of relative enrichment of one special motif within target sequences relative to the control sequences”.*

*The enriched consensuses were distributed around the methylated adenosine, not the consensus that containing the target ‘A’. Most of the publications focused on the analyses the common consensus that containing the methylated site (A), and compared the difference species (2019- PLoS Genet; 2020a-Plant Physiol; 2020b-Plant Physiol; 2020-eLife; 2014-RNA Biology). We also searched the already investigated common consensus, and analyzed their occurrence in our study. The results were showed in Supplementary Table S14, we analyzed the four most common consensus “RRACH”, “URUAY”, “RAGRAG”, and “UGUAMM”, and their percentage in total confident peaks were counted and listed. The consensus “RRACH” was most distributed (About 68%), and then “URUAY” (28%), then “RAGRAG” (13%), and last for “UGUAMM” (8%). We also revised the statements in manuscript, which is “The top five widely studied motifs in other species were selected and analyses using the DREME and MEME suites of in the mock-, RBSDV-, and RSV-infected rice samples were selected using the DREME and MEME suites (Supplementary Table S9), and the results showed that the most common motifs still were “RRACH” (68%), and “URUAY” (28%).”*

*Thanks very much for your suggestion again.*

*Reference:*

*[1]. Zhang F, Zhang YC, Liao JY, Yu Y, Chen YQ: The subunit of RNA N6-methyladenosine methyltransferase OsFIP regulates early degeneration of microspores in rice. PLoS Genet 2019, 15:e1008120.*

*[2]. Miao Z, Zhang T, Qi Y, Song J, Han Z, Ma C: Evolution of the RNA N6-methyladenosine methylome mediated by genomic duplication. Plant Physiol 2020, 182:345-360.*

*[3]. Arribas-Hernández L, Brodersen P: Occurrence and functions of m6A and other covalent modifications in plant mRNA. Plant Physiol 2020, 182:79-96.*

*[4]. Parker MT, Knop K, Sherwood AV, Schurch NJ, Mackinnon K, Gould PD, Hall AJW, Barton GJ, Simpson GG: Nanopore direct RNA sequencing maps the complexity of Arabidopsis mRNA processing and m6A modification. eLife 2020, 9:e49658.*

*[5]. Li Y, Wang X, Li C, Hu S, Yu J, Song S: Transcriptome-wide N6-methyladenosine profiling of rice callus and leaf reveals the presence of tissue-specific competitors involved in selective mRNA modification. RNA Biology 2014, 11:1180-1188.*

Minor point:

1. In line 174, the author calculated the correlation between the peak number and q-value. But in corresponding fig. 4B, the label is peak number and p-value. Which one is correct? In addition, please indicate what kind of p-value you used.

*Response: Thanks very much for your carefulness and profession. The Figure 4B presented was right, we determined the confidence of the peak significance through calculating the correlations of the peak numbers with the p-value/q-value simultaneously. Then, we obtained two maps that one is the peak numbers with the p-value, and one is the peak numbers with the q-value. We already have revised the sentence to “…correlations of the peak numbers and the p-value (Fig. 4B)”, and which was showed in the manuscript.*

*The p-value of m6A peak calling was calculated by MeTDiff software (2018- IEEE/ACM Transactions on Computational Biology and Bioinformatics). Briefly, the potential peak region is defined as consecutive bins predicted by the Viterbi. Its PeakScore was calculated, and the averaged PeakScores for all the combined bins. Next, the PeakScore is assumed to follow a gaussian distribution with mean [mean(PeakScore)] and standard deviation [std(PeakScore)], estimated from all the bins. Then, after performing the z-transform of the PeakScores, a one-sided test for significance of the potential peak region can be conducted and p-value can be calculated. Then, the Benjamini-Hochberg method is utilized to correct the multiple testing and compute the False Discovery Rate (FDR).*

*Thanks very much for your carefulness again.*

*Reference:*

*[1]. Cui X, Zhang L, Meng J, Rao MK, Chen Y, Huang Y: MeTDiff: A novel differential RNA methylation analysis for MeRIP-seq Data. IEEE/ACM Transactions on Computational Biology and Bioinformatics 2018, 15:526-534.*

2. line 180: The median number of the peak enrichment-fold was approximately 25-27. In the corresponding Fig. 4D, the label of y-axis is not consistent with 25-27 folds enrichment. Please check your calculation or label.

*Response: Thanks very much for your critical suggestion. Actually, we mistakenly evaluated the fold-enrichment number of the RBSDV_vs_mock and RSV_vs_mock in the manuscript. We checked the origin date of the Fig. 4D, and supplied the origin data this time, which was showed in Supplementary Table S18. The fold-enrichment were 25.33 and 26.25 for RSV- and RBSDV-infected samples, respectively. We already revised the sentence and the fold-enrichment number in the manuscript.*

*Thanks very much for your suggestion again.*

3. In Fig 4C, is y-axis number of genes or number of peaks?

*Response: Thanks very much for your suggestion. It’s wrong in the labeling of the y-axis. It’s the number of differential m6A peaks in the RBSDV_vs_mock and RSV_vs_mock. We already have revised the label of the Fig. 4C.*

*Thanks very much for your valuable suggestions again.*

4. The authors defined the high-expressed genes (FPKM ≥ 1) and genes which are not highly expressed (FPKM < 1) in line 193-194, but the authors further separated high-expressed genes into groups of FPKM = 1-5 and FPKM ≥ 5 in Fig. 5B without any explanation. It is quite confused when one reads line 192-200 regarding high- and low- expressed genes.

*Response: Thanks very much for your constructive suggestion. We already have revised the statements of the results of Fig. 5, especially the division of the genes.*

*Based on the confident sequencing results, the m6A peaks of each treatment were annotated to the genes. Rice genes that could be annotated by the m6A peaks were called m6A gene, and that couldn’t be annotated were called non-m6A gene in the next analyses. According to the Fragments Per Kilobase of exon model per Million mapped fragments (FPKM) (Supplementary Table S7), these genes were divided to three groups (FPKM < 1, 1 < FPKM < 5, and FPKM > 5), and the ratio of gene number of each category were calculated and showed by the heatmap (Fig. 5B). Either in the mock-treated sample or the viruses’ infected rice, most of the analysed genes (m6A or non-m6A genes) were mainly distributed in the low expressed category (FRKM < 1) (Fig. 5B). Compared to the mock-treated sample, the ratio of m6A methylated genes were increased in low expressed category of RSV- and RBSDV-infected samples (Fig. 5B). When these genes (m6A and non-m6A) were divided into highly expressed genes (FRKM ≥ 1) and genes that were not highly expressed (FRKM < 1), the expression of genes that was high or low were showed in mock-, RBSDV-, and RSV-infected samples (Fig. 5C), and we found that most genes were non-methylated and distributed in low expression category in mock-, RBSDV-, and RSV-infected rice samples (Fig. 5C). The m6A methylated genes were slightly enriched in the low expression category upon viruses’ infection of rice compared to mock-treated sample (Fig. 5C). Either in mock-treated or in RSV-infected and RBSDV-infected samples, the m6A-methylated genes were faintly expressed at a higher level than those of non-m6A methylated genes (Fig. 5D), and the highly expressed genes displayed a lower m6A occupancy (Fig. 5B). Hence, the m6A methylation mainly occurred in low expressed genes either in mock-treated or in RSV- and RBSDV-infected rice samples, and the m6A modified gene number were slightly enriched in low expressed category upon viruses’ infection.*

*Thanks very much for your valuable suggestion again.*

5. The authors mentioned to use mRNAs to perform LC-MS/MS and dot blot. What method did the authors use to isolate mRNAs? Please indicate in the Method section.

*Response: Thanks very much for your carefulness and profession. The mRNAs were isolated from the extracted total tissue RNAs by the the Ambion® Poly(A)Purist™ MAG kit (InvitrogenTM, USA). The mRNA that with poly(A) tails were enriched and isolated from total RNAs. We already added the detail information in the method section “Total mRNA isolation and Dot-blot analyses”, which was showed as “Total mRNA isolation and Dot-blot analyses. The extracted total RNAs of the rice plant by Trizol Reagent (TaKaRa Bio, Japan). Then, the Ambion® Poly(A)Purist™ MAG kit (InvitrogenTM, USA) was taken for total mRNA isolation according to the manufacturer's instructions. RNA integrity was evaluated using the Agilent 2100 Bioanalyzer (Agilent Technologies, Santa Clara, CA, USA)”.*

*Thanks very much for your valuable suggestion again.*

6. In Fig. 8B, please include a loading control to show the same amount of RNAs in Mock, RBSDV- and RSV-infected samples was loaded.

*Response: Thanks very much for your strictly design of the experiment, your suggestion will improve the manuscript a lot. According to your suggestion, we re-quantified the RNA concentration of the corresponding samples conserved in -80oC refrigerator, and added the loading control of Fig. 8B. To not affect the organization of Fig. 8, we added the loading control as Supplementary Figure S11, and the corresponding Figure legend and introduction were inserted in the manuscript.*

*Thanks very much for your constructive suggestion.*

7. The authors mentioned that OsALKBH9B-2 was up-regulated in RSV-infected samples. Where is OsALKBH9B-2 in Fig. 9B?

*Response: Thanks very much for your carefulness and profession. We already have revised the note of the Fig. 9B, and the accession number of OsALKBH9B-2 is Os12g0122000. We did not used the gene name in drawing the histogram. Thanks very much for your valuable suggestion again.*

8. There are lots of incorrect labeling in the figures. Please carefully check and correct them.

*Response: Thanks very much for your constructive suggestions. We already have checked the labeling appeared in the figures, and corrected them.*

(1) For example, in Fig. 4D, according to your y-axis, some of the fold-enrichment reach unbelievable fold change (2^(2^8) fold).

*Response: Thanks very much for your constructive suggestion. In Fig. 4D, the labeling of the y-axis have no problem. According to the Fig. 4D, the fold-enrichment were distributed mainly in 2^(21) to 2^(24), and the fold-enrichment of some individual free points reached to an unbelievable change that you thought. The violin plot of Fig. 4D showed that the fold-enrichment of most peaks were mainly distributed in the 2^(21) to 2^(24), and the distribution were relatively concentrated.*

*Thanks very much for your constructive suggestion again.*

(2) In Fig. 9 and 10, the authors did not mention what the meaning of a, b and c above the column is. These are only two examples, please check carefully through the whole ms.

*Response: Thanks very much for your constructive suggestion. According to your suggestion, we added the “Statistical analysis” part in the method section, and we also explained the meaning of different letters appeared in Fig. 9, Fig. 10, and from Fig. S3 to Fig. S9, and added the sentence “In each bar chart, different letters above bars denote statistically significant differences according to the Duncan's multiple range test of SPSS software (p < 0.05)” in the figure legend of these figures.*

*We checked the similar problems throughout the manuscript, and have revised these. Thanks very much for your valuable suggestion again.*

9. Line 136: Samples of these series libraries came from mock-, RSV-, and RBSDV-infected rice leaves at 60 dpt. However, the most severe symptoms are in 20- and 60 dpt respectively. Thus, the sampling timings do not catch the best time for one viral infection.

*Response: Thanks very much for your valuable suggestion. Yes, the symptoms that caused by RSV were severely at 60 dpt, and for RBSDV, the severe symptoms appeared at 20 dpt. The severe symptoms of RBSDV infection could last and usually not cause the plant death, and the symptoms could last for a long time, and RSV infection could cause two major symptoms: one is death and curling of the central leave of rice, the other one is milder, and could appeared mosaic ([1], [2], and [3]). Hence, to reduce the number of control (mock samples) and be more convenient of next sequencing data analyses, we determined the sampling timings to 60 dpt, and this long lasting infection of virus on plant, their m6A methylation dynamics were meaningful in interaction between plant virus-rice research.*

*Thanks very much for your constructive suggestion again.*

*Reference*

*[1]. Qiying Lin, Lianhui Xie, Zhongju Zhou, et. al. 1990. The research of the rice stripe virus I. The distribution and loss of the virus. Journal of Fujian Agricultural University, 19(4):421-425.*

*[2]. Dongming Gao, Wensheng Qin, Aiming Li, Hongfang Chen. 1993a. Relationship between different resistance rice cultivar and rice stripe virus disease. Rice science in China, 7(1):58-60.*

*[3]. Hanxin Lin, Taiyun Wei, Zujian Wu, Qiying Lin, Lianhui Xie. 2002. Comparisons of the chemical characteristics and pathogenicity of the seven rice stripe virus isolates in China. Journal of the Fujian Agricultural and Forest University (Natural Science), 31(2):164-167.*

10. Fig. 2 and 3: the better presentation way is needed: IP/Input.

*Response: Thanks very much for your constructive suggestion, your valuable comments will greatly improve the manuscript. We did not concentrate on some certain gene or genomic, our interest is to explore the m6A methylation dynamic in rice genome upon viruses’ infection. Hence, we think the Fig. 2 does not need to re-exhibition.*

*We already accepted your suggestions on Fig. 3, and re-exhibited the fine m6A peak distributions on the two viral genomic RNAs in Fig. 3C, Fig. 3D, and Supplementary Table S6.*

*The following content was added in the manuscript. “We also exhibited the fine m6A peaks that distributed to the each viral genomic RNAs (Supplementary Table S6), and the viral specific m6A peaks that distributed on RBSDV S5, S6, S9 (Fig. 3C) and RSV RNA1, RNA2, and RNA3 (Fig. 3D) were selected and exhibited. Our results suggested that the m6A modification often occurred in the 5’-terminal of the genomic RNAs of RBSDV, while they were random distributed on RSV genome. These maybe resulted from the characteristic of the two completely different plant viruses (RSV is a single-strand RNA virus, while RBSDV is a double-stranded RNA virus).”*

*Thanks very much for your critical suggestions again.*

11. m6A location: 5'UTR, Coding-sequence or 3'UTR? There are many conflicted statements through the ms. Line 480: most m6A sites were located in the CDS region? What about in Fig. 6A?

*Response: Thanks very much for your constructive suggestion, we have completely accepted your suggestions and revised the corresponding statements throughout the manuscript.*

(1) Line 480: most m6A sites were located in the CDS region? What about in Fig. 6A?

*Response: Thanks a lot. Fig. S10E and Fig. S10F exhibited the results of integrated analyses of the m6A methylation peaks position on gene body and their expression profile of the target gene. Hence, we firstly combined the RNA-seq transcriptome data with the m6A-IP sequencing data, and association analyses was performed. The genes simultaneously appeared in these two groups were selected, and their relationships between m6A methylation position and their corresponding expression profile of target gene were summarized.*

*While, Fig. 6A showed the total m6A peak distribution pattern from m6A-IP sequencing data, and represent the whole m6A distribution pattern in gene body. These two figures demonstrated the two aspects of m6A peaks, and were two different things.*

*Thanks very much for your valuable suggestion.*

**Second round of review**

**Reviewer 1**

**The authors fully addressed my concerns.**

**Reviewer 2**

**The authors addressed most of the questions. However, the following questions should be further addressed:**

**1. In line 650-653, "The m6A modifications were strongly associated with the up- or down-regulation of the target genes, which determined the direct correlation between m6A modification and relative expression.", I cannot see the strong association between expression level and m6A modification based on Fig. 8C and 8D. As the authors mention, the relative expression pattern of OsAGO18 (Increased) and OsSLRL1 (Decreased) were completely opposite to each other.**

**2. The authors mentioned several times in the response that the different m6A methylation sites have different effects to the expression pattern. Please check carefully the methylation sites on these genes such as OsAGO18, OsSLRL1, MTA, MTB, YTH etc.**

**Authors Response**

**Point-by-point responses to the reviewers’ comments:**

Reviewer #1:

1. The authors fully addressed my concerns.

*Response: Thanks very much for your encouragement, patience, and professionalism.*

Reviewer #2:

1. The authors addressed most of the questions. However, the following questions should be further addressed:

In line 650-653, "The m6A modifications were strongly associated with the up- or down-regulation of the target genes, which determined the direct correlation between m6A modification and relative expression.", I cannot see the strong association between expression level and m6A modification based on Fig. 8C and 8D. As the authors mention, the relative expression pattern of OsAGO18 (Increased) and OsSLRL1 (Decreased) were completely opposite to each other.

*Response: Thanks very much for your critical and constructive suggestion. In Fig. 8C and 8D, the RT-qPCR were used for tracing the dynamic expression levels of OsAGO18 and OsSLRL1 after RBSDV and RSV infected for 1-, 2-, 4-, 8-, and 16-day, respectively. Through these two genes’ expression level, we known that the OsAGO18 was induced gradually with RBSDV and RSV infection, whereas the OsSLRL1 was suppressed.*

*Furthermore, we wonder if these two genes’ expression profile were correlated to their m6A methylation. According to the m6A-sequencing result, we known that OsAGO18 and OsSLRL1 occurred m6A methylation modifications. To clear the detail m6A methylation region on these two genes, we designed 12 and 10 pairs of primers corresponding to the specific regions on OsAGO18 and OsSLRL1 gene body respectively. The corresponding detail region of each pair of primers was showed in Additional file 2: Table S3. Then, the m6A-IP were performed, resins that bounded with anti-m6A antibody (#ab208577; Abcam) were incubated with total mRNAs (Fragmented) of rice samples with or without viruses’ infection at 1-, 2-, 4-, 8-, and 16-day, respectively. All the m6A methylated genes could be precipitated, and these precipitated m6A genes were used as total RNAs and qRT-PCR were performed to evaluate the m6A methylation levels of different regions on target genes. As shown in Fig. 8E, the m6A levels were gradually increased on OsAGO18 position 6 (1,200-1,374 bp) with RBSDV infected for 1-, 2-, 4-, 8-, and 16-day, or on OsAGO18 position 12 (3,607-3,784 bp) with RSV infected for 1-, 2-, 4-, 8-, and 16-day. While, the m6A levels were not altered on OsAGO18 position 1 (110-258 bp) with RSV and RBSDV infected for 1-, 2-, 4-, 8-, and 16-day.*

*From the gene’s structure, we known that position 1 was located in 3’-UTR, position 6 was located in coding region, and position 12 was located in 5’-UTR. Combination analyses of the results of Fig. 8C and Fig. 8E, we could easily find the correlations between m6A levels and their total expression levels. In RBSDV-infected sample, the increased m6A levels on coding region of OsAGO18 were tightly and positively correlated with its expression profile at virus infected for 1-, 2-, 4-, 8-, and 16-day. In RSV-infected sample, the increased m6A levels on 5’-UTR were also tightly and positively correlated with its expression profile at virus infected for 1-, 2-, 4-, 8-, and 16-day.*

*Similar to the analyses process of OsAGO18, the results of Fig. 8D and Fig. 8F were integrated and further analyzed. Three coding regions of OsSLRL1, which including position 2 (161-341 bp), position 3 (372-525 bp), and position 4 (538-757 bp), were selected showed. In RBSDV-infected sample, the increased m6A levels on position 2 and 4 were tightly and negatively correlated with its expression profile at virus infected for 1-, 2-, 4-, 8-, and 16-day, while m6A levels on position 3 was not correlated with its expression profile. In RSV-Infected samples, the increased m6A levels on position 3 were tightly and negatively correlated with its expression profile at virus infected for 1-, 2-, 4-, 8-, and 16-day, while m6A levels on position 2 and 4 were not corrected with its expression profile.*

*To sum, we conclude the findings and given the result “these collective findings indicated that changes in the relative expression of OsAGO18 and OsSLRL1 tightly correlated with the changes in their m6A modification level and that the m6A methylation of different gene regions may have different effects on its expression”.*

*And we also added the Figures at correct sites in manuscripts. Which are showed “The m6A modifications were strongly associated with the up- (Fig. 8C and 8E) or down-regulation (Fig. 8D and 8F) of the target genes, which determined the direct correlation between m6A modification and relative expression.”*

*Thanks very much for your valuable concern and professional spirits in handling our manuscript again.*

2. The authors mentioned several times in the response that the different m6A methylation sites have different effects to the expression pattern. Please check carefully the methylation sites on these genes such as OsAGO18, OsSLRL1, MTA, MTB, YTH etc.

*Response: Thanks very much for your professionalism and constructive suggestions. As you suggested, we re-checked the m6A methylation status and their expression profiles of OsAGO18, OsSLRL1, MTA, MTB, YTH etc.*

*For OsAGO18 and OsSLRL1, we have already discussed above. These two genes are selected as representatives, and we performed m6A-IP-qPCR validated the credibility of our m6A-IP-sequencing data with different pairs of primers. The sequencing results and our experiment indicates “changes in the relative expression of OsAGO18 and OsSLRL1 tightly correlated with the changes in their m6A modification level and that the m6A methylation of different gene regions may have different effects on its expression”.*

*For MAT and YTH, we just determined their relative expression levels with or without two rice viruses’ infection, and not determined its specific m6A regions of each gene using experiment (m6A-IP-qPCR). From the m6A-IP-sequencing data, we known that the OsMAT3 was m6A methylated in RBSDV-infected plant, and OsMAT4 was m6A methylated in RSV-infected plant, while both of their expression levels were not changed. The m6A methylation statues and their expression levels are similar to genes of OsYHT1, OsYHT10, OsYHT11, and OsYHT12 in RBSDV-infected plants. While in RSV-infected plant, the m6A methylation of OsYHT1 and OsYHT10 correspond to suppressed expression. Hence, there are no conserved regulation modes. According to the validation process of OsAGO18 and OsSLRL1, we have predicted that different m6A methylation sites have different effects to the expression pattern.*

*About the real effect of m6A methylation to their expression profile of each gene your suggested, we will firstly to determine the specific regions on gene body using m6A-IP-qPCR, then to analyses the location of the m6A methylation sites, and we looking forward to find some common and conserved regulation modes in future work.*

*Thanks very much for your constructive suggestions again.*
